# Luminescent Au(III)–M(I) (M = Cu, Ag) Aggregates Based on Dicyclometalated Bis(alkynyl) Gold Anions†

**DOI:** 10.1021/acs.inorgchem.3c00870

**Published:** 2023-08-03

**Authors:** Rebeca Lara Garnica, Raquel J. Rama, Isabelle Chambrier, Gabriele Agonigi, David L. Hughes, Elena Lalinde, Manfred Bochmann, Julio Fernandez-Cestau

**Affiliations:** ‡ School of Chemistry, University of East Anglia, Norwich Research Park, Norwich NR4 7TJ, U.K.; § Departamento de Química—Centro de Investigación en Síntesis Química (CISQ), Universidad de La Rioja, E-26006 Logroño, Spain; ∥ Dipartimento di Chimica e Chimica Industriale, University of Pisa, I-56124 Pisa, Italy; ⊥ Departamento de Química Inorgánica, Universidad de Sevilla, 41071 Sevilla, Spain; # SMN Centre for Materials Science and Nanotechnology, Department of Chemistry, University of Oslo, Sem Sælands vei 26, 0371 Oslo, Norway

## Abstract

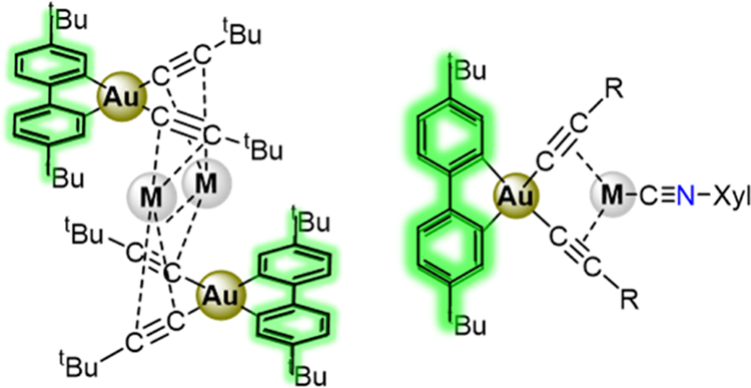

The syntheses and structures of a series of complexes based on the C^∧^C-chelated Au(III) unit (C^∧^C = 4,4′-bis(*t*-butyl) 2,2′-biphenyl-1,1′-diyl) are reported, namely, [{(C^∧^C)Au(C≡C*^t^*Bu)_2_}_2_M_2_], (C^∧^C)Au(C≡CR)(C≡NXyl), and [{(C^∧^C)Au(C≡CR)_2_}{M(C≡NXyl)}] (M = Ag, Cu; R = *^t^*Bu, C_6_H_4_*^t^*Bu-4, C_6_H_4_OMe-4; Xyl = 3,5-Me_2_C_6_H_3_). The X-ray structures reveal a broad range of dispositions determined by the different coordination modes of Ag(I) or Cu(I). The complexes are bright photoemitters in the solid state and in poly(methyl methacrylate) (PMMA) films. The photoluminescence is dominated by ^3^IL(C^∧^C) transitions, with indirect effects from the rest of the molecules, as supported by theoretical calculations. This work opens up the possibility of accessing Au(III) carbon-rich anions to construct photoluminescent aggregates.

## Abstract 2

The synthesis and structures of a series of polymetallic complexes based in a C^∧^C-chelated Au(III) unit of the types [{(C^∧^C)Au(C≡C*^t^*Bu)_2_}_2_M_2_], (C^∧^C)Au(C≡CR)(C≡NXyl), [{(C^∧^C)Au(C≡CR)_2_}{M(C≡NXyl)}] (C^∧^C = 4,4′-bis(*t*-butyl) 2,2′-biphenyl-1,1′-diyl) (M = Ag, Cu; R = *^t^*Bu, C_6_H_4_*^t^*Bu-4, C_6_H_4_OMe-4; Xyl = 3,5-Me_2_C_6_H_3_) are reported. The complexes are phosphorescent with lifetimes of up to 50 μs predominantly due to ^3^IL(C^∧^C) transitions.

## Introduction

Phosphorescent organic light-emitting diodes (OLEDs) have been traditionally dominated by platinum(II)^1−5^ and iridium(III)^6−8^ complexes as photoemitters. However, in recent years, gold(III) complexes, particularly those based on 2-arylpyridine (C^∧^N) and 2,6-biphenylpyridine (C^∧^N^∧^C) pincer ligands as chromophores, have emerged as excellent alternatives.^9−11^ These ligands have been found to be effective platforms for stabilizing Au(III) against reduction^12−14^ and, at the same time, generate emissive compounds. Their luminescent properties are generally ^3^LC in nature and, in some cases, show TADF (thermally activated delayed fluorescence) behavior.^15−18^ The emission is favored by the coordination of mono- or di-cyclometallated ligands and the high ligand-field splitting thus induced, which, upon photoexcitation, avoids the population of metal-centered d-states typically responsible for nonradiative relaxation processes. To further enhance this effect, the remaining coordination positions are typically occupied by strong C-based σ-donor ligands, such as alkyl,^16^ aryl,^17,19−21^ alkynyl,^15,19,22,23^*N*-heterocyclic carbenes,^19^ or thiolates.^24^

However, the strategy of introducing high ligand-field splitting in such complexes has limitations due to the presence of the central pyridine ring of the cyclometallated ligand. We and others have demonstrated that modifying the central ring in the C^∧^N^∧^C fragment significantly changes the photophysical properties of the synthesized complexes.^15,20^ Furthermore, Nevado et. al. reported in 2015 the (C^∧^C^∧^N) analogue of the (C^∧^N^∧^C) pincer.^25^ Introducing a C(sp^2^) donor in place of the N atom of the pyridine increases the trans influence and makes the fluorine complex suitable as starting material for direct substitution by aryls and alkynes, among others. These complexes have been implemented by several authors in efficient OLEDs.^19,22,26^

Nevertheless, in these C^∧^C^∧^N complexes, like for C^∧^N and C^∧^N^∧^C, one position in the square-planar coordination environment of Au(III) is still occupied by the pyridine. For this reason, the introduction of dianionic chelating C-donor ligands, with no assistance by an *N*-heterocycle donor, is obviously attractive. The first examples of this ligand in gold chemistry date back to Usón et al. in the 1980s, who reported 1,2,3,4-tetraphenyl-butadien-1,4-diyl and 2,2′-biphenyl-1,1′-diyl complexes of gold(III).^27^ More recently, Mohr and co-workers prepared a series of *t*-butyl substituted 2,2′-biphenyl-diyl compounds, with improved solubility and potentially interesting photophysical properties.^28^ In recent years, a series of biphenyl-derived C^∧^C gold(III) complexes with promising photoluminescence have been reported^29−31^ and one cationic 4,4′-di-tert-butyl-1,1′-biphenyl Au(III) diphosphine complex has been already employed as electroactive complex in the first light-emitting electrochemical cell (LECs) device based on this element.^32^

These ligands have proved their ability to stabilize gold(III) complexes by a combination of good electron-donor properties, and π-acceptor capability with steric rigidity. This provides excellent protection against reduction. We therefore became interested in these C^∧^C ligands when searching for stable gold(III) alkene and alkyne complexes^12,13,33^ and found that 4,4′-bis(*t*-butyl)-2,2′-biphenyl-1,1′-diyl ligands allow the synthesis and crystallographic characterization of thermally stable alkene complexes of the type [(C^∧^C)Au(diene)]^+^ (diene = 1,5-cyclooctadiene or norbornadiene)^34^ and also provided access to an unprecedented series of neutral and anionic gold(III) hydrides.^35^

As part of our search for photoluminescent gold(III) chelate and pincer complexes,^15,16,24,36^ we turn our attention to the study of luminescent gold(III)–M(I) aggregates.^37^ It is known that the formation of heteronuclear aggregates through supported or unsupported intermetallic contacts, mainly of heavy-metal atoms [Pt(II), Au(I), Ag(I), Cu(I)], not only induces intriguing photoluminescent behavior, due to the presence of noncovalent interactions, that on occasions are sensitive to external stimuli, but also facilitates S_1_ to T_1_ promotion, thus enhancing the phosphorescence radiative relaxation.^38−41^ In addition, the molecular rigidity in the final arrays is usually increased in relation to their mononuclear counterparts, and therefore nonradiative deactivation of triplet excited states is distinctly reduced.^42^

In this context, we describe here the synthesis and structures of a number of mono- and polynuclear C^∧^C-based (C^∧^C = 4,4′-bis(*t*-butyl) 2,2′-biphenyl-1,1′-diyl) gold(III) complexes, including alkynyl-bridged silver and copper aggregates. The photophysical properties of these compounds are also investigated and their origin is supported by computational studies.

## Results and Discussion

### Synthesis and NMR Characterization

**Scheme 1:**
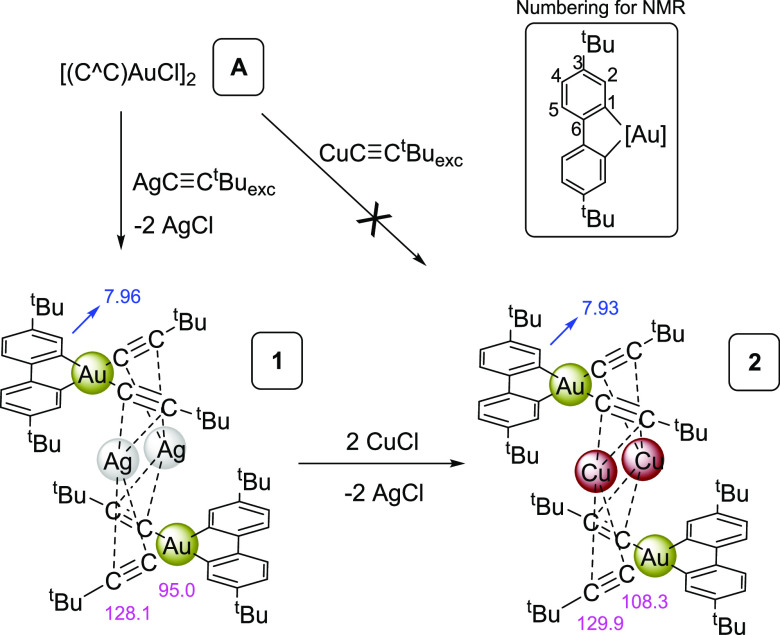
Synthesis of Complexes [{(C^∧^C)Au(C≡C*^t^*Bu)_2_}_2_Ag_2_] **1** and [{(C^∧^C)Au(C≡C*^t^*Bu)_2_}_2_Cu_2_] **2** with Selected ^1^H NMR (Blue) and ^13^C{^1^H} (Pink) NMR Chemical Shifts Inset shows the numbering used for the NMR signals in both complexes.

Treatment of the (poorly soluble) chloride complex **A**(28) with silver *tert*-butyl acetylide caused a double alkynylation process, with precipitation of AgCl, giving rise to a yellow solution from which the cluster [{(C^∧^C)Au(C≡C*^t^*Bu)_2_}_2_Ag_2_] **1**, was isolated as yellow crystals (Scheme 1). Complex **1** shows a simple ^1^H NMR pattern that confirms the introduction of two acetylides *per* C^∧^C fragment and the equivalence of both rings of the C^∧^C ligand. Based on X-ray crystallography, the complex is a tetranuclear aggregate [{(C^∧^C)Au(C≡C*^t^*Bu)_2_}_2_Ag_2_] **1**, in which two [(C^∧^C)Au(C≡C*^t^*Bu)_2_]^−^ anions stabilize two Ag(I) ions by coordination of each silver to two alkynyls, one from each gold fragment, acting as η^2^-C≡C···Ag bridging ligands. The related tetranuclear cluster [{(C^∧^C)Au(C≡C-*^t^*Bu)_2_}_2_Cu_2_] **2** was easily obtained by a metathesis reaction. Thus, treatment of a solution of **1** in CH_2_Cl_2_ with two equivalents of CuCl caused the precipitation of AgCl and the formation of **2** in solution. The insolubility of AgCl and the stability of the final aggregates clearly act as the driving force of the formation of both complexes. Indeed, complex **2** is not accessible by direct treatment of **A** with CuC≡C*^t^*Bu, which underlines the key role of the formation of AgCl precipitate in both reactions.

The ^13^C{^1^H} NMR spectra are particularly diagnostic. Specifically, the silver complex [{(C^∧^C)Au(C≡C-*^t^*Bu)_2_}_2_Ag_2_] **1** shows a distinct set of signals for C^α^ and C^β^ (C^α^≡ C^β*t*^Bu) at 95.0 and 128.1 ppm, respectively, appearing as broad doublets due to coupling to ^107^Ag and ^109^Ag atoms. The fact that the signals appear as two doublets without resolution is indicative of the tetranuclear aggregate’s permanence in the solvent. This is because the NMR signal reflects an admixture of three isotopomers, specifically ^107^Ag^107^Ag, ^109^Ag^109^Ag, and ^107^Ag^109^Ag, in an approximate 1:1:2 ratio. The ^13^C{^1^H} NMR spectrum of the complex [{(C^∧^C)Au(C≡C*^t^*Bu)_2_}_2_Cu_2_] **2** displays singlets at 129.9 ppm (C^β^) and 108.3 ppm (C^α^).

**Scheme 2:**
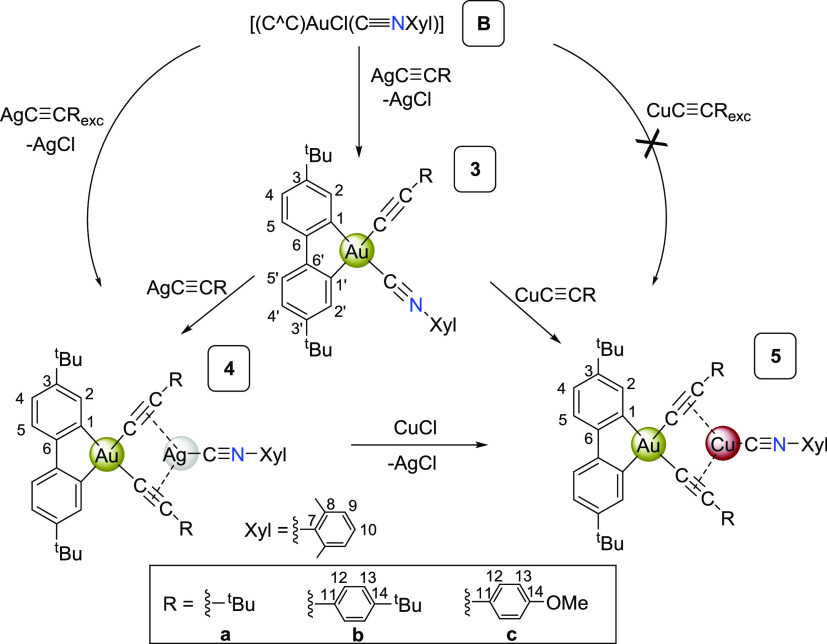
Synthesis of Complexes (C^∧^C)Au(C≡CR)(C≡NXyl) (R = *^t^*Bu **3a**, C_6_H_4_*^t^*Bu-4 **3b**, C_6_H_4_OMe-4 **3c**) [{(C^∧^C)Au(C≡CR)_2_}{Ag(C≡NXyl)}] (R = *^t^*Bu **4a**, C_6_H_4_*^t^*Bu-4 **4b**, C_6_H_4_OMe-4 **4c**) and [{(C^∧^C)Au(C≡CR)_2_}{Cu(C≡NXyl)}] (R = *^t^*Bu **5a**, C_6_H_4_*^t^*Bu-4 **5b**, C_6_H_4_OMe-4 **5c**)

In 2014, Mohr^28^ described the synthesis of the mononuclear isocyanide adduct [(C^∧^C)AuCl(CNXyl)] (**B**, Scheme 2). Treatment of this complex with one equivalent of silver acetylide in CH_2_Cl_2_ results in the slow precipitation of AgCl and the formation of the corresponding mixed isocyanide/acetylide complexes (C^∧^C)Au(C≡CR)(C≡NXyl) (R = *^t^*Bu **3a**, C_6_H_4_*^t^*Bu-4 **3b**, C_6_H_4_OMe-4 **3c**). Treatment of complexes **3** with a second equivalent of silver acetylide, or direct treatment of the precursor [(C^∧^C)Au(CNXyl)(C≡CR)] (**B**) with an excess of the corresponding AgC≡CR, in CH_2_Cl_2_, evolve with the formation of a series of complexes of stoichiometry [{(C^∧^C)Au(C≡CR)_2_}{Ag(C≡NXyl)}]*_x_* (*X* = 1, R = *^t^*Bu, **4a**, C_6_H_4_*^t^*Bu-4; **4b**; *X* = 2, R C_6_H_4_OMe-4, **4c**). In these complexes, the bis-acetylide anions [(C^∧^C)Au(C≡CR)_2_]^−^ act as chelating ligands toward a [Ag(C≡NXyl)]^+^ cation by η^2^-C≡C bonding, as confirmed by X-ray diffraction studies on **4a** and **4c**.

The formation of these complexes involves a double migration process with the isocyanide ligand transferring from gold to silver and the acetylide ligand from silver to gold. Alkynyl to chloride exchange and alkynylation processes have been previously reported for the preparation of heterometallic complexes featuring a *cis*-[M](C≡CR)_2_ unit acting as a chelating ligand toward a second metal.^43^ Not only Ag(I) but also Cu(I) and Au(I) metal acetylides (MC≡CR)*_x_* (M = Cu, Ag, Au) have been extensively used for constructing polymetallic luminescent systems.^38,44^ However, as far as we know, the complexes reported here are the first examples in gold chemistry featuring a *cis-* “Au(C≡CR)_2_” chelating disposition to any other metal. As shown in Scheme 2, similar isocyanide/alkynyl exchange process takes place by reaction of complex **3** with the corresponding CuC≡CR giving rise to the related Au(III)–Cu(I) series [{(C^∧^C)Au(C≡CR)_2_}{Cu(C≡NXyl)}] (R = *^t^*Bu **5a**, C_6_H_4_*^t^*Bu-4 **5b**, C_6_H_4_OMe-4 **5c**). These complexes (**5a**–**c**) can also be easily prepared by treatment of the corresponding Au(III)–Ag(I) aggregate (**4a–c**) with one equivalent of CuCl. Once again, the precipitation of AgCl is the driving force for the reaction and attempts to react **B** with copper acetylides (1:1 molar ratio or excess) result in admixtures that could not be identified.

Unlike the remarkable stability of mononuclear (**3**) and binuclear Au(III)–Ag(I) (**4)** complexes, the related Au(III)-Cu(I) derivatives (**5)** slowly lose the isocyanide ligand in solution. In fact, complex **2** was first detected in our attempts to grow crystals of **5a**, from which **2** crystallized as a byproduct. This is most likely due to the weaker nature of the Cu(I)-isocyanide bond compared to that of Ag(I), a consequence of the lesser back-donation component.

The ^1^H NMR spectra of complexes **3** show two signals corresponding to H^2^ and H^2′^, indicating the lack of symmetry in the C^∧^C ligand. By contrast, the binuclear complexes Au(III)–M(I) (**4** and **5**) exhibit a single signal for H^2^, integrating for two protons, due to the recovery of the symmetry plane.

The ^13^C{^1^H} NMR chemical shift of the acetylide C^α^≡C^β^ signals changes remarkably in the binuclear compounds **4** and **5** compared to the corresponding mononuclear precursor **3**. Thus, in **3a**, having the *tert-*butyl acetylide bonded in a terminal fashion (κ^1^:C^α^), both signals appear close to each other (δ 114.7 C^β^, 101.2 C^α^). In contrast, in **4a** and **5a**, where the alkynyls exhibit a μ-κC^α^:η^2^ bonding, the C^β^ shifts to higher and the C^α^ to lower frequencies (δ 124.9 C^β^, 87.3 C^α^**4a** and 126.4 C^β^ 87.7 C^α^**5a**). This trend is also observed for the **b** and **c** series and is consistent with observations in other polymetallic systems with bridging η^2^ alkynyls.^43^

### X-ray Diffraction Studies

**Figure 1:**
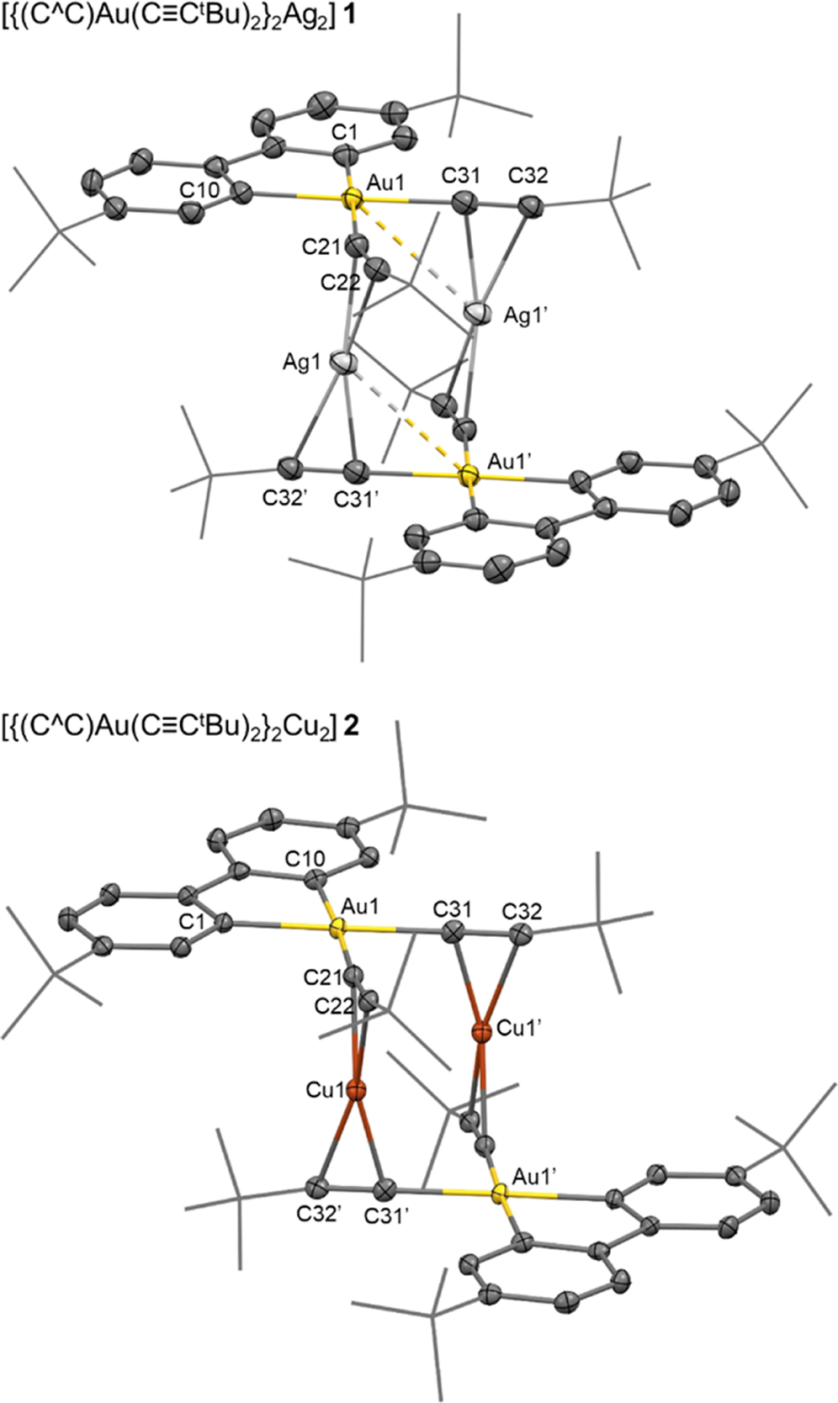
X-ray structure of [{(C^∧^C)Au(C≡C*^t^*Bu)_2_}_2_Ag_2_] **1** (top) and [{(C^∧^C)Au(C≡C*^t^*Bu)_2_}_2_Cu_2_] **2** (down). The structures are shown as based skeleton with the most relevant atoms represented as ellipsoids with 50% probability level. Selected bond distances (Å) and angles (°): **Complex 1**: Au1–C1 2.045(2), Au1–C11 2.045(3), Au1–C21 2.063(2), C21–C22 1.212(3), Au1–C31 2.058(3), C31–C32 1.210(4), Ag1–C21 2.204(2), Ag1–C22 2.379(2), Ag1–C31′ 2.214(2), Ag1–C32′ 2.392(2), Ag1···Au1′ 3.2155(6), Ag1···Au1 3.2982(4). C1–Au1–C10 81.6(1), C1–Au1–C31 94.5(1), C11–Au1–C21 97.1(1), C21–Au1–C31 86.7(1), Au1–C21–C22 164.3(2), Au1–C31–C32 174.8(2), C21–C22–C23 168.4(3), C31–C32–C33 168.4(3), C21–Ag1–C31′ 163.53(9). **Complex 2**: Au1–C1 2.042(3), Au1–C10 2.044(3), Au1–C21 2.053(3), C21–C22 1.223(4), Au1–C31 2.054(3), C31–C32 1.228(5), Cu1–C21 1.997(2), Cu1–C22 2.103(3), Cu1–C31′ 1.999(3), Cu1–C32′ 2.108(3). C1–Au1–C10 81.3(1), C1–Au1–C31 95.0(1), C10–Au1–C21 97.2(1), C21–Au1–C31 86.5(1), Au1–C21–C22 165.4(3), Au1–C31–C32 174.1(3), C21–C22–C23 165.6(3), centroid(C21–C22)-Cu1-centroid(C31′–C32′) 177.5.

Light yellow crystals of [{(C^∧^C)Au(C≡C*^t^*Bu)_2_}_2_Ag_2_] **1** were obtained by slow diffusion of pentane into a solution of the complex in CH_2_Cl_2_. Colorless crystals of complex[{(C^∧^C)Au(C≡C*^t^*Bu)_2_}_2_Cu_2_] **2** were grown by slow evaporation of a CH_2_Cl_2_ solution of the crude product. Representations of both structures are presented in Figure 1.

As depicted in Figure 1, both structures confirm the tetranuclear nature of the complexes. Specifically, each complex consists of two square-planar (C^∧^C)Au(C≡C*^t^*Bu)_2_ fragments connected by two M(I) (M = Ag **1**, Cu **2**) ions. The main difference between the two structures lies in how the M(I) ion coordinates to the alkynyl groups. As previously noted, the M(I) ions are stabilized by two η^2^-C≡C···M(I) alkynyl ligands, one from each Au(III) fragment, resulting in a linear coordination environment for both the Ag(I) and the Cu(I) centers.

In complex **1**, the coordination of the silver centers to both alkynyls is slightly asymmetrical, as evidenced by shorter Ag–C^α^ distances (2.204(2) and 2.214(2) Å) compared to Ag–C^β^ distances (2.379(2) and 2.392(2) Å). Interestingly, the Ag1–Au1′ distance of 3.2155 (6) Å is shorter than the sum of the van der Waals radii of both atoms (1.66 + 1.72 = 3.38 Å). This fact points to the possibility of bonding interaction between Au(III) and Ag(I). Although profusely studied for Au(I), the ability of the smaller electropositive Au(III) to form metallophilic interactions is a long kept debate since the first calculations of Pyykko and Mendizabal.^39^ Recent studies by Che and co-workers point to the existence of very weak Au(III)–Au(III) bonding interactions, in cyclometalated gold(III) complexes.^45^ We reported in 2017^13^ one mixed Au(III)_2_Au(I)Ag(I) complex, related to **1**, where the Ag(I) center was stabilized through a double asymmetric η^2^-C≡C bridge toward two “(C^∧^C)Au(C≡C*^t^*Bu)” fragments (Ag–C^α^ 2.24–2.25 Å, Ag–C^β^ 2.32–2.35 Å). However, in that case, the Au(III)–Ag(I) distances of 3.422 and 3.493 Å were found to be much longer than those described for **1** and longer than the sum of the van der Waals radii. Given that these two structures are very similar in nature but taking into consideration that dispersion forces have been overestimated in the past and that electrostatics and orbital interactions tend to be dominant, especially in hetero-bimetallic systems,^46^ we suggest that the short Au(III)–Ag(I) distance in **1** is likely due to electrostatic interactions originating from the presence here of the electron-rich [(C^∧^C)Au(C≡C*^t^*Bu)_2_]^−^ anion.

Similarly, the Cu(I) centers in complex [{(C^∧^C)Au(C≡C*^t^*Bu)_2_}_2_Cu_2_] **2** coordinate to the alkynyls in a slightly asymmetrical manner, with Cu1–C^α^ distances (1.999(3) Å; 1.997(2) Å) shorter than Cu1–C^β^ (2.108(3) Å; 2.103(3) Å). The Cu-alkynyl distances are shorter than the Ag-alkynyl distances in complex **1**, as previously found in related systems.^47^ This leads to a shorter distance between the coordination planes of the Au fragments in **2** (3.81 Å) compared to **1** (4.35 Å). Furthermore, the shift, measured as the distance between the gold centers parallel to the coordination plane of one of them (see Figure S2-1), is smaller in **1** (3.40 Å) than in **2** (3.93 Å). This shift may be attributed to the Cu-alkyne distances being shorter than the Ag-alkyne. This forces the Au(III) fragments to sit closer in **2** and produces larger steric repulsions that promote relaxation of the structure by increasing the displacement.

**Figure 2:**
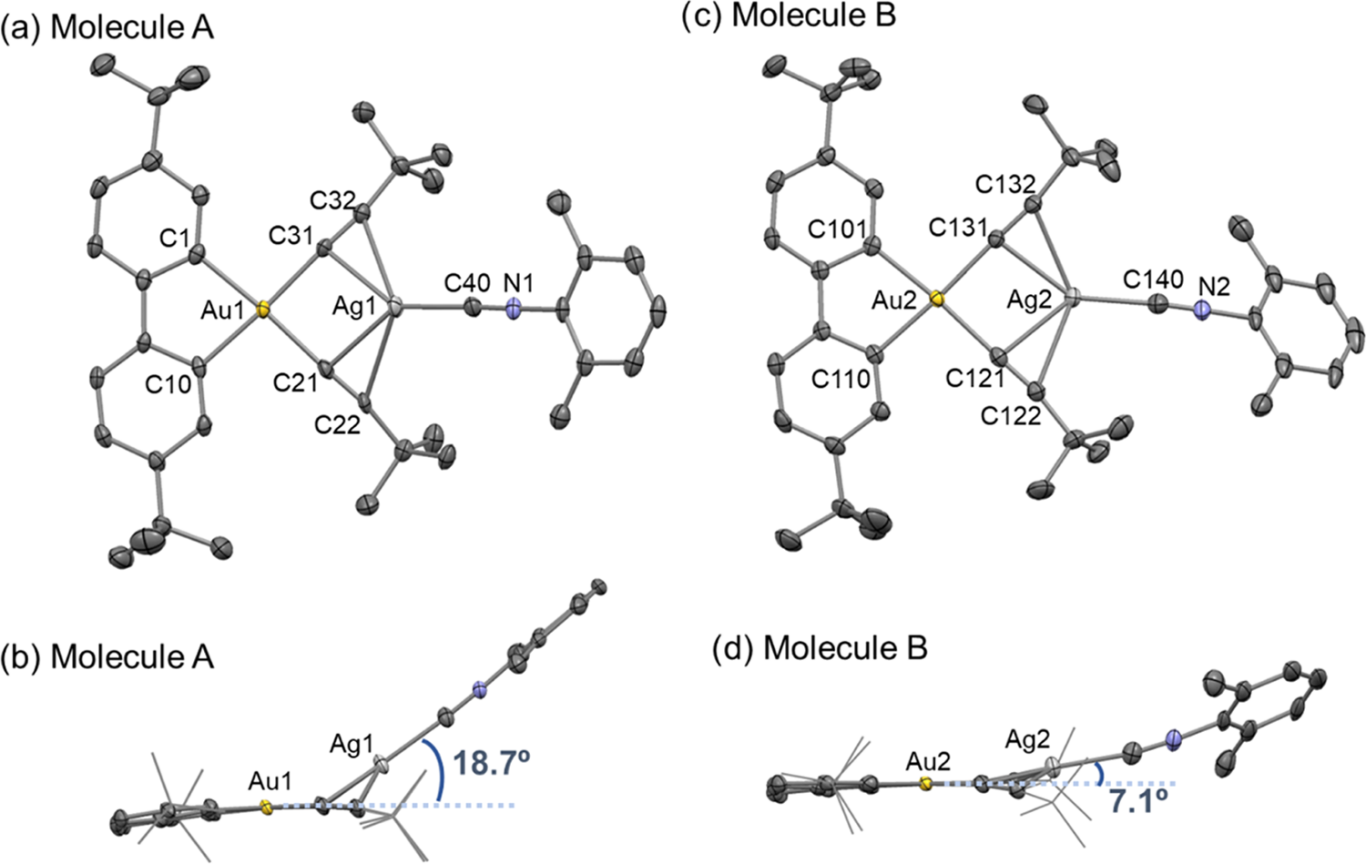
X-ray structure of [{(C^∧^C)Au(C≡C*^t^*Bu)_2_}{Ag(C≡NXyl)}] **4a**. Ellipsoids with a 50% probability level. The unit cell shows two different molecules; Molecule A (a, b) and Molecule B (c, d). Selected bond distances (Å) and angles (°): **Molecule A**: Au1–C1 2.035(4), Au1–C10 2.040(3), Au1–C21 2.047(4), C21–C22 1.217(6), Au1–C31 2.049(3), C31–C32 1.214(4), Ag1–C21 2.379(3), Ag1–C22 2.605(3), Ag1–C31 2.384(4), Ag1–C32 2.600(4), Ag1–C40 2.095(4), C40–N1 1.146(6), Au1···Ag1 3.2279(7), C1–Au1–C10 81.5(2), C1–Au1–C31 95.0(2), C10–Au1–C21 94.7(1), C21–Au1–C31 88.6(1), centroid(C21–C22)–Ag1-centroid(C31–C32) 98.5, centroid(C21–C22)–Ag1–C40 128.34, centroid(C31–C32)–Ag1–C40 131.53. **Molecule B**: Au2–C101 2.034(4), Au2–C110 2.047(3), Au2–C121 2.059(5), C121–C122 1.230(6), Au2–C131 2.051(3), C131–C132 1.227(5), Ag2–C121 2.404(3), Ag2–C122 2.540(3), Ag2–C131 2.359 (4), Ag2–C132 2.478(4), Ag2–C140 2.113(5), C140–N2 1.145(6), Au2···Ag2 3.3434(7), C101–Au2–C110 81.4(1), C101–Au2–C131 94.4(1), C121–Au2–C131 89.0(2), C110–Au2–C121 95.3(2), centroid(C121–C122)–Ag2-centroid(C131–C132) 103.20, centroid(C121–C122)–Ag2–C140 123.40, centroid(C131–C132)–Ag2–C140 132.51.

Crystals of [{(C^∧^C)Au(C≡C*^t^*Bu)_2_}{Ag(C≡NXyl)}] **4a** were grown by slow diffusion of light petroleum into a saturated solution of the complex in CD_2_Cl_2_. The structure confirms the formulation as a bimetallic complex in which the anion [(C^∧^C)Au(C≡C*^t^*Bu)_2_]^−^ stabilizes the cation [Ag(C≡NXyl)]^+^ by forming two asymmetric η^2^-C≡C···Ag bridges (Ag–C^α^ 2.359(4)–2.404(3) Å; Ag–C^β^ 2.478(4)–2.540(3) Å). As shown in Figure 2, the asymmetric unit comprises two different types of molecules (named A and B). The main difference between them is the position of the Ag(C≡NXyl) fragment relative to the coordination plane of gold. In both cases, the Ag(I) ions are in a trigonal coordination environment by bonding to the isocyanide and the two alkynyls (defined by the centroid C^α^≡C^β^). Molecule A has a V-shaped coordination supplementary angle to torsion with an angle between the Au–Ag vector and the coordination plane of Au of 18.7°, while Molecule B has a more in-plane arrangement of 7.1°.

The Au1–Ag1 distance (3.2279(7) Å) in Molecule A, is only slightly longer than the Au–Ag distance found in complex 1 (3.2155(6) Å) and still shorter than the sum of the van der Waals radii of both atoms (3.38 Å). The most plausible explanation for the V-shape disposition in Molecule A that determines the gold–silver distance, is the presence of intermolecular interactions. In fact, as can be seen in Figure S2-2, the Ag centers of both molecules complete their electronic requirements by interaction with a carbon atom of a vicinal molecule (Ag1···C106 3.270 Å, Ag2···C43 3.149 Å) forming a dimeric aggregate.

**Figure 3:**
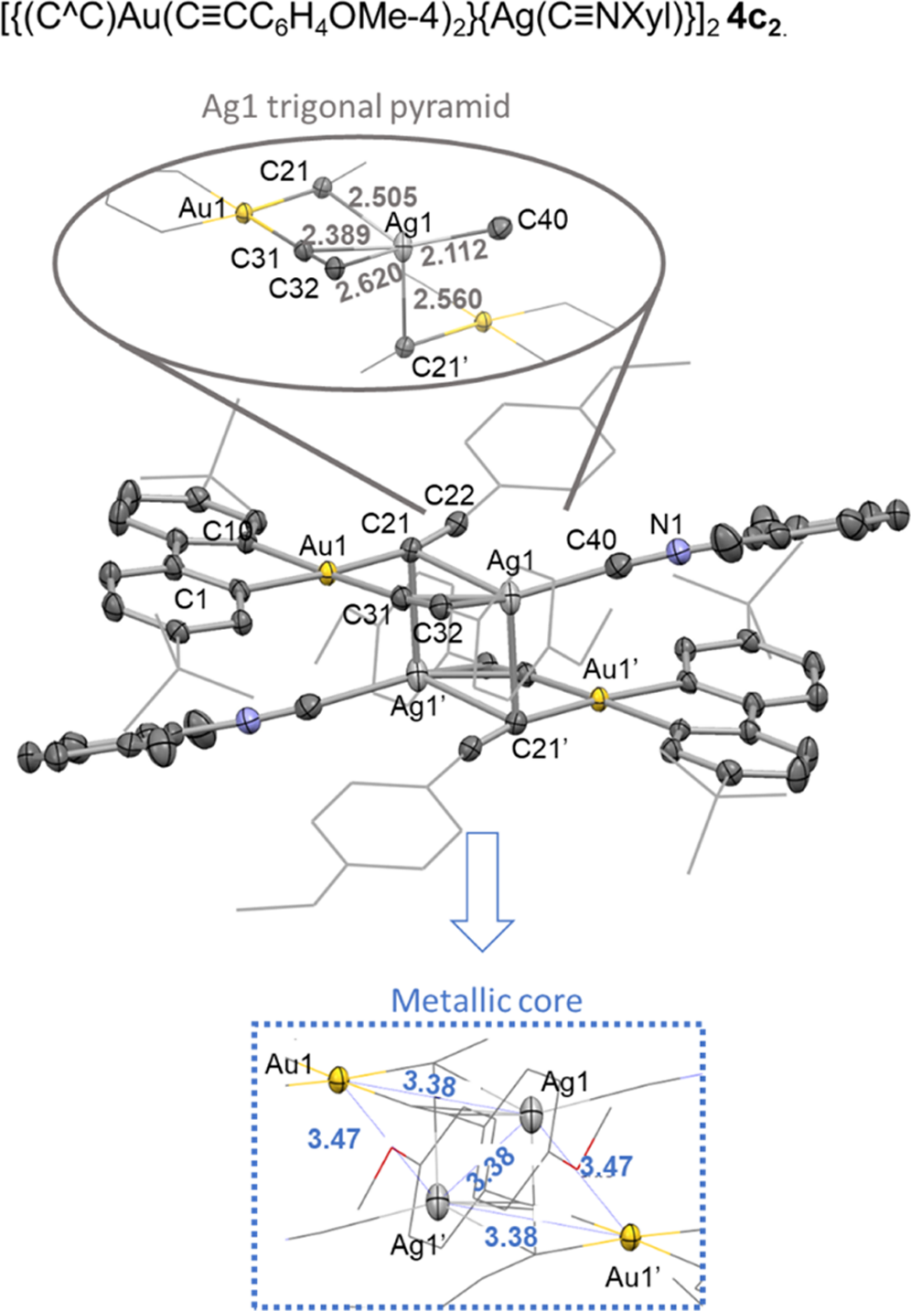
X-ray structure of [{(C^∧^C)Au(C≡CC_6_H_4_OMe-4)_2_}{Ag(C≡NXyl)}]_2_·CH_2_Cl_2_**4c**_**2**_·CH_2_Cl_2_**4c**·2 CH_2_Cl_2_. The structure is shown as basic skeleton with the most relevant atoms represented as ellipsoids with 50% probability level. Selected bond distances (Å) and angles (°): Au1–C1 2.046(3), Au1–C1 2.049(3), Au1–C21 2.072(4), C21–C22 1.210(5), Au1–C31 2.052(3), C31–C32 1.216(5), Ag1–C21 2.505(3), Ag1–C22 2.768(3), Ag1–C31 2.389(4), Ag1–C32 2.620(3), Ag1–C21′ 2.560(3), Ag1–C40 2.112(4), C40–N1 1.148(5), C1–Au1–C10 80.8(1), C1–Au1–C31 94.0(1), C10–Au1–C21 94.2(1), C21–Au1–C31 90.9(1), centroid(C31–C32)–Ag1–C21′ 108.1, centroid(C21–C22)–Ag1–C22 96.95, C21′–Ag1–C40 106.0(1), C21–Ag1–C31 73.8(1).

**Figure 4:**
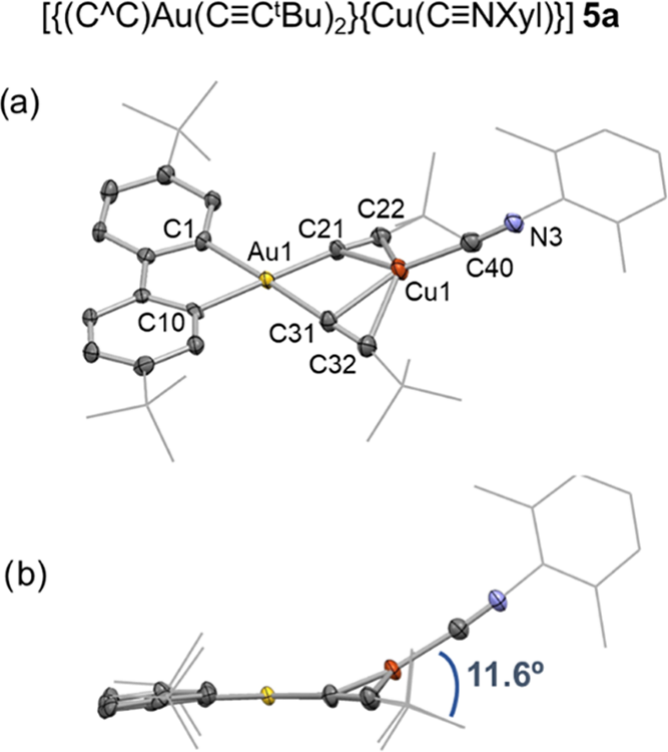
(a, b) X-ray structure of [{(C^∧^C)Au(C≡C*^t^*Bu)_2_}{Cu(C≡NXyl)}] **5a**. The structure is shown as basic skeleton with the most relevant atoms represented as ellipsoids with 50% probability level. Selected bond distances (Å) and angles (°): Au1–C1 2.031(4), Au1–C10 2.045(4), Au1–C21 2.047(4), C21–C22 1.209(6), Au1–C31 2.051(4), C31–C32 1.209(6), Cu1–C21 2.132(4), Cu1–C22 2.280(5), Cu1–C31 2.130(4), Cu1–C32 2.314(5), Cu1–C40 1.878(5), C40–N3 1.167(7), C1–Au1–C10 81.1(2), C1–Au1–C21 97.2(2), C10–Au1–C31 97.2(2), C21–Au1–C31 84.4(2), centroid(C21–C22)–Cu1-centroid(C31–C32) 110.29, centroid(C21–C22)–Cu1–C40 126.76, centroid(C31–C32)–Cu1–C40 122.94.

Crystals of **4c** were grown in CH_2_Cl_2_ and its X-ray structure is shown in Figure 4. The stoichiometry proposed in Scheme 2 was confirmed, but the structure reveals that the complex dimerizes as **4c**_**2**_ involving one of the alkynyl units of the closest molecule giving rise to a tetranuclear arrangement that clearly differs from that observed for **4a**. Thus, as the inset in Figure 3 shows, silver adopts a trigonal-pyramidal coordination environment with the equatorial positions occupied by the isocyanide carbon (Ag1–C40 2.112(4) Å), one alkyne bond η^2^-C≡C···Ag (Ag–C31 2.389(4), Ag1–C32 2.620(3) Å) and the C^α^ of the second alkynyl unit (Ag1–C21 2.505(3) Å) of the same gold fragment, as the Ag–C22 distance is too long to be considered a bond (Ag1–C22 2.768(3) Å). The Ag1 completes its requirements by bonding to the C^α^ of the alkynyl of the second gold fragment (Ag1–C21′ bond of 2.560(3) Å), which occupies the axial position of the trigonal pyramid. The good alignment between Ag1 with C21′ implies that both gold fragments sit in a shifted arrangement and are relatively close to each other, at 2.9 Å between both coordination planes (see Figure S14 for a top-down view). Nevertheless, the Au_2_Ag_2_ metallic core, represented in Figure 3, adopts a rhomboidal disposition forced by the symmetry, with long intermetallic distances (Au1···Ag1 3.38 Å, Au1···Ag1′ 3.47 Å, Ag1···Ag1′ 3.38 Å).

The structural disposition in **4c**_**2**_ is related to the supramolecular arrangement observed in **4a**. Thus, the Ag(I) ions in **4c**_**2**_ form a direct Ag–C bond (Ag1–C21′ 2.560 Å) with the second gold fragment, whereas this was exposed as a long-distance interaction in **4a** (Ag1···C106 3.270 Å, Ag2···C43 3.149 Å). This difference may be due to the higher electron density of the alkyl acetylide C≡C*^t^*Bu compared to C≡CC_6_H_4_OMe-4, which fulfills silver’s electronic requirements, as well as the steric hindrance of the *^t^*Bu residues of C≡C*^t^*Bu, which, in **4a**, precludes the structural disposition observed in **4c**_**2**_.

Crystals of [{(C^∧^C)Au(C≡C*^t^*Bu)_2_}{Cu(C≡NXyl)}] **5a** and [{(C^∧^C)Au(C≡CC_6_H_4_OMe-4)_2_}{Cu(C≡NXyl)}] **5c** were grown by layering a solution of the corresponding solid in CD_2_Cl_2_ with light petroleum. Unfortunately, the crystals of **5c** were not of sufficient quality to give a satisfactory model which might allow an analysis of distances and angles. However, a general view of the connectivity is included in Figure S15. As can be seen in Figure 4, in [{(C^∧^C)Au(C≡C*^t^*Bu)_2_}{Cu(C≡NXyl)}] **5a** the Cu(I) isocyanide fragment is disposed in a V-shape geometry, similar to **4a**, with an angle between the Au–Cu vector and the coordination plane of Au of 11.8°. While the quality of the crystal is poor, the connectivity in **5c** suggests an “in-plane” disposition for the Cu(I) fragment.

### Photophysical Properties

#### Absorption Spectra

**Figure 5:**
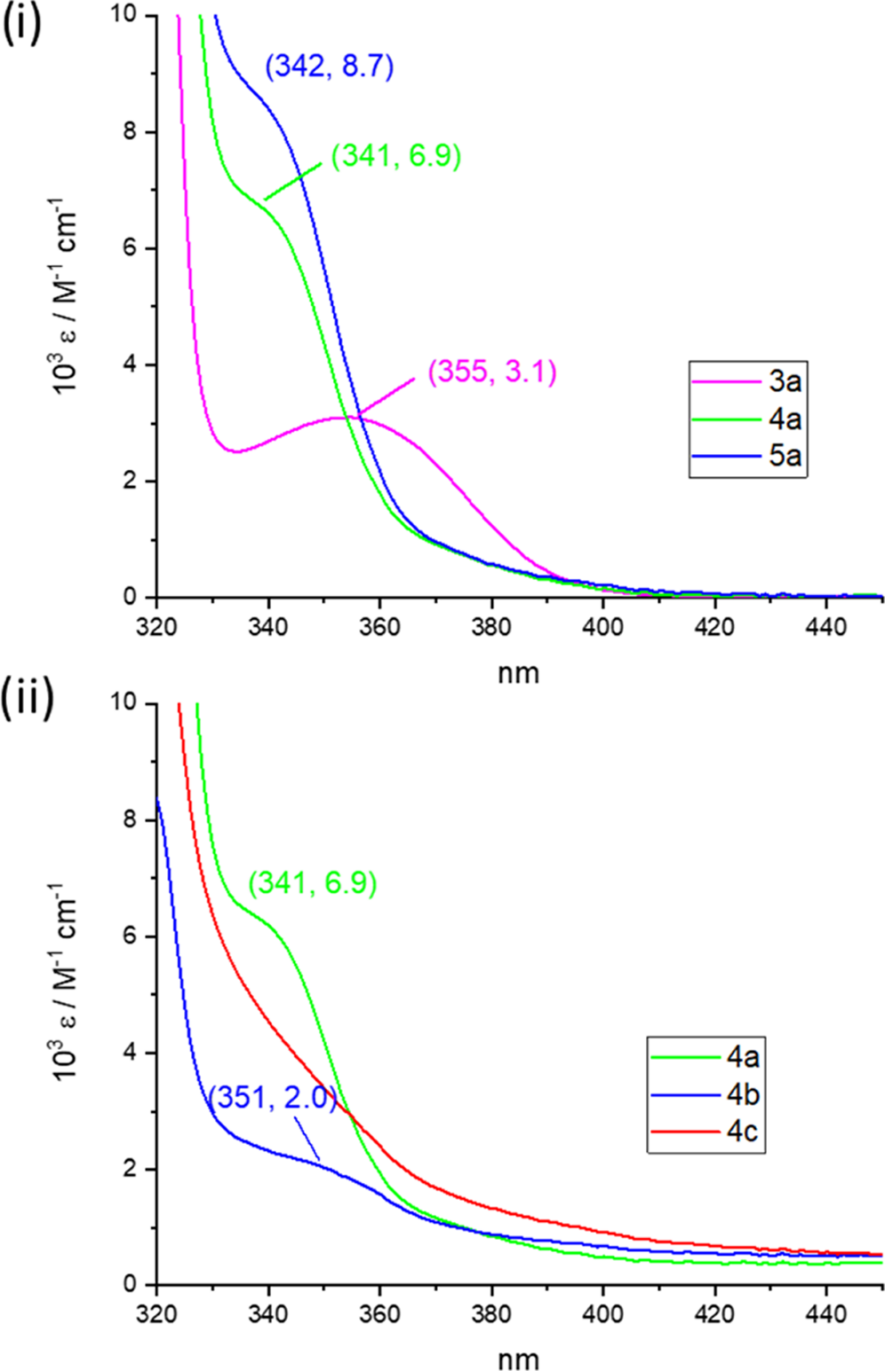
UV–vis absorption spectra of complexes (**3**–**5**)**a (i)** and **4(a**–**c) (ii)** 5 × 10^–5^ M in CH_2_Cl_2_.

The ultraviolet–visible (UV–vis) absorption spectra of the complexes were recorded in CH_2_Cl_2_. The data are summarized in Table S2. As an illustration, some selected examples are collected in Figures 5 and S16. The absorption bands observed in the UV region in all of the complexes can be attributed to ^1^IL transitions, which is consistent with the theoretical calculations. Among these transitions, the most distinct band appears at lower energies (340–370 nm), which still exhibits a strong ^1^IL(C^∧^C) character. However, based on the theoretical calculations, some degree of charge transfer toward Ag(**1**), Cu(**2**), and C≡NXyl (**3–5**) is also suggested.

Figure S16i shows that the low-energy absorption band is little impacted by the substitution of Ag/Cu in the tetranuclear aggregates [{(C^∧^C)Au(C≡C*^t^*Bu)_2_}_2_Ag_2_] **1** (355 nm) and [{(C^∧^C)Au(C≡C*^t^*Bu)_2_}_2_Cu_2_] **2** (354 nm), both of which have similar structures and contain the same type of gold fragments. However, while the mononuclear complex (C^∧^C)Au(C≡C*^t^*Bu)(C≡NXyl) **3a**, shows no shift of the low-energy band (355 nm), this is clearly blue-shifted for the binuclear derivatives [{(C^∧^C)Au(C≡C*^t^*Bu)_2_}{Ag(C≡NXyl)}] **4a** (341 nm) and [{(C^∧^C)Au(C≡C*^t^*Bu)_2_}{Cu(C≡NXyl)}] **5a** (342 nm) (Figure 5i). This shift is attributed to the involvement of the orbitals of the alkynyls and the gold center in the empty orbitals associated with the transition responsible for the low-energy absorption in these complexes. The influence of the alkynyl substituents in each of series also supports this conclusion. As shown in Table 1 and illustrated in Figure 5(ii) (see also Figure S16 and Table S2), the low-energy band follows the energy trend C≡C*^t^*Bu **a** > C≡CC_6_H_4_*^t^*Bu-4 **b** ≥ C≡CC_6_H_4_OMe-4 **c**. This bathochromic shift is consistent with the change from alkyl to aryl acetylide and the introduction of the -OMe substituent, which reduces the highest occupied molecular orbital-lowest unoccupied molecular orbital (HOMO–LUMO) gap of benzene rings by a resonant increase of the conjugation.

**Table 1:** Emission Properties of the Complexes

		λ_em_/nm (λ_ex_/nm)	τ/μs^a^ (λ_em_/nm)	ϕ^d^	*k*_r_ (s^–1^)^e^	*k*_nr_ (s^–1^)^f^
**1**	solid	496, 532_max_, 569, 614 (330–390)	47.05 (532)	0.10	2.13E^3^	1.91E^4^
	PMMA^b^	505, 535_max_, 581sh (330–390)	33.34 (535)	0.33	9.90E^3^	2.01E^4^
	CH_2_Cl_2_^c^	410br, 511, 542_max_, 578sh (320–360)	12.43(96%), 1.44(4%) (542)			
**2**	solid	494, 531_max_, 568, 611 (330–390)	50.07 (531)	0.07	1.40E^3^	1.86E^4^
	PMMA^b^	500_max_, 532, 574sh (320–370)	31.40 (532)	0.20	6.37E^3^	2.55E^4^
	CH_2_Cl_2_^c^	400, 424, 502, 537_max_, 574sh (320–360)	8.82 (537)			
**3a**	solid	490, 527_max_, 565, 604sh (320–380)	53.85 (527)	0.06	1.11E^3^	1.75E^4^
	PMMA^b^	496_max_, 528, 556sh (300–390)	38.94 (528)	0.22	5.65E^3^	2.00E^4^
	CH_2_Cl_2_^c^	410br, 501, 536_max_, 570sh (320–360)	8.65 (536)			
**3b**	solid	490, 527_max_, 560, 611sh (330–390)	39.67 (527)	0.12	3.02E^3^	2.22E^4^
	PMMA^b^	496_max_, 529, 556sh (300–390)	15.05 (529)	0.15	9.97E^3^	5.65E^4^
	CH_2_Cl_2_^c^	504, 539_max_, 572sh (320–360)	9.55 (71%), 1.05 (29%) (539)			
**3c**	solid	496, 533_max_, 565, 612sh (330–390)	32.90 (533)	0.11	3.34E^3^	2.71E^4^
	PMMA^b^	500, 532_max_, 568sh (300–390)	14.53 (532)	0.17	1.17E^4^	5.71E^4^
	CH_2_Cl_2_^c^	504, 538_max_, 572sh (320–360)	9.39 (76%), 1.11 (24%) (538)			
**4a**	solid	491, 527_max_, 563, 609sh (330–390)	50.89 (527)	0.08	1.57E^3^	1.81E^4^
	PMMA^b^	499, 531_max_, 564sh (300–400)	28.76 (531)	0.21	7.30E^3^	2.75E^4^
	CH_2_Cl_2_^c^	410br, 501, 537_max_, 570sh (320–360)	9.02 (74%), 1.05 (26%) (537)			
**4b**	solid	495, 532_max_, 567, 616sh (330–390)	47.80 (532)	0.18	3.77E^3^	1.72E^4^
	PMMA^b^	496_max_, 532, 564sh (300–400)	51.20 (532)	0.41	8.01E^3^	1.15E^4^
	CH_2_Cl_2_^c^	492, 528_max_, 562sh (320–360)	10.00 (77%), 2.09 (23%) (528)			
**4c**	solid	489, 527_max_, 567, 604sh (330–390)	44.31 (527)	≤0.01		
	PMMA^b^	493, 527_max_, 555sh (300–400)	41.33 (527)	0.07	1.69E^3^	2.25E^4^
	CH_2_Cl_2_^c^	496, 536, 562 (320–360)	9.50 (75%), 1.22 (25%) (536)			
**5a**	solid	440br, 504, 535_max_, 571sh (330–390)	24.32 (91%), 6.8 (9%) (535)	≤0.01		
	PMMA^b^	486, 516, 547sh (300–360)	27.04	0.06	2.22E^3^	3.48E^4^
	CH_2_Cl_2_^c^	425_max_, 497, 530, 597sh (320–360)	9.52 (78%), 1.16 (22%) (530)			
**5b**	solid	492, 527_max_, 565, 608sh (330–390)	21(69%), 56(31%) (527)	0.07	2.20E^3^^g^	2.92E^4^^g^
	PMMA^b^	488, 521, 551sh (300–360)	32.83 (84%), 10.00 (16%) (521)	0.20	6.85E^3^^g^	2.74E^4^^g^
	CH_2_Cl_2_^c^	448, 489, 526, 561 (320–360)	8.79 (78%), 1.06 (22%) (526)			
**5c**	solid	448br, 489, 526_max_, 569, 607sh (330–390)	7.4 (39%), 22 (61%) (526)	0.08	4.90E^3^^g^	5.64E^4^^g^
	PMMA^b^	489, 521, 551sh (300–360)	1.46 (4%), 26.44 (96%) (521)	0.21	8.25E^3^^g^	3.10E^4^^g^
	CH_2_Cl_2_^c^	433, 487, 523, 614sh (320–360)	9.26 (86%), 1.34 (14%) (523)			

a Excitation at 350–360 nm with μF2 pulse lamp.

b PMMA film (10 wt %).

c Deoxygenated CH_2_Cl_2_ solution (∼1E^–3^ M).

d λ_ex_ 360 nm.

e *k*_r_ = ϕ/τ.

f *k*_nr_ = (1 – ϕ)/τ.

g Using τ_av_ Calc. emissions (nm): 549 (**1**), 552 (**2**), 542 (**3a**), 541 (**4c**), 536 (**5a**), 538 (**5c**).

#### Photoluminescence Spectra

**Figure 6:**
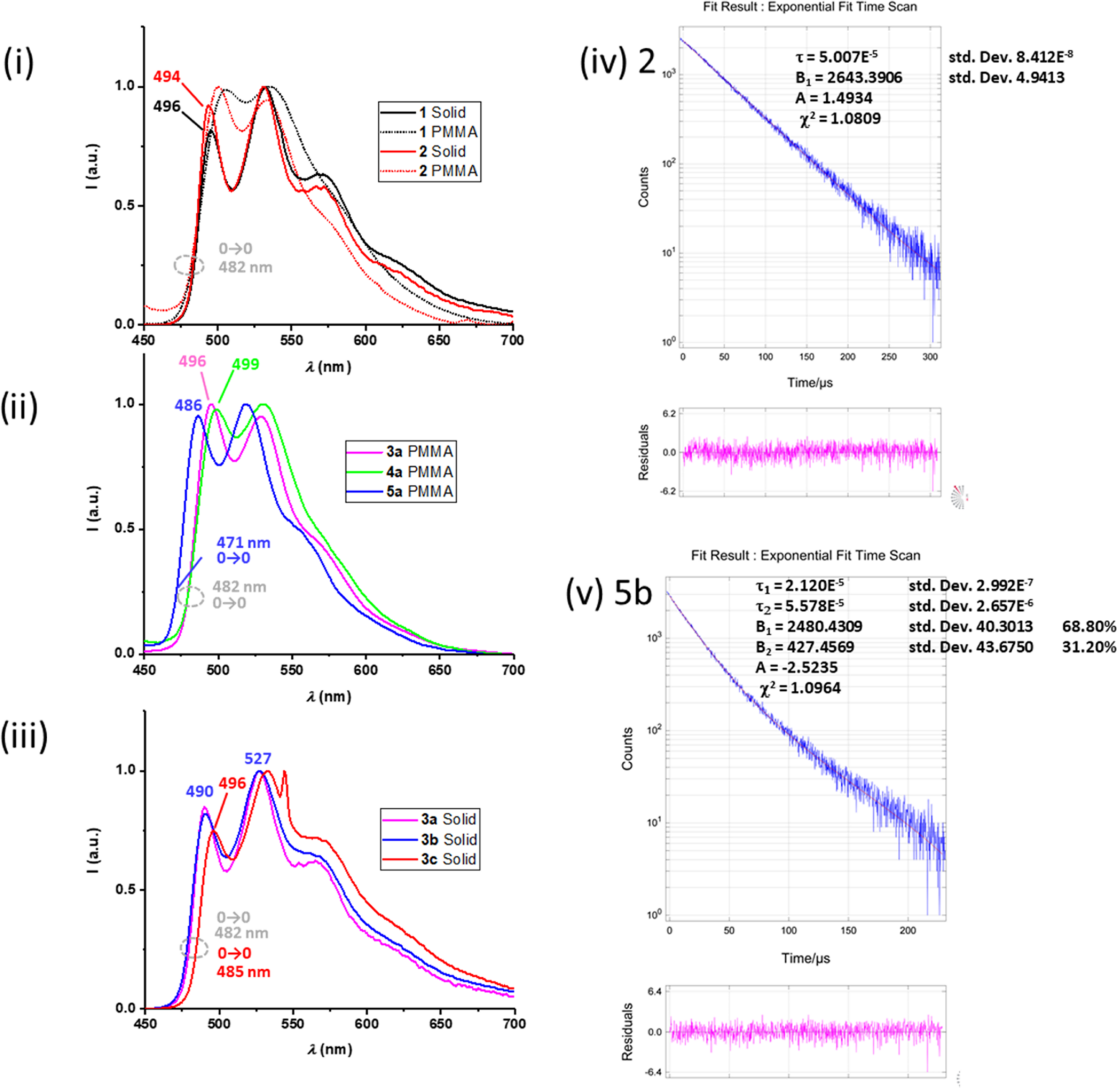
PL spectra of complexes **1** and **2** in PMMA film (10 wt %) (i), **3**–**5a** in PMMA film (10 wt %) (ii), and **3**(**a**–**c**) (iii) in the solid state. Decay curve of the emission at 531 nm for complex **2** in the solid state (iv). Decay curve of the emission at 527 nm for complex **5b** in the solid state (v).

The photoluminescence properties of the complexes in the solid state (microcrystalline powders), in PMMA films (prepared by drop casting mixtures of the complex and poly(methyl methacrylate) (PMMA) solution (10 wt %)) and in deoxygenated CH_2_Cl_2_ solution (∼1 × 10^–3^ M) are summarized in Table 1 and selected spectra are provided in Figure 6.

All complexes exhibit similar vibronically structured emissions in the solid state and in PMMA films, with slight red shifts in PMMA and long lifetimes in the range of 20–50 μs. Based on the Stokes shift and the lifetimes in the microsecond range, a phosphorescent emission behavior is proposed, which is consistent with the assignment proposed by other researchers for systems with the same dominating “(C^∧^C)Au” chromophore,^29,32^ and supported by theoretical calculations. Comparison between solid state and PMMA photophysical data reveals a noticeable increase in the quantum yields in PMMA films that in most cases is accompanied by a reduction in the lifetime of the emission. This fact could be attributed to the occurrence of facile triplet–triplet annihilation in the solid state for which closer intermolecular interactions are allowed.^48^ In fact, the analysis of the radiative *k*_r_ and nonradiative *k*_nr_ constants shows an increase of both in PMMA with respect to the solid state but the radiative constant is increased to a larger extent, which explains the higher QY in PMMA. We note that the Cu isocyanide series **5a-c** shows biexponential decays (see Figure 6(v) for **5b**), suggesting the contribution of two close emissive states that could be related to the presence of V-shape and in-plane coordination geometries of the bis(alkynyl)CuCNR units.

As can be seen in Figure 6(ii) for PMMA, the emission energy of the higher-energy maximum of the Cu(I) dinuclear complex [{(C^∧^C)Au (C≡C*^t^*Bu)_2_}{Cu(C≡NXyl)}] **5a** (486 nm) is blue-shifted with respect to the other C≡C*^t^*Bu containing complexes. For the rest of the *^t^*Bu **a** series, the first maxima appear in the energy sequence 496 (**3a)** > 499 (**4a)** > 500 (**2)** > 505 nm (**1**). However, a simple analysis of the position of the emission maxima usually leads to misunderstanding when translated into conclusions about the electronic transitions without considering the vibrational overlap. This is particularly risky in the solid state or in films, like in this case. In fact, the 0 → 0 transition, taken at the origin of the emission band, for **1**, **2**, **3a**, and **4a** is around 482 nm but is clearly blue-shifted to around 471 nm for **5a**. The position of the 0 → 0 transition is the same in the solid state and in PMMA for each complex. This means that the different positions for the emission maxima are most likely related to the different vibrational overlap between the excited and the ground states in the radiative process. This is in fact consistent with the vibrational spacings of the emission bands that fit well in the C=C and C–C stretching region.

In each series of complexes, **3**, **4**, and **5**, the modification of the substituent on the alkynyl ligand has little effect on the energy of the emission. As can be seen in Figure 6(iii) for the solid state, only in the comparison of **3a,b** vs **3c** a slight red shift of the emission maxima (490 **3a,b***vs* 496 nm **3c**) is observed by the introduction of the C_6_H_4_OMe-4, thus mimicking the variation found in the absorption maxima. This shift is also reflected in the 0 → 0 transition (482 nm **3a,b** vs 485 nm **3c**) and its small magnitude indicates the dominating role of the “(C^∧^C)Au” chromophore in the emissive state, also supported by theoretical calculations.

**Figure 7:**
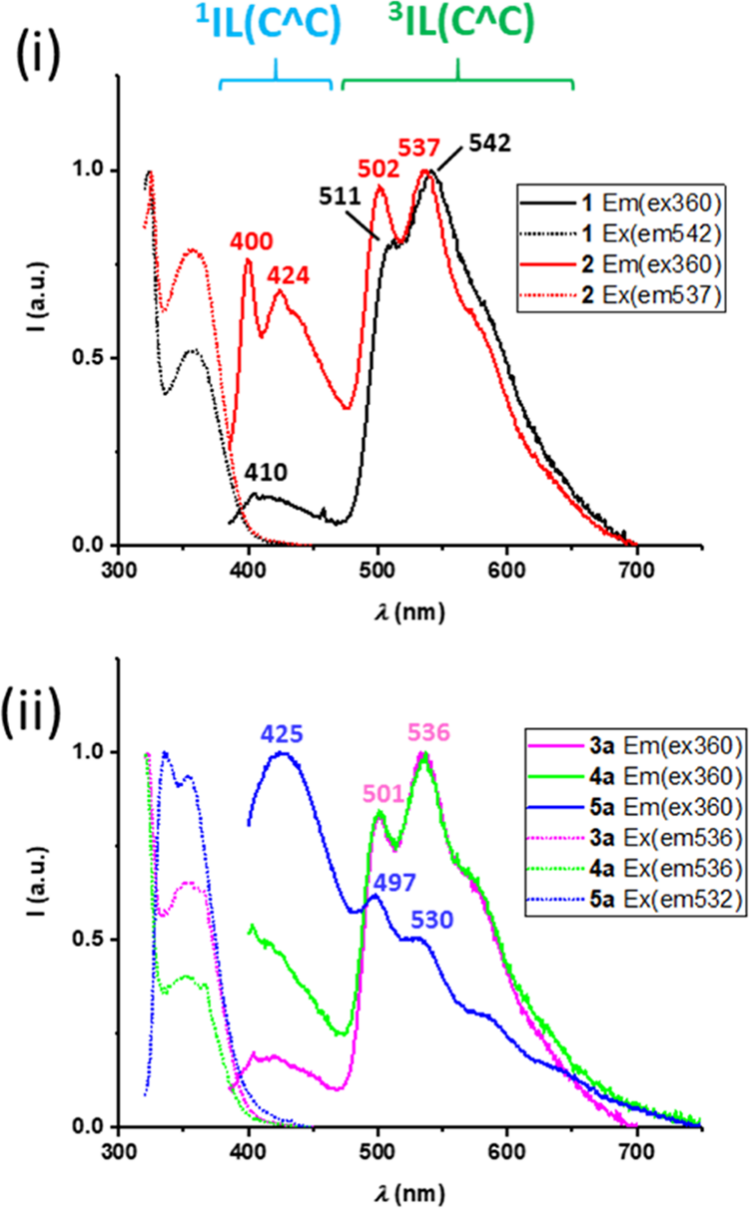
PL spectra (emission as solid lines and excitation as dotted lines) of complexes **1** and **2** (i) and **3**–**5a** (ii) in CH_2_Cl_2_ (∼1 × 10^–3^ M).

Despite a relatively high intensity in the solid state and in PMMA, the emission in solution is weak, most likely due to deactivation through vibrational relaxation to nonradiative d-d states, which is a common phenomenon in luminescent (C^∧^C)Au(III) complexes.^29,49^ However, under strict deoxygenated conditions, the blue-greenish emission of the complexes in CH_2_Cl_2_ can be monitored. The data are collected in Table 1, and selected examples are shown in Figures 7 and S18.

In all cases, in fluid CH_2_Cl_2_ solution, the complexes exhibit a dual emission profile formed by a high-energy band in the blue region (400–460 nm) and a low-energy structured band in the green region (490–700 nm). The low-energy band appears in a similar region to the one observed in the solid state and PMMA and is strongly quenched by oxygen; therefore, it is assigned to intraligand ^3^IL(C^∧^C) phosphorescence. The high-energy band has a similar excitation profile to the low-energy band and is not affected by oxygen, so is ascribed to ^1^IL(C^∧^C) fluorescence. This suggests that intersystem crossing is not very effective in these complexes at 298 K, particularly for tetra- and bimetallic compounds for which the contribution of the high-energy band is dominant.

### Theoretical Calculations

To better understand the photophysical properties of the complexes, density functional theory (DFT) and time-dependent density functional theory (TD-DFT) calculations were performed for **1**, **2**, **3a**, **4c**, **5a**, and **5c**. Calculations of the ground state and the absorption spectra were carried out in dichloromethane using the polarizable continuum model (PCM), which allows comparison with the experimental data. The photophysical properties of the complexes were found to be dominated by the C^∧^C fragment in the orbitals involved in the transitions responsible for light absorption and emission. This result was consistent across all of the complexes studied.

#### Geometry

From a structural perspective (refer to Table S3), the most notable characteristic of the tetranuclear aggregates **1** and **2** is that the computed ground and triplet state optimizations of the clusters display shorter distances between the coordination planes of the Au(III) units than their X-ray structures (experimental values ranging from 5.52 to 5.47 Å, while computed values range from 5.13 to 4.90 Å). The structure of **1** at S^0^ level shows slight elongation of the shorter Au–Ag distances and shortening of the longer Au–Ag distances, this results in more similar intermetallic distances with respect to those obtained from the X-ray structure. Nevertheless, the calculated distances (3.237–3.250 Å) are clearly shorter than the sum of the van der Waals radii of both atoms (3.38 Å) reinforcing the idea of attractive Au(III)–Ag(I) interactions.

On the other hand, the optimized geometries of compounds **4c**, **5a**, and **5c** indicate that the M–C≡NXyl moiety is arranged “in plane” relative to the coordination plane of the Au atom. This finding is consistent with the notion that the “V-shape” disposition is linked to the accommodation of supramolecular interactions in the crystal lattice, while the “in-plane” arrangement is adopted in the absence of these forces, as previously discussed in the Synthesis and Characterization Section.

#### Absorption

**Figure 8:**
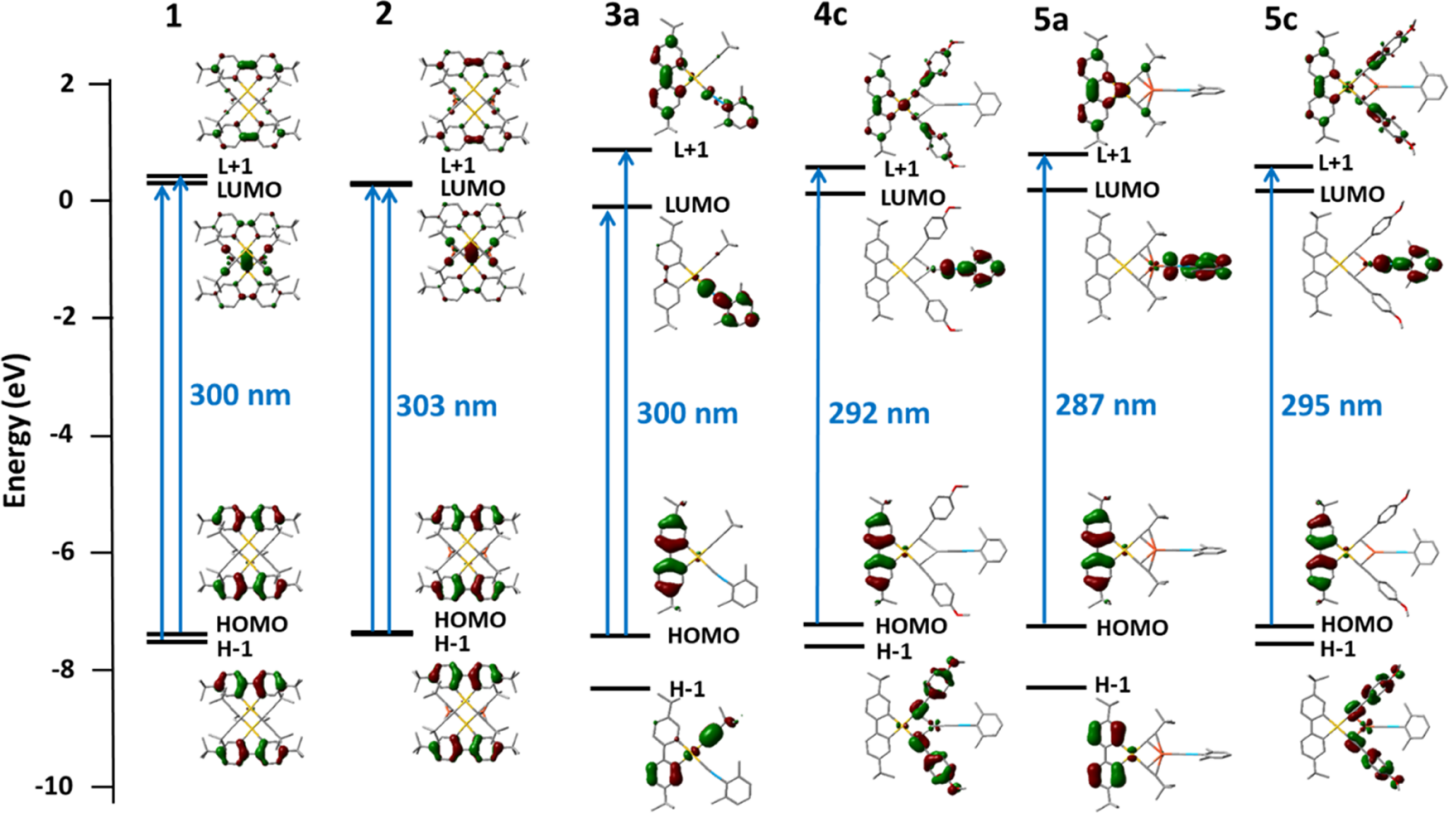
Schematic representations of the HOMO – 1, HOMO, LUMO, and LUMO + 1 and calculated lower-energy transitions for **1**, **2**, **3a**, **4c**, **5a**, and **5c**.

Table S4 illustrates the composition of the frontier molecular orbitals for the complexes chosen in the calculations, while Figures S19–S24 display the shape of these orbitals (from HOMO – 3 to LUMO + 3) and Figure 8 extracts the orbitals that determine the nature of the lower-energy absorption (HOMO – 1, HOMO, LUMO and LUMO + 1). At the optimized ground state geometry, the HOMO is situated on the C^∧^C ligand for all of the complexes, with negligible participation of the gold center or the rest of the molecule (1-2%). However, to gain a better understanding of the transition that leads to lower-energy absorption, relying solely on the analysis of the LUMO could lead to misunderstandings. As shown in Table S5, the lowest-energy transition (with *f* values greater than 0.1) does not correspond to the HOMO → LUMO transition. For complexes **1** and **2**, this transition is a mixture of HOMO – 1 → LUMO and HOMO → LUMO + 1, indicating some charge transfer character toward Ag and Cu. In the same line, for complexes **3a**, **4c**, **5a**, and **5c**, the lower-energy transition is an admixture ^1^IL/^1^LLCT (C^∧^C → C^∧^C/C≡NXyl). The contribution of the isocyanide in the transition comes from the LUMO, in all cases strongly polarized toward this ligand. For **4c** and **5c**, some contribution of the C≡CC_6_H_4_OMe-4 fragments that arise from the LUMO + 1 orbital is also noted.

The calculated energies are consistent with the experimental data. Specifically, for complexes **1**, **2**, and **3a**, the calculated energies are nearly identical (around 300 nm), with a slightly lower energy for complex **2** (303 nm). The complexes in the **4** and **5** series exhibit transitions at higher energies. Notably, complex **5a** displays a blue-shifted calculated transition (287 nm), having the same alkynyl substituent as complexes **1**, **2**, and **3a**, the same effect that was observed experimentally. The calculated transitions also predict the red shift observed upon the introduction of the C_6_H_4_OMe-4 substituent, as evidenced by the comparison of **5a** (287 nm) with **5c** (295 nm).

For a better understanding of the emissive properties, the T_1_ excited state optimizations and the emission properties were calculated without any solvent. As detailed in the SI, Section S4, from the three functionals evaluated, named B3LYP3, CAM-B3LYP4, and ωB97X-D5,6, the dispersion-corrected hybrid density functional ωB97X-D predicted the emission energies best. The basis set used for the metal atoms was the LanL2DZ effective core potential and 6-31G(d,p) for the ligand atoms. The emission energies were calculated as the difference between the optimized T_1_ state and the S_0_ state in the optimized T_1_ geometry (adiabatic electronic transition). These calculated emission wavelengths are plotted and compared with the experimental value in Table 1. Despite the general red shift for the calculated emissions compared with the corresponding experimental, the general trend is supported. It is particularly relevant that our calculations predict the small blue shift of the Cu bimetallic type of complex **5** with respect to the others, as can be seen comparing, for example, in PMMA, **5a** (536 nm) *vs***3a** (542 nm) and **2** (552 nm) or **5c** (538 nm) vs **4c** (541 nm) and **2** (552 nm).

**Figure 9:**
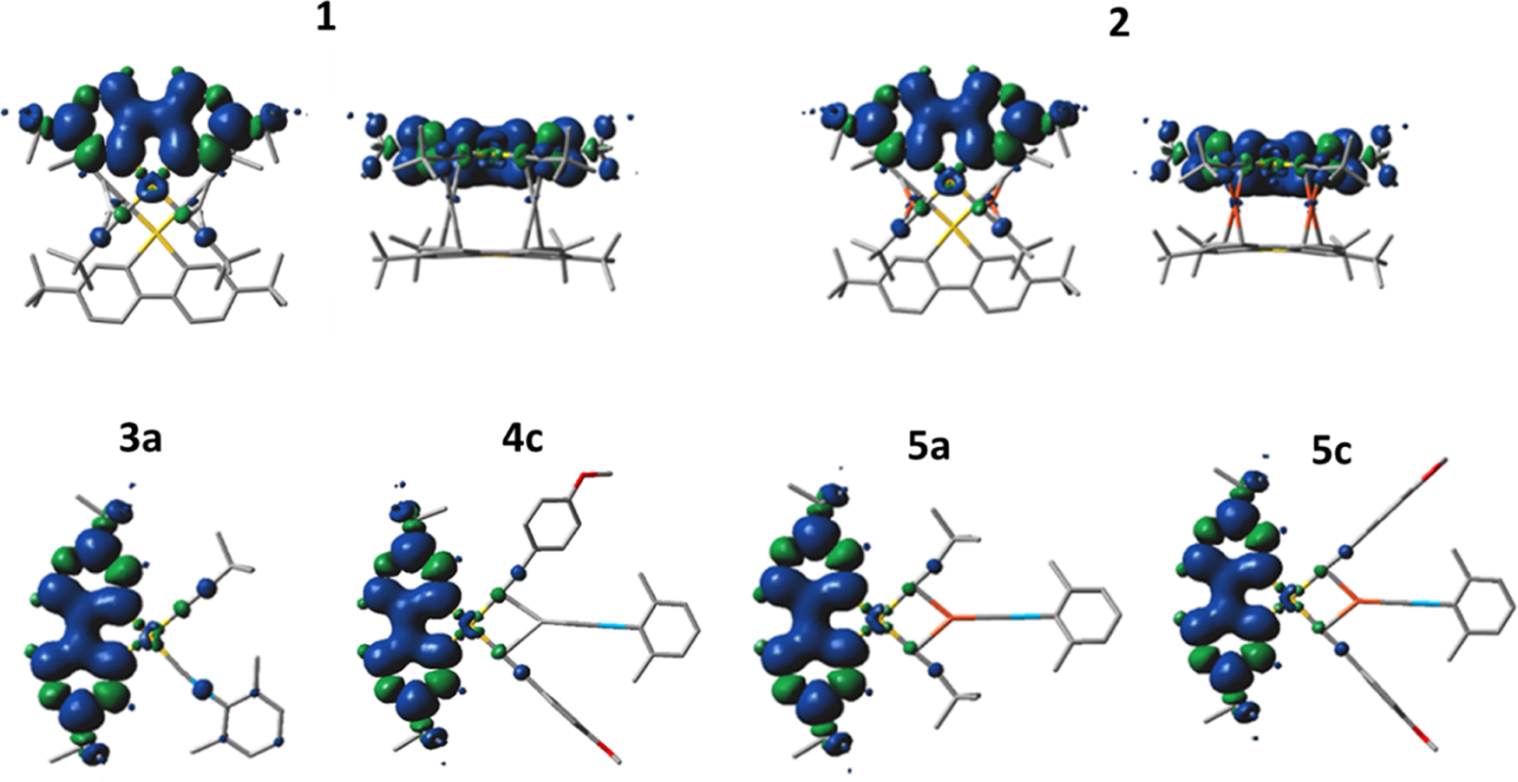
Spin distribution for the lowest triplet excited states in **1**, **2**, **3a**, **4c**, **5a**, and **5c**.

The distribution of the singly occupied molecular orbital (SOMO) and SOMO – 1 orbital and the spin distribution for the lowest triplet excited state support the conclusion that the emission in all of these complexes has ^3^IL(C^∧^C) character, with a minor contribution of the Au atom (∼5%) in the SOMO orbital. This result is consistent with the limited modulation of the PL observed, despite the very different structures, and with the long monitored excited state lifetimes. Accordingly, the slightly different emission energies observed for these systems might be attributed to the indirect effect of the alkynyl ligands and the heterometal bonded to the gold center on the relative energies of the C^∧^C frontier orbitals of this chromophore (Figure 9).

## Conclusions

In summary, we report the synthesis, characterization, X-ray structures, and photophysical properties of C^∧^C-chelated Au(III) complexes with alkynyl and isocyanide ligands and Ag(I) or Cu(I) ions. The products were obtained by exploring the reactivity of [(C^∧^C)AuCl]_2_ and (C^∧^C)AuCl(CNXyl) toward AgC≡CR (R = *^t^*Bu, C_6_H_4_*^t^*Bu-4, C_6_H_4_OMe-4). The tetranuclear aggregate [{(C^∧^C)Au(C≡C*^t^*Bu)_2_}_2_Ag_2_] **1**, the mononuclear (C^∧^C)Au(C≡CR)(C≡NXyl) **3**, and binuclear [{(C^∧^C)Au(C≡CR)_2_}{Ag(C≡NXyl)}] **4** series (R = *^t^*Bu **a**, C_6_H_4_*^t^*Bu-4 **b**, C_6_H_4_OMe-4 **c**) are accessible this way. Besides, using the insolubility of AgCl as a driving force, metal exchange with CuCl gives access to the complexes; [{(C^∧^C)Au(C≡C*^t^*Bu)_2_}_2_Cu_2_] **2**, [{(C^∧^C)Au(C≡CR)_2_}{Cu(C≡NXyl)}] (R = *^t^*Bu **5a**, C_6_H_4_*^t^*Bu-4 **5b**, C_6_H_4_OMe-4 **5c**).

This family of complexes exhibits a wide range of structural features that are directed by the various coordination modes of the Ag(I) and Cu(I) centers. These coordination modes range from linear to distorted four-coordinate, passing through trigonal planar geometries, and they are stabilized by M···(C≡C) bonds and the isocyanide. The Au fragments described in this paper feature a unique “all-carbon” anionic coordination environment.

Due to the high-field ligands in the coordination environment of Au(III), all of the complexes exhibit intense phosphorescence in the solid state and in a PMMA matrix. However, in solution, the complexes show weak dual emissions, ascribed to fluorescence and phosphorescence. Surprisingly, despite the structural richness, the photoluminescence is dominated by ^3^IL(C^∧^C) transitions, which limits access to a broad range of emission energies. These results open the door for the synthesis of more elaborate complexes using similar strategies.

## Experimental Section

### General Considerations

When required, manipulations were performed by using standard Schlenk techniques under dry N_2_. All solvents were dried by means of the appropriate drying agent and distilled. Dichloromethane–*d*_2_ was stored in the glovebox over activated 4 Å molecular sieves. [(C^∧^C)AuCl]_2_ (**A**), and (C^∧^C)AuCl(CNXyl) (**B**) were synthesized according to literature procedures.^28^ Elemental analyses were performed by London Metropolitan University. Infrared spectra were recorded using a PerkinElmer Spectrum 65 FT-IR spectrometer with a diamond ATR attachment. Matrix-assisted laser desorption/ionization-time of flight (MALDI-TOF) spectra were collected in a Microflex MALDI-TOF Bruker spectrometer in the negative ion mode in CH_2_Cl_2_. ^1^H and ^13^C{^1^H} NMR experiments were recorded using a Bruker DPX–300 spectrometer equipped with a ^1^H,BB smartprobe and Z-gradients. ^1^H NMR spectra are referenced to the residual protons of the deuterated solvent. ^13^C NMR spectra are referenced to the D-coupled ^13^C signals of the solvent. Numbering schemes can be found in Schemes 1 and 2.

#### Synthesis of [{(C^∧^C)Au(C≡C*^t^*Bu)_2_}_2_Ag_2_] **1**

Ag–C≡C*^t^*Bu (0.114 g, 0.604 mmol) was added to a suspension of [(C^∧^C)AuCl]_2_ (0.100 g, 0.098 mmol) in 40 mL of dichloromethane. The mixture was stirred for 20 h protected from the light and then filtered through a celite pad. The solution was evaporated to a small volume, and petrol is slowly added to obtain the product as pale yellow crystals (0.122 g, 0.083 mmol, 83% yield). Anal. Calcd for C_64_H_84_Ag_2_Au_2_ (1463.1): C, 52.54; H, 5.79. Found: C, 52.25; H, 5.96. IR (cm^–1^): ν(C≡C) 2044 (m). MALDI-TOF (−): *m*/*z* (%) 823 [(C^∧^C)Au(C≡C*^t^*Bu)_2_] (10), 483 [(C^∧^C)AuNa] (20), 293 [AuO(C≡C*^t^*Bu)] (90). ^1^H NMR (CD_2_Cl_2_, 300.13 MHz, 298 K): δ 7.96 (d, ^4^*J*_HH_ = 1.8 Hz, 4H, H^2^), 7.26 (d, ^3^*J*_HH_ = 8.0 Hz, 4 H, H^5^), 7.15 (dd, ^3^*J*_HH_ = 8.0 Hz, ^4^*J*_HH_ = 1.8 Hz, 4 H, H^4^), 1.31 (s, 36 H, *^t^*Bu), 1.26 (s, 36 H, *^t^*Bu). ^13^C{^1^H} NMR (CD_2_Cl_2_, 75 MHz, 298 K): δ 155.0 (C^1^), 152.4 (C^3^), 149.0 (C^6^), 133.3 (C^2^), 128.1 (d, *J*_CAg_ = 7.1 Hz, C^β^), 123.8 (C^4^), 120.1 (C^5^), 95.0 (d, *J*_CAg_ = 9.0 Hz, C^α^), 34.8 (*C*CH_3,_*^t^*Bu), 32.4 (*C*H_3,_*^t^*Bu), 32.1 (*C*CH_3,_*^t^*Bu), 31.3 (*C*H_3,_*^t^*Bu).

#### Synthesis of [{(C^∧^C)Au(C≡C*^t^*Bu)_2_}_2_Cu_2_] **2**

CuCl (0.017 g, 0.168 mmol) is added to a solution of [(C^∧^C)Au(C≡C*^t^*Bu)_2_]Ag_2_ (0.061 g, 0.042 mmol) in 20 mL of dichloromethane. The white suspension is stirred for 20 h protected from the light and then filtered over celite. The yellow solution is evaporated to dryness to obtain the product as a white solid (0.040 g, 0.029 mmol, 70% yield). Anal. Calcd for C_64_H_84_Cu_2_Au_2_ (1374.4): C, 55.93; H, 6.16. Found: C, 56.12; H, 6.20. IR (cm^–1^): ν(C≡C) Not observed. MALDI-TOF (−): *m*/*z* (%) No significant peak observed. ^1^H NMR (CD_2_Cl_2_, 300.13 MHz, 298 K): δ 7.93 (s, 4H, H^2^), 7.27 (d, ^3^*J*_HH_ = 8.0 Hz, 4 H, H^5^), 7.16 (d, ^3^*J*_HH_ = 8.0 Hz, 4 H, H^4^), 1.34 (s, 36 H, *^t^*Bu), 1.32 (s, 36 H, *^t^*Bu). ^13^C{^1^H} NMR (CD_2_Cl_2_, 75 MHz, 298 K): δ 155.2 (C^1^), 152.4 (C^3^), 149.0 (C^6^), 133.4 (C^2^), 129.9 (C^β^), 123.8 (C^4^), 120.1 (C^5^), 108.3 (C^α^), 34.8 (*C*CH_3,_*^t^*Bu), 32.1 (*C*H_3,_*^t^*Bu), 31.6 (*C*CH_3,_*^t^*Bu), 31.3 (*C*H_3,_*^t^*Bu).

#### Synthesis of (C^∧^C)Au(C≡C*^t^*Bu)(C≡NXyl) **3a**

A solution of (C^∧^C)AuCl(C≡NXyl) (80 mg, 0.13 mmol) in CH_2_Cl_2_ was treated with AgC≡C*^t^*Bu (25 mg, 0.13 mmol). The mixture was stirred at room temperature for 24 h in a dark flask and then filtered through a celite pad. The volatiles were evaporated and the whitish residue was washed with diethyl ether (2 × 20 mL). The product was isolated as a white powder. (80 mg, 91%). Anal. Calcd for C_35_H_42_AuN (673.7): C, 62.40; H, 6.28; N, 2.08. Found: C, 62.25; H, 6.35; N, 2.02. IR (cm^–1^): ν(C≡N) 2212 (m). ν(C≡C) Not observed. MALDI-TOF (−): *m*/*z* (%) 542 [(C^∧^C)Au(C≡C*^t^*Bu)] (100), 647 [(C^∧^C)Au(C≡C*^t^*Bu)_2_Na]. ^1^H NMR (CD_2_Cl_2_, 300.13 MHz, 298 K): δ 8.40 (d, ^4^*J*_HH_ = 1.8 Hz, 1H, H^2^), 7.85 (d, ^4^*J*_HH_ = 1.8 Hz, 1H, H^2′^), 7.41 (m, 1H, H^10^), 7.33 (m, 1H, H^5′^), 7.27 (m, 3H, H^9^, H^4′^), 7.20 (m, 2H, H^4^, H^5^), 2.68 (s, 6H, CH_3,_Xyl), 1.35 (s, 9H, *^t^*Bu), 1.34 (s, 9H, *^t^*Bu), 1.32 (s, 9 H, *^t^*Bu). ^13^C{^1^H} NMR (CD_2_Cl_2_, 75 MHz, 298 K): δ 159.9 (C^1′^), 152.9 (C^1^), 151.6 (C^6^), 151.1 (C^6′^), 150.2 (C^3^), 149.3 (C^3′^), 136.8 (C^8^), 134.8 (C^2′^), 134.2 (C^2^), 131.3 (C^10^), 128.4 (C^9^), 124.7 (C^7^), 124.3 (C^4^), 123.8 (C^5^), 120.4, 120.3 (C^4′^, C^5′^), 114.7 (C^β^, C≡C*^t^*Bu), 101.2 (C^α^, C≡C*^t^*Bu), 35.0, 34.7 (*C*CH_3,_*^t^*Bu, C^∧^C), 32.0 (*C*H_3,_*^t^*Bu, C≡C*^t^*Bu), 31.3 (*C*H_3,_*^t^*Bu, C^∧^C), 28.9 (*C*CH_3,_*^t^*Bu, C≡C*^t^*Bu), 19.1 (*C*H_3,_ Xyl).

#### Synthesis of (C^∧^C)Au(C≡CC_6_H_4_*^t^*Bu-4)(C≡NXyl) **3b**

The complex was prepared using the same synthetic procedure described for **3a** but starting from (C^∧^C)AuCl(C≡NXyl) (80 mg, 0.13 mmol) and AgC≡CC_6_H_4_*^t^*Bu (35 mg, 0.13 mmol). Isolated as a white powder. (90 mg, 92%). Anal. Calcd for C_41_H_46_AuN (749.8): C, 65.70; H, 6.18; N, 1.87. Found: C, 65.85; H, 6.55; N, 2.00. IR (cm^–1^): ν(C≡N) 2214 (m). ν(C≡C) Not observed. MALDI-TOF (−): m/z (%) 618 [(C^∧^C)Au(C≡CC_6_H_4_*^t^*Bu-4)] (100), 775 [(C^∧^C)Au(C≡C_6_H_4_*^t^*Bu-4)_2_] (5). ^1^H NMR (CD_2_Cl_2_, 300.13 MHz, 298 K): δ 8.51 (d, ^4^*J*_HH_ = 1.9 Hz, 1H, H^2′^), 7.88 (d, ^4^*J*_HH_ = 1.9 Hz, 1H, H^2^), 7.45–7.20 (m, 12 H, aromatic), 2.69 (s, 6H, CH_3,_ Xyl), 1.36 (s, 9H, *^t^*Bu), 1.34 (s, 9H, *^t^*Bu), 1.33 (s, 9H, *^t^*Bu). ^13^C{^1^H} NMR (CD_2_Cl_2_, 75 MHz, 298 K): δ 159.39 (C^1′^); 152.72, 151.52, 151.32 (C^1^, C^6^, C^6′^), 150.35, 149.85 (C^3^, C^3′^), 149.55 (C^14^), 136.79 (C^8^), 134.80 (C^2′^), 134.08 (C^2^), 131.9 (C^13^), 131.39 (C^10^), 131.04 (C^11^), 128.48 (C^9^), 125.20 (C^7^), 125.3 (C^β^, C≡CC_6_H_4_*^t^*Bu-4), 124.54, 123.97, 123.52, 120.57, 120.43 (C^4^, C^5^, C^4′^, C^5′,^ C^12^), 105.96 (C^α^, C≡CC_6_H_4_*^t^*Bu-4), 34.87 (*C*CH_3,_*^t^*Bu, C^∧^C), 34.69 (*C*CH_3,_*^t^*Bu, C^∧^C), 34.53 (*C*H_3,_*^t^*Bu, C_6_H_4_*^t^*Bu-4), 31.26 (*C*H_3,_*^t^*Bu, C^∧^C), 31.22 (*C*H_3,_*^t^*Bu, C^∧^C), 30.98 (*C*H_3,_*^t^*Bu, C^∧^C), 18.93 (*C*H_3,_ Xyl).

#### Synthesis of (C^∧^C)Au(C≡CC_6_H_4_OMe-4)(C≡NXyl) **3c**

The complex was prepared using the same synthetic procedure described for **3a** but starting from (C^∧^C)AuCl(C≡NXyl) (80 mg, 0.13 mmol) and AgC≡CC_6_H_4_OMe-4 (30 mg, 0.13 mmol). Isolated as a white powder. (82 mg, 87%). Anal. Calcd for C_38_H_40_AuNO (723.7): C, 63.07; H, 5.57; N, 1.94. Found: C, 62.75; H, 5.90; N, 2.04. IR (cm^–1^): ν(C≡N) 2217 (m). ν(C≡C) Not observed. MALDI-TOF (−): *m*/*z* (%) 592 [(C^∧^C)Au(C≡CC_6_H_4_OMe-4)] (100), 725 [(C^∧^C)Au(C≡C_6_H_4_*^t^*Bu-4)(C≡NXyl)] (5). ^1^H NMR (CD_2_Cl_2_, 300.13 MHz, 298 K): δ 8.50 (d, ^4^*J*_HH_ = 1.8 Hz, 1H, H^2′^), 7.88 (d, ^4^*J*_HH_ = 1.8 Hz, 1H, H^2^), 7.45–7.22 (m, 9 H, aromatic), 6.85 (d, ^3^*J*_HH_ = 8.6 Hz, 2H, H^12^), 3.81 (s, 3H, OC*H*_3_), 2.69 (s, 6H, CH_3,_ Xyl), 1.36 (s, 9H, *^t^*Bu, C^∧^C), 1.34 (s, 9H, *^t^*Bu, C^∧^C). ^13^C{^1^H} NMR (CD_2_Cl_2_, 75 MHz, 298 K): δ 159.5 (C^1′^); 158.6 (C^14^); 152.7, 151.5, 151,4 (C^1^, C^6^, C^6′^), 151.1 (C≡NXyl), 150.3, 149.5 (C^3^, C^3′^), 136.7 (C^8^), 134.7 (C^2′^), 134.0 (C^2^), 132.6 (C^13^), 131.4 (C^10^), 128.5 (C^9^), 124.9 (C^7^), 124.5, 123.9, 120.6, 120.4 (C^4^, C^5^, C^4′^, C^5′^), 118.8 (C^11^), 114.7 (C^β^, C≡CC_6_H_4_OMe-4), 113.7 (C^12^), 55.2 (O*C*H_3_), 34.9 (*C*CH_3,_*^t^*Bu, C^∧^C), 34.7 (*C*CH_3,_*^t^*Bu, C^∧^C), 31,2 (*C*H_3,_*^t^*Bu, C^∧^C), 18.9 (*C*H_3,_ Xyl).

#### Synthesis of [{(C^∧^C)Au(C≡C*^t^*Bu)_2_}{Ag(C≡NXyl)}] **4a**

Method A: AgC≡C*^t^*Bu (25 mg, 0.13 mmol) was added to a solution of (C^∧^C)Au(C≡C*^t^*Bu)(C≡NXyl) (**3a**) (75 mg, 0.11 mmol) in CH_2_Cl_2_ (10 mL). The mixture was stirred in the dark at room temperature for 20 min. The volatile substances were removed affording an orange residue. The product was obtained as a white powder through precipitation from a CH_2_Cl_2_/diethyl ether mixture. (72 mg, 97%). Method B: A solution of (C^∧^C)AuCl(C≡NXyl) (80 mg, 0.13 mmol) in CH_2_Cl_2_ (20 mL) was treated with AgC≡C*^t^*Bu (50 mg, 0.26 mmol). The mixture was stirred at room temperature for 24 h in a dark flask and then filtered through a celite pad. The product was obtained as a white powder through precipitation from a CH_2_Cl_2_/diethyl ether mixture. (60 mg, 81%). Anal. Calcd for C_41_H_51_AgAuN (673.7): C, 57.08; H, 5.96; N, 1.62. Found: C, 56.86; H, 6.30; N, 1.55. IR (cm^–1^): ν(C≡N) 2170 (m). ν(C≡C) 2070 (vw). ^1^H NMR (CD_2_Cl_2_, 300.13 MHz, 298 K): δ 8.02 (d, ^4^*J*_HH_ = 1.6 Hz, 2H, H^2^), 7.35 (m, 1H, H^10^), 7.27 (m, 2H, H^5^), 7.21 (d, ^3^*J*_HH_ = 7.6 Hz, 2H, H^9^), 7.13 (dd, ^3^*J*_HH_ = 8.0 Hz, ^4^*J*_HH_ = 2.0 Hz, 2H, H^4^), 2.57 (s, 6H, CH_3,_ Xyl), 1.49 (s, 18H, *^t^*Bu, C≡C*^t^*Bu), 1.35 (s, 18H, *^t^*Bu, C^∧^C). ^13^C{^1^H} NMR (CD_2_Cl_2_, 75 MHz, 298 K): δ 154.5 (C^6^), 152.6 (C^1^), 151.6 (C≡NXyl), 148.8 (C^3^), 136.1 (C^8^), 132.8 (C^2^), 130.8 (C^10^), 128.4 (C^9^), 124.9 (C^β^, C≡C*^t^*Bu), 123.8 (C^7^), 123.0 (C^4^), 119.6 (C^5^), 87.3 (C^α^, C≡C*^t^*Bu), 34.8 (*C*CH_3,_*^t^*Bu, C^∧^C), 33.0 (*C*H_3,_*^t^*Bu, C≡C*^t^*Bu), 31.4 (*C*H_3,_*^t^*Bu, C^∧^C), 29.5 (*C*CH_3,_*^t^*Bu, C≡C*^t^*Bu), 18.7 (*C*H_3,_ Xyl).

#### Synthesis of [{(C^∧^C)Au(C≡CC_6_H_4_*^t^*Bu-4)_2_}{Ag(C≡NXyl)}] **4b**

The complex was prepared following the same synthetic procedure described for **4a** (Method A) but starting from AgC≡CC_6_H_4_*^t^*Bu-4 (35 mg, 0.13 mmol) and (C^∧^C)Au(C≡CC_6_H_4_*^t^*Bu-4)(C≡NXyl) (**3b**) (86 mg, 0.12 mmol). The product was obtained as a white powder through precipitation from a CH_2_Cl_2_/diethyl ether mixture. (112 mg, 92%). Anal. Calcd for C_53_H_59_AgAuN (1014.9): C, 62.72; H, 5.86; N, 1.38. Found: C, 62.86; H, 6.00; N, 1.22. IR (cm^–1^): ν(C≡N) 2173 (m). ν(C≡C) 2072 (vw). ^1^H NMR (CD_2_Cl_2_, 300.13 MHz, 298 K): δ 8.21 (bs, 2H, H^2^), 7.63 (d, ^3^*J*_HH_ = 7.6 Hz, 4H, H^12^), 7.36-7.27 (m, 6H, aromatic), 7.18–7.11 (m, 4H, aromatic), 2.21 (s, 6H, CH_3,Xyl_), 1.33 (s, 18H, *^t^*Bu), 1.30 (s, 18H, *^t^*Bu). ^13^C{^1^H} NMR (CD_2_Cl_2_, 75 MHz, 298 K): δ 154.7 (C^6^), 152.8 (C^1^), 151.7 (C^11/14^), 149.3 (C^3^), 136.3 (C^8^), 132.9 (C^2^), 132.3 (C^12/13^), 130.9 (C^10^), 128.5 (C^9^), 125.7 (C^12/13^), 124.9 (C^7^), 123.7 (C^4^), 121.2 (C^11/14^), 120.2 (C^5^), 113.8 (C^α^, C≡CC_6_H_4_*^t^*Bu-4), 35.0 (*C*CH_3,_*^t^*Bu, C^∧^C), 31.7 (*C*CH_3,_*^t^*Bu, C≡CC_6_H_4_*^t^*Bu-4), 31.2 (*C*H_3,_*^t^*Bu), 18.8 (*C*H_3,_ Xyl). C≡NXyl and C^β^ not observed.

#### Synthesis of [{(C^∧^C)Au(C≡CC_6_H_4_OMe-4)_2_}{Ag(C≡NXyl)}] **4c**

The complex was prepared following the same synthetic procedure described for **4a** (Method A) but starting from AgC≡CC_6_H_4_OMe-4 (30 mg, 0.13 mmol) and (C^∧^C)Au(C≡CC_6_H_4_OMe-4)(C≡NXyl) (**3c**) (80 mg, 0.11 mmol). The product was obtained as a white powder through precipitation from a CH_2_Cl_2_/diethyl ether mixture. (105 mg, 99%). Anal. Calcd for C_47_H_47_AgAuNO_2_ (962.72): C, 58.64; H, 4.92; N, 1.46. Found: C, 59.01; H, 4.99; N, 1.39. IR (cm^–1^): ν(C≡N) 2173 (m). ν(C≡C) 2074 (vw). ^1^H NMR (CD_2_Cl_2_, 300.13 MHz, 298 K): δ 8.18 (bd, 2H, H^2^), 7.60 (d, ^3^*J*_HH_ = 8.4 Hz, 4 H, H^13^), 7.31–7.10 (m, 7H, aromatic), 6.77 (d, ^3^*J*_HH_ = 8.4 Hz, 4H, H^12^), 3.77 (s, 6H, OC*H*_3_), 2.19 (s, 6H, CH_3,Xyl_), 1.30 (s, 18H, *^t^*Bu). ^13^C{^1^H} NMR (CD_2_Cl_2_, 75 MHz, 298 K): δ 159.9 (C^14^), 154.8 (C^6^), 152.8 (C^1^), 149.3 (C^3^), 136.3 (C^8^), 134.0 (C^13^), 132.9 (C^2^), 130.9 (C^10^), 128.4 (C^9^), 124.9 (C^7^), 123.8 (C^4^), 120.2 (C^5^), 116.2 (C^7^), 114.2 (C^12^), 98.6 (C^α^, C≡CC_6_H_4_OMe-4), 55.6 (O*C*H_3_), 35.0 (*C*CH_3,_*^t^*Bu, C^∧^C), 31.7 (*C*H_3,_*^t^*Bu, C^∧^C), 18.7 (*C*H_3,_ Xyl). C^β^ and *C*≡NXyl not observed.

#### Synthesis of [{(C^∧^C)Au (C≡C*^t^*Bu)_2_}{Cu(C≡NXyl)}] **5a**

[{(C^∧^C)Au(C≡C*^t^*Bu)_2_}{Ag(C≡NXyl)}] **4a** (21.6 mg, 0.025 mmol) and CuCl (2.5 mg, 0.025 mmol) were stirred in CH_2_Cl_2_ (2 mL) overnight at room temperature. The solution was filtered through celite, and the solvent evaporated affording a whitish residue. The product was obtained as a white powder through crash-out with diethyl ether (13 mg, 0.016 mmol, 65% yield). Anal. Calcd for C_41_H_51_NAuCu (818.4): C, 60.17; H, 6.28; N, 1.71. Found: C, 59.98; H, 6.50; N, 1.63. IR (cm^–1^): ν(C≡N) 2156 (m). ν(C≡C) Not observed. ^1^H NMR (CD_2_Cl_2_, 300.13 MHz, 298 K): δ 7.91 (s, 2H, H^2^), 7.35-7.13 (m, 7H, aromatic), 2.48 (s, 6H, CH_3,Xyl_), 1.49 (s, 18H, *^t^*Bu, C≡C*^t^*Bu), 1.34 (s, 18H, *^t^*Bu_,_ C^∧^C). ^13^C{^1^H} NMR (CD_2_Cl_2_, 75 MHz, 298 K): δ 154.7 (C^6^), 153.0 (C^1^), 149.3 (C^3^), 136.0 (C^8^), 133.2 (C^2^), 130.6 (C^14^), 128.7 (C^13^), 126.4 (C^β^, C≡C*^t^*Bu), 125.8 (C^11^), 123.5 (C^4^), 120.1 (C^5^), 87.7 (C^α^, C≡C*^t^*Bu), 35.2 (CCH_3,*^t^*Bu_, C^∧^C), 32.8 (CH_3,_*^t^*Bu, C≡C*^t^*Bu), 31.8 (CH_3,_*^t^*Bu, C^∧^C), 30.8 (CCH_3,_*^t^*Bu, C≡C*^t^*Bu), 19.1 (CH_3,_ Xyl). C≡NXyl not observed.

#### Synthesis of [{(C^∧^C)Au(C≡CC_6_H_4_*^t^*Bu-4)_2_}{Cu(C≡NXyl)}] **5b**

The complex was prepared following the same method described for **5a** but starting from [{(C^∧^C)Au(C≡CC_6_H_4_*^t^*Bu-4)_2_}{Ag(C≡NXyl)}] **4b** (25.4 mg, 0.025 mmol) and CuCl (2.5 mg, 0.025 mmol). The product was obtained as a white solid (19.6 mg, 0.020 mmol, 81% yield). Anal. Calcd for C_53_H_59_NAuCu (970.6): C, 65.59; H, 6.13; N, 1.44. Found: C, 65.21; H, 6.33; N, 1.82. IR (cm^–1^): ν(C≡N) 2161 (m). ν(C≡C) Not observed. ^1^H NMR (CD_2_Cl_2_, 300.13 MHz, 298 K): δ 8.08 (bd, ^3^*J*_HH_ = 1.7 Hz, 2H, H^2^), 7.64 (d, ^3^*J*_HH_ = 8.3 Hz, 4H, H^12^), 7.35 (d, ^3^*J*_HH_ = 8.3 Hz, 4H, H^13^), 7.31–7.16 (m, 5H, aromatic), 7.04 (d, ^3^*J*_HH_ = 7.6 Hz, 2H, H^9^), 2.00 (s, 6H, CH_3,Xyl_), 1.35 (s, 18H, *^t^*Bu), 1.30 (s, 18H, *^t^*Bu). ^13^C{^1^H} NMR (CD_2_Cl_2_, 75 MHz, 298 K): δ 154.6 (C^6^), 152.8 (C^1^), 152.0 (C^11/14^), 149.5 (C^3^), 136.2 (C^8^), 133.1 (C^2^), 132.1 (C^12/13^), 130.5 (C^10^), 128.3 (C^9^), 125.9 (C^12/13^), 125.5 (C^7^), 123.8 (C^4^), 121.8 (C^11/14^), 120.3 (C^5^), 116.1 (C^β^, C≡CC_6_H_4_*^t^*Bu-4), 98.2 (C^α^, C≡CC_6_H_4_*^t^*Bu-4), 36.1 (*C*CH_3,_*^t^*Bu), 36.0 (*C*CH_3,_*^t^*Bu), 31.7 (*C*H_3,_*^t^*Bu), 31.3 (*C*H_3,_*^t^*Bu), 18.6 (*C*H_3,_ Xyl). *C*≡NXyl not observed.

#### Synthesis of [{(C^∧^C)Au(C≡CC_6_H_4_OMe-4)_2_}{Cu(C≡NXyl)}] **5c**

The complex was prepared following the same method described for **5a** but starting from [{(C^∧^C)Au(C≡CC_6_H_4_OMe-4)_2_}{Ag(C≡NXyl)}] **4a** (24.1 mg, 0.025 mmol) and CuCl (2.5 mg, 0.025 mmol). The product was obtained as a white solid (18.7 mg, 0.020 mmol, 81% yield). Anal. Calcd for C_47_H_47_AuCuNO_2_ (918.4): C, 61.47; H, 5.16; N, 1.53. Found: C, 61.22; H, 5.69; N, 1.41. IR (cm^–1^): ν(C≡N) 2161 (m). ν(C≡C) 2060 (vw). ^1^H NMR (CD_2_Cl_2_, 300.13 MHz, 298 K): δ 8.07 (bd, 2H, H^2^), 7.63 (d, ^3^*J*_HH_ = 8.4 Hz, 4 H, H^13^), 7.29 (d, ^3^*J*_HH_ = 8.0 Hz, 2H, H^5^), 7.26–7.20 (m, 1H, H^10^), 7.17 (d, ^3^*J*_HH_ = 7.9 Hz, 2H, H^4^), 7.06 (d, ^3^*J*_HH_ = 7.6 Hz, 2H, H^9^), 6.83 (d, ^3^*J*_HH_ = 8.0 Hz, 4 H, H^12^), 3.78 (s, 6 H, OC*H*_3_), 2.04 (s, 6H, CH_3,Xyl_), 1.33 (s, 18H, *^t^*Bu). ^13^C{^1^H} NMR (CD_2_Cl_2_, 75 MHz, 298 K): δ 160.2 (C^14^), 154.7 (C^6^), 152.8 (C^1^), 149.4 (C^3^), 136.2 (C^8^), 133.8 (C^12/13^), 133.0 (C^2^), 130.5 (C^10^), 128.4 (C^9^), 125.6 (C^7^), 123.8 (C^4^), 120.3 (C^5^), 116.8 (C^7^), 114.3 (C^β^, C≡CC_6_H_4_OMe-4), 114.3 (C^12/13^), 97.8 (C^α^, C≡CC_6_H_4_OMe-4), 55.7 (OCH_3_), 35.0 (CCH_3,_*^t^*Bu, C^∧^C), 31.6 (CH_3,_*^t^*Bu, C^∧^C), 18.5 (CH_3,_ Xyl). C≡NXyl not observed.

## Supporting Information Available

Details of synthesis and characterization; X-ray crystallography; photophysical properties; and theoretical calculations (PDF)

## Dedication

^†^ Dedicated to Prof. E. J. Fernández, colleague, father, and friend.
