# Supplementary material for: Luminescent Au(III)–M(I) (M = Cu, Ag) Aggregates Based on Dicyclometalated Bis(alkynyl) Gold Anions†
Source: Inorg Chem. 2023 Aug 3;62(32):12683–96. doi: 10.1021/acs.inorgchem.3c00870 (PMC10428224; doi:10.1021/acs.inorgchem.3c00870)
Supplement: Supplementary file 1 — ic3c00870_si_001.pdf [file ic3c00870_si_001.pdf]

Supporting Information

Luminescent Au(III)-M(I) (M= Cu, Ag) Aggregates Based on Dicyclometalated Bis(alkynyl) Gold Anions.

Rebeca Lara Garnica,[a,b] Raquel J. Rama,[a,d] Isabelle Chambrier,[a] Gabriele Agonigi,[a,c] David L. Hughes,[a] Elena Lalinde,[b] Manfred Bochmann[a] and Julio Fernandez-Cestau[a,b]*

[a] Dr. I. Chambrier https://orcid.org/0000-0003-0090-1186, Dr D. L. Hughes, https://orcid.org/0000-0003-0621-204X, Prof. Dr. M. Bochmann https://orcid.org/0000-0001-7736-5428, School of Chemistry, University of East Anglia, Norwich Research Park, Norwich NR4 7TJ, UK.

[b] Dr. R. Lara Garnica, Prof. Dr. E. Lalinde https://orcid.org/0000-0001-7402-1742, Dr. J. Fernandez-Cestau https://orcid.org/0000-0001-7663-6222, Departamento de Química - Centro de Investigación en Síntesis Química (CISQ), Universidad de La Rioja, E-26006 Logroño (Spain)

E-mail: juliofernandez50@gmail.com

[c] Dr. G. Agonigi, Dipartimento di Chimica e Chimica Industriale, University of Pisa, I-56124 Pisa (Italy)

[d] Dr. R. J. Rama, https://orcid.org/0000-0001-9586-1599 Departamento de Química Inorgánica, Universidad de Sevilla, 41071 Sevilla, (Spain); SMN Centre for Materials Science and Nanotechnology, Department of Chemistry, University of Oslo, Sem Sælands vei 26, 0371 Oslo, (Norway).

NMR **S1**

X-ray crystallography **S2**

Photophysical properties **S3**

Theoretical calculations **S4**

References **S5**

S1. NMR

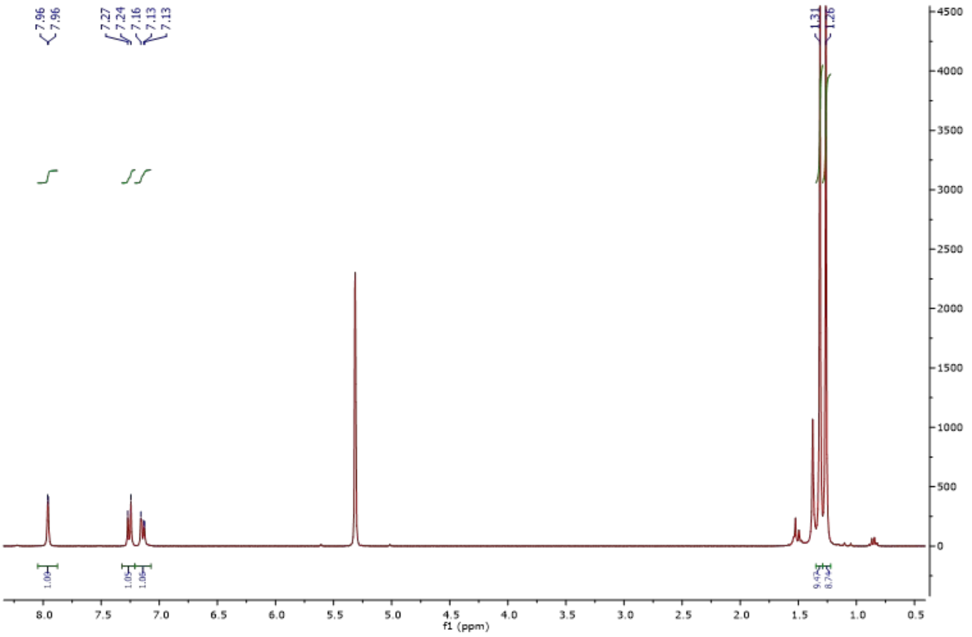

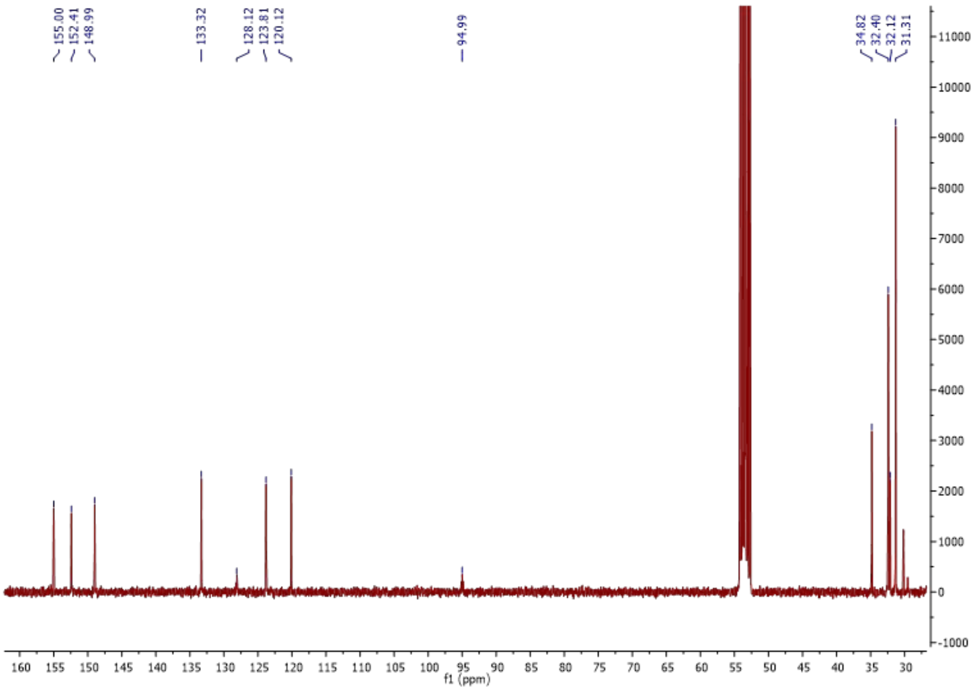

Figure S1. ^{1}H and $^{13}C\{^{1}H\}$ NMR spectra of $[\{(C\text{^}C)Au(C{\equiv}C^{t}Bu)_2\}_2Ag_2]$ **1.**

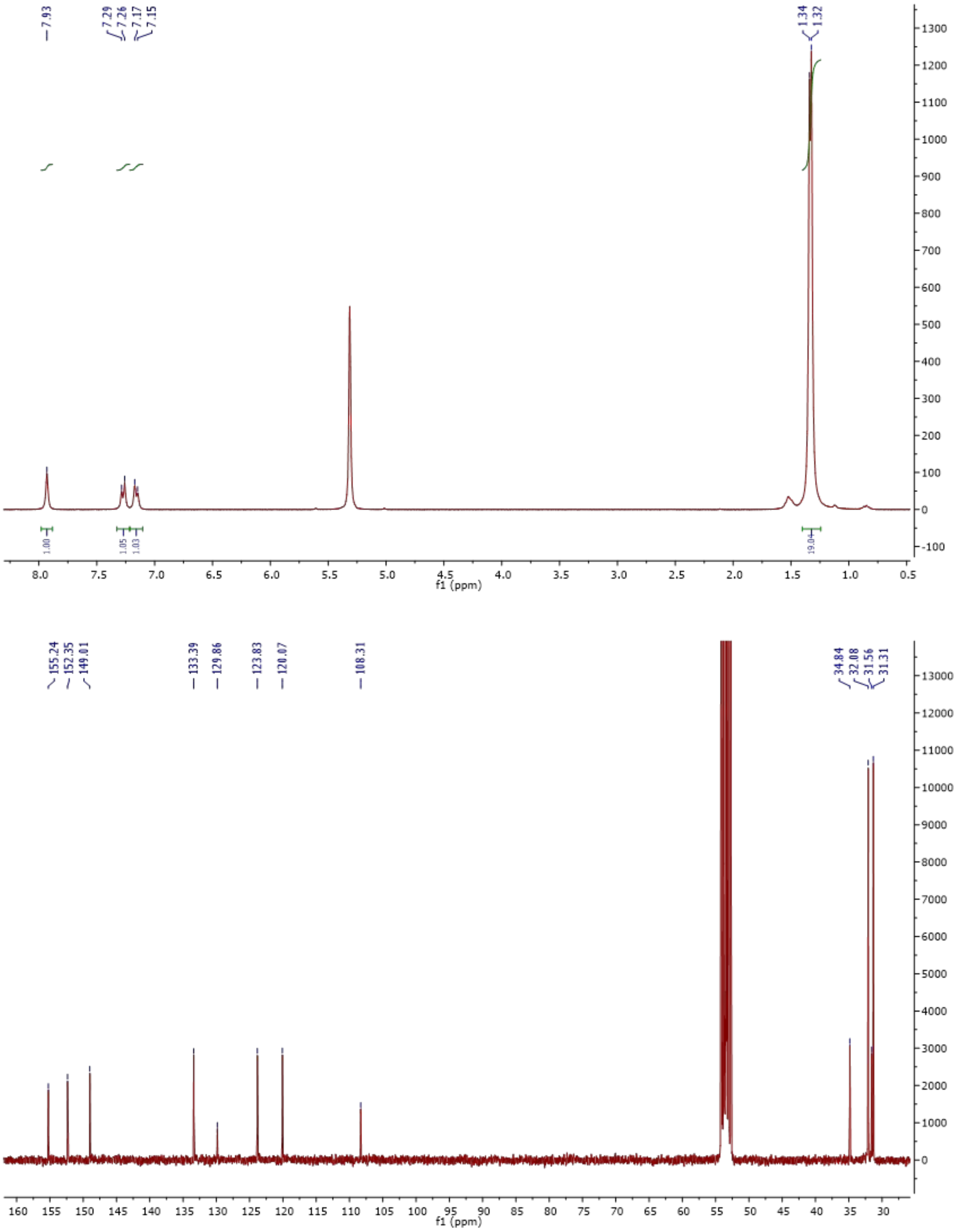

Figure S2. ^{1}H and $^{13}C\{^{1}H\}$ NMR spectra of [{(C^C)Au(C≡C^{t}Bu)$_2$}$_2$Cu$_2$] **2.**

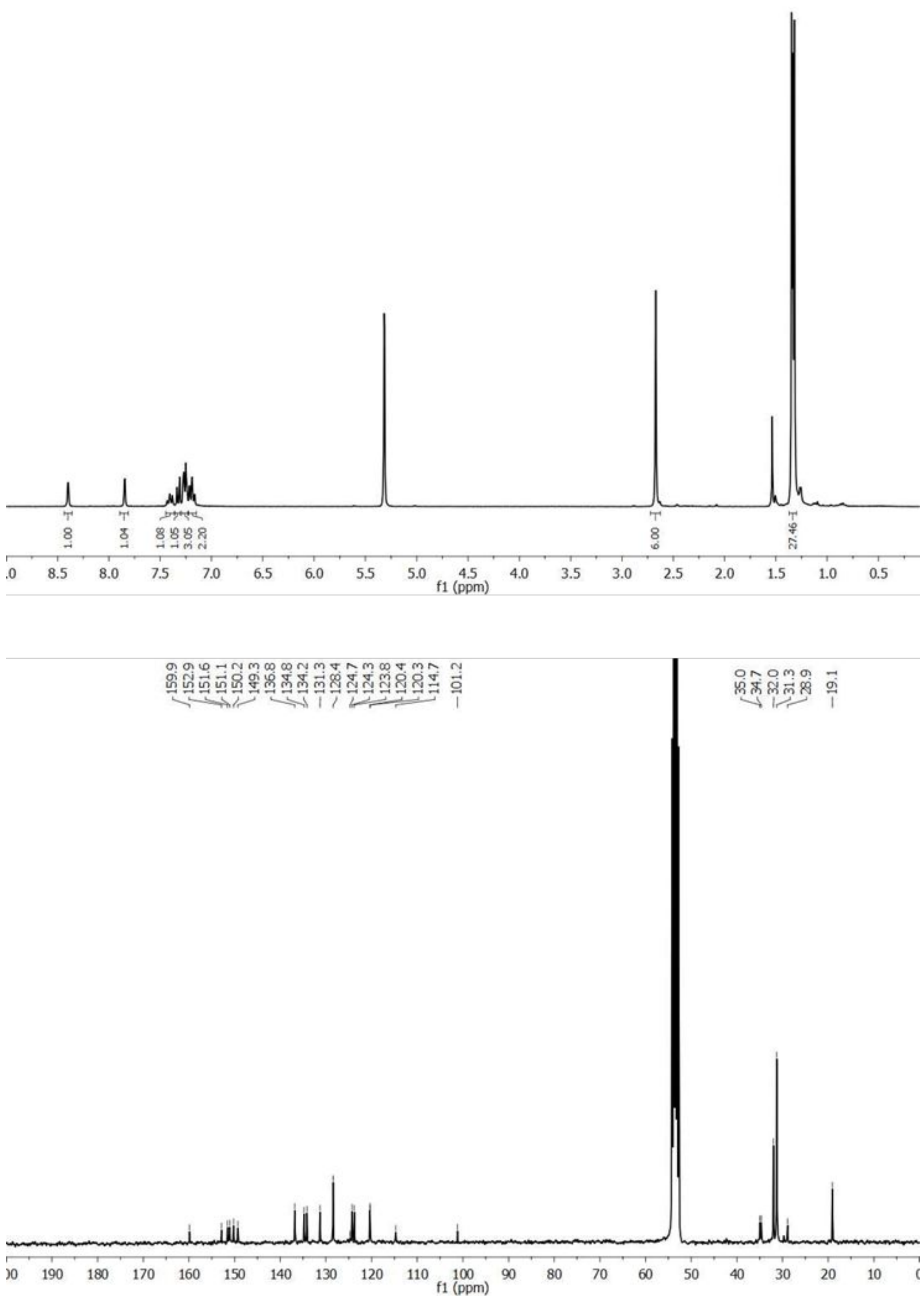

Figure S3. ^{1}H and ^{13}C{^{1}H] NMR spectra of (C^C)Au(C≡C^{t}Bu)(C≡NXyl) **3a.**

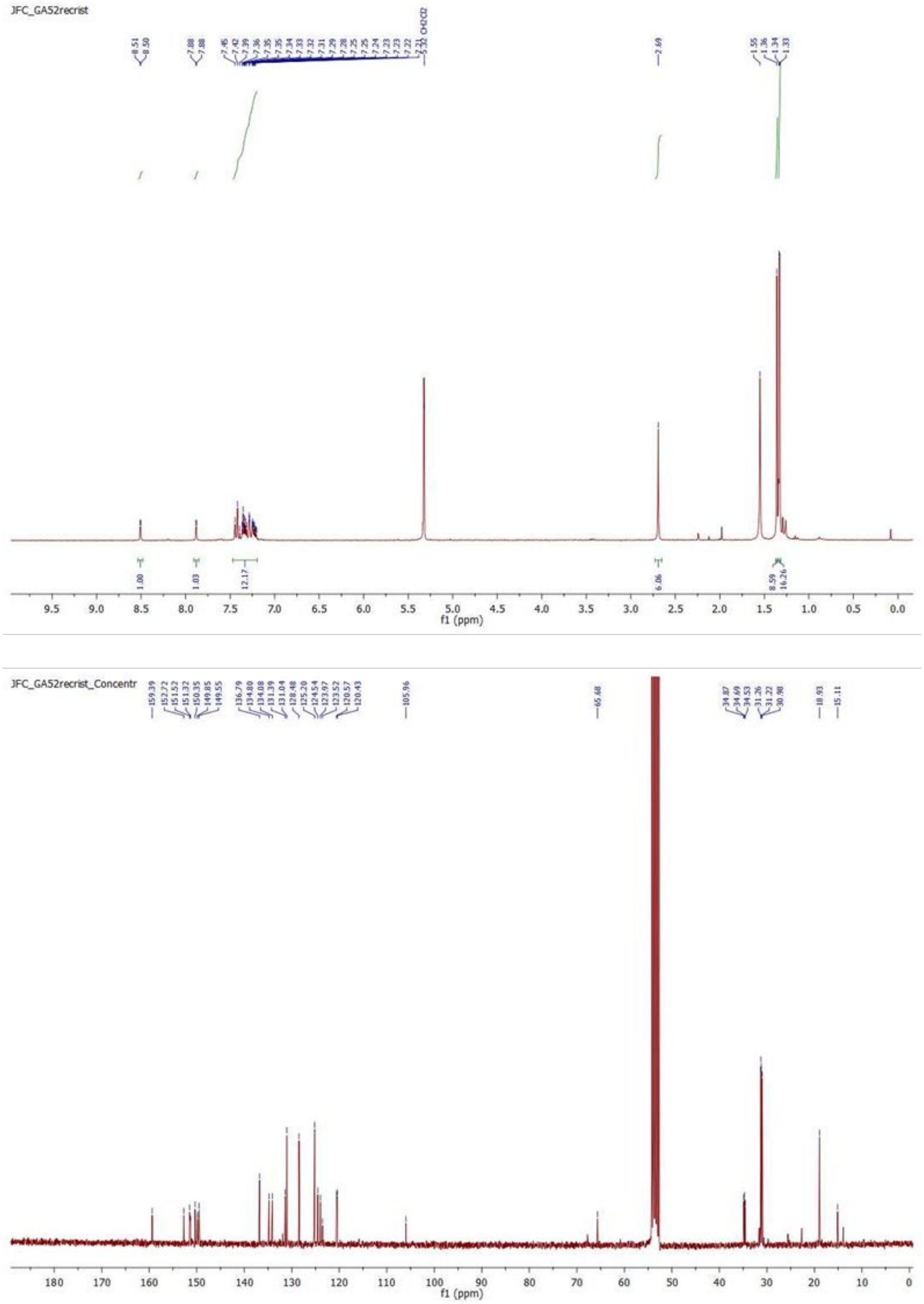

Figure S4. ^{1}H and ^{13}C{^{1}H] NMR spectra of (C^C)Au(C≡CC$_6$H$_4$^{t}Bu-4)(C≡NXyl) **3b.**

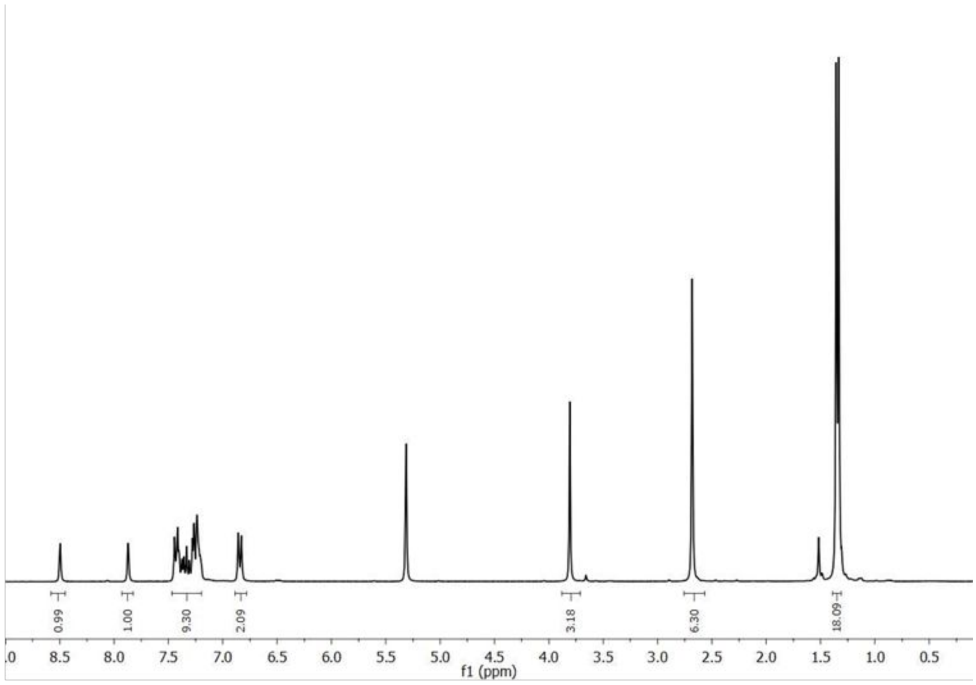

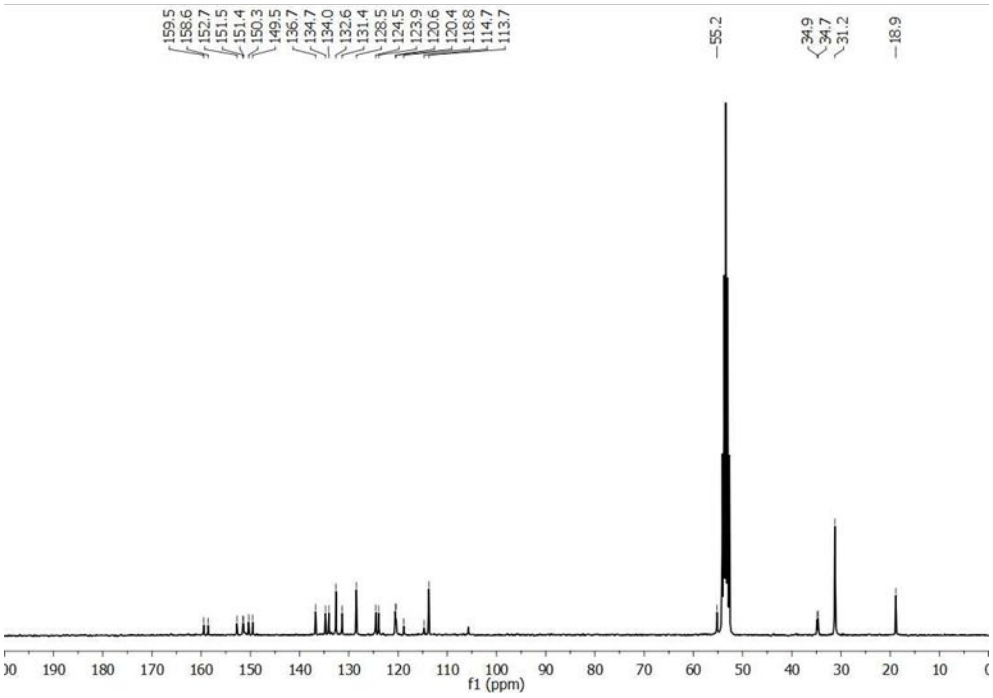

Figure S5. ^{1}H and $^{13}C\{^{1}H]$ NMR spectra of (C^C)Au(C≡CC$_6$H$_4$OMe-4)(C≡NXyl) **3c.**

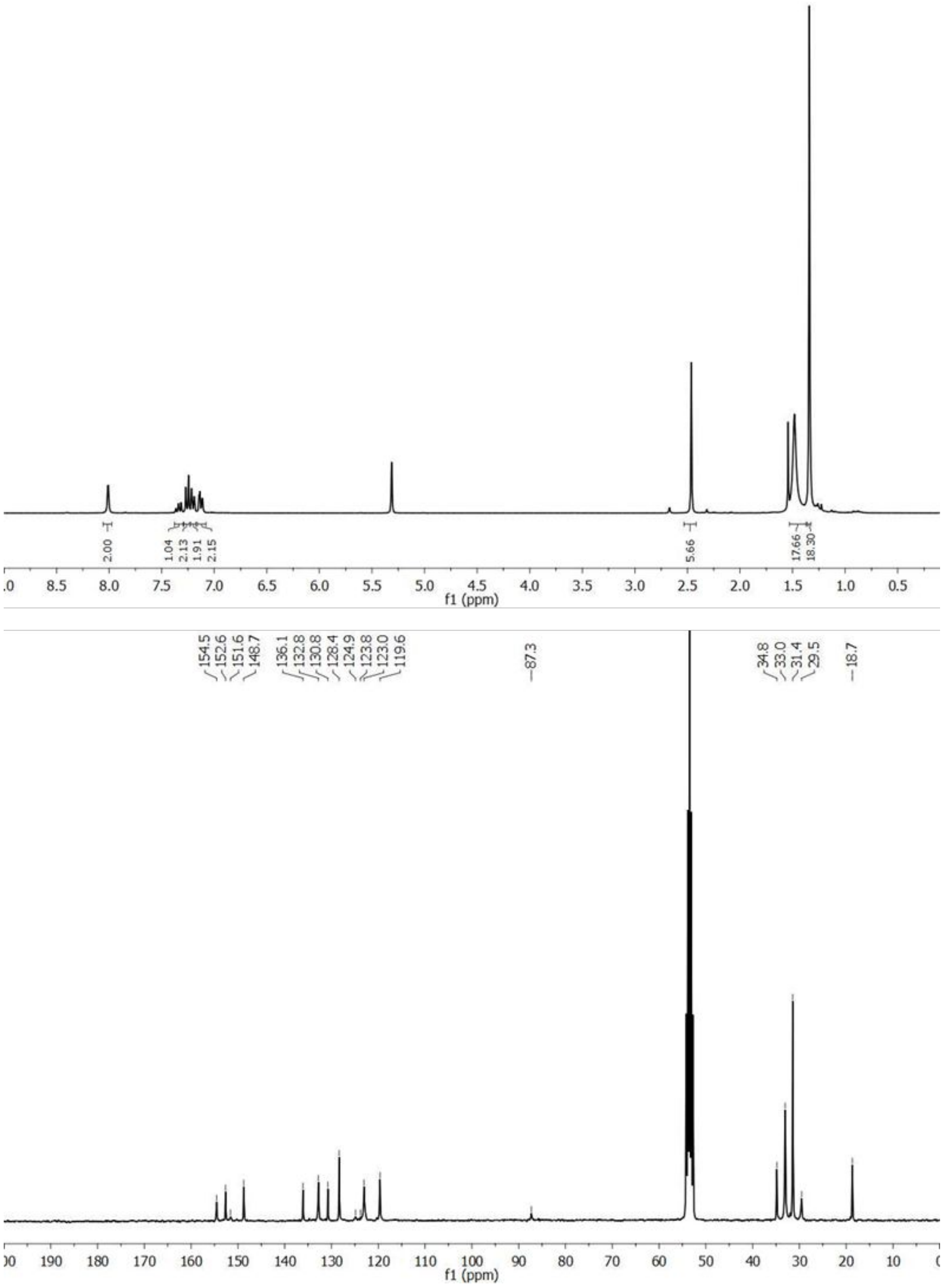

Figure S6. ^{1}H and ^{13}C{^{1}H] NMR spectra of [{(C^C)Au(C≡C^{t}Bu)$_2$}{Ag(C≡NXyl)}] **4a.**

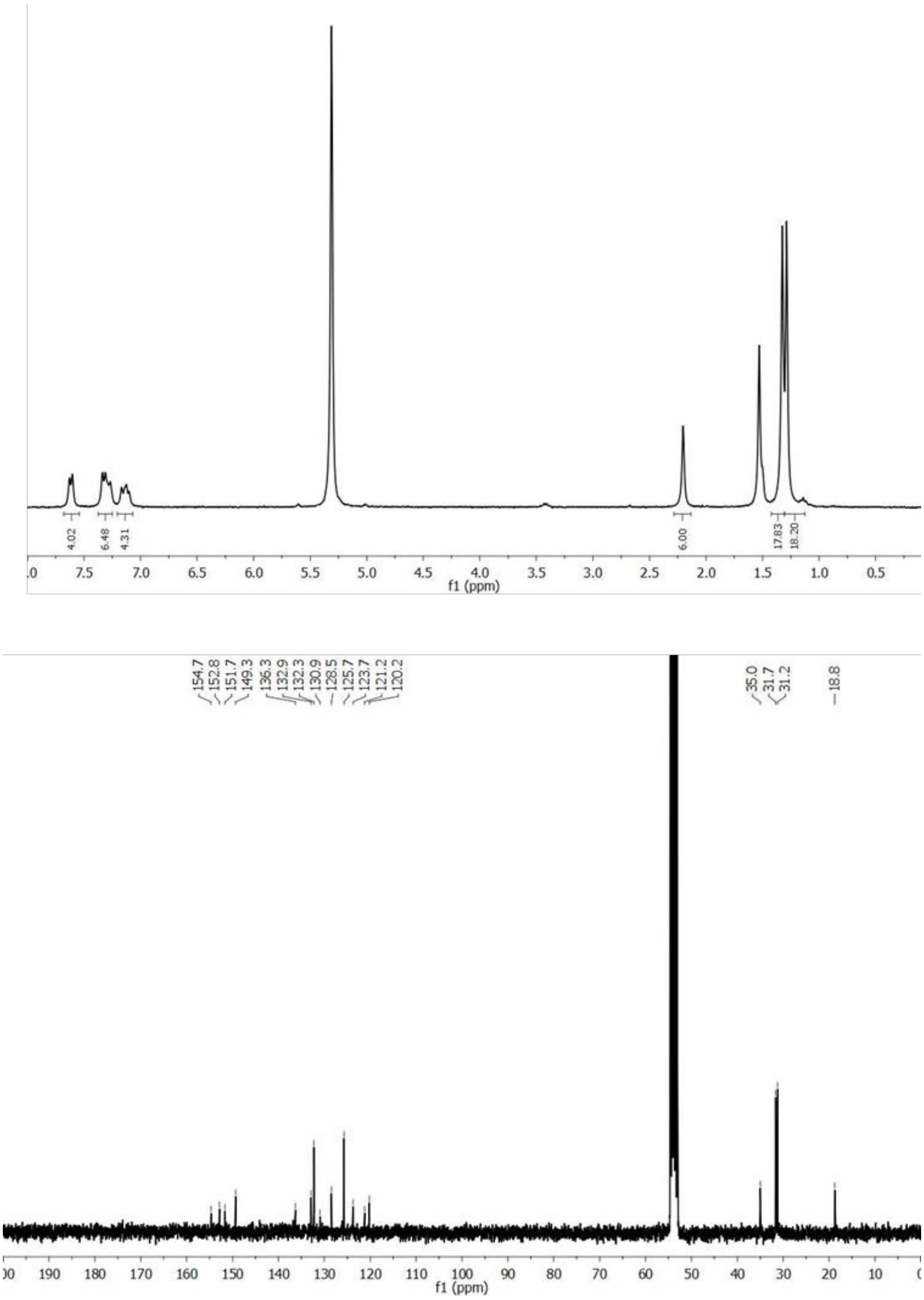

Figure S7. ^{1}H and $^{13}C\{^{1}H\}$ NMR spectra of [{(C^C)Au(C≡CC$_6$H$_4$^{t}Bu-4)$_2$}{Ag(C≡NXyl)}] **4b.**

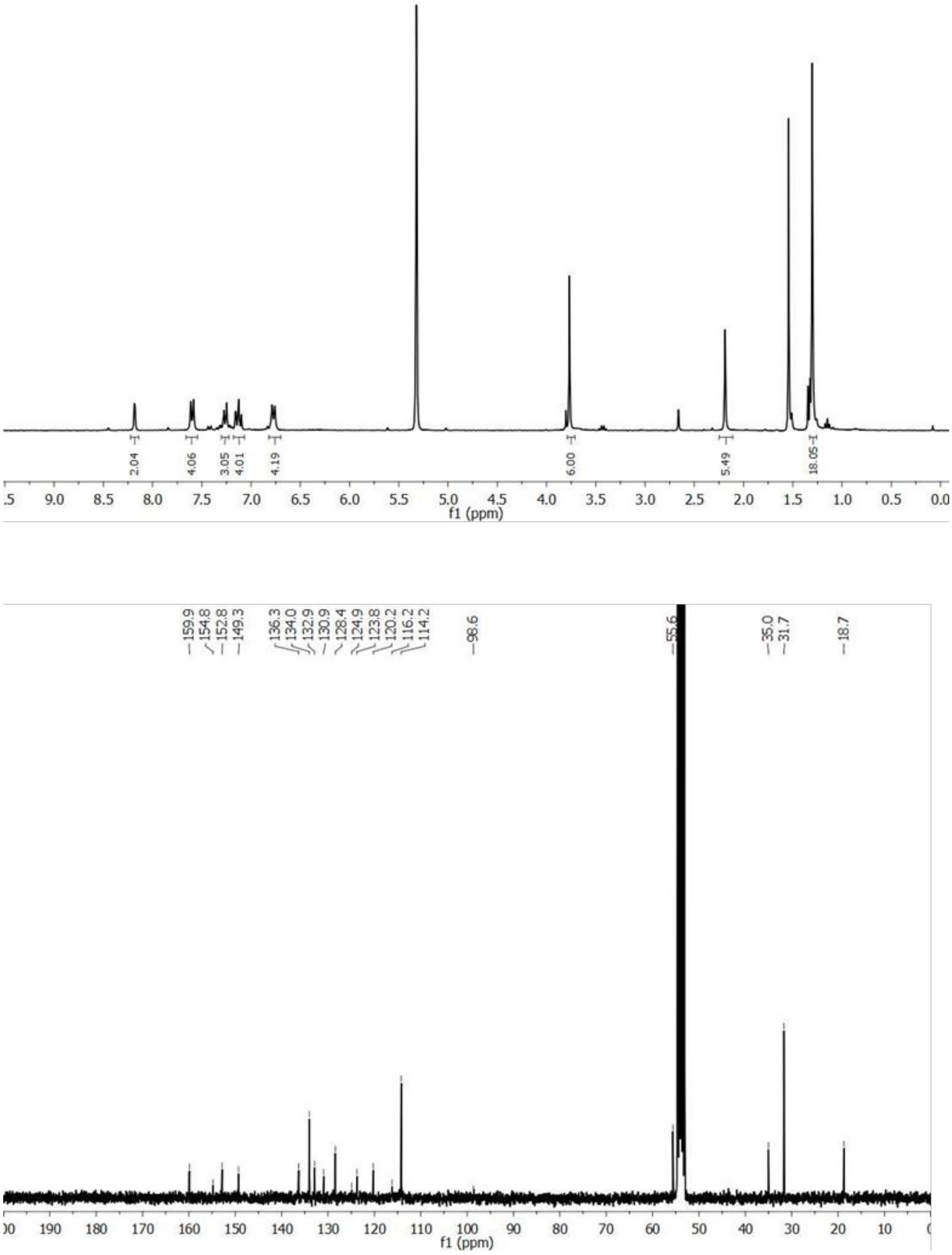

Figure S8. ^{1}H and $^{13}C\{^{1}H\}$ NMR spectra of [{(C^C)Au(C≡CC$_6$H$_4$OMe-4)$_2$}{Ag(C≡NXyl)}] **4c.**

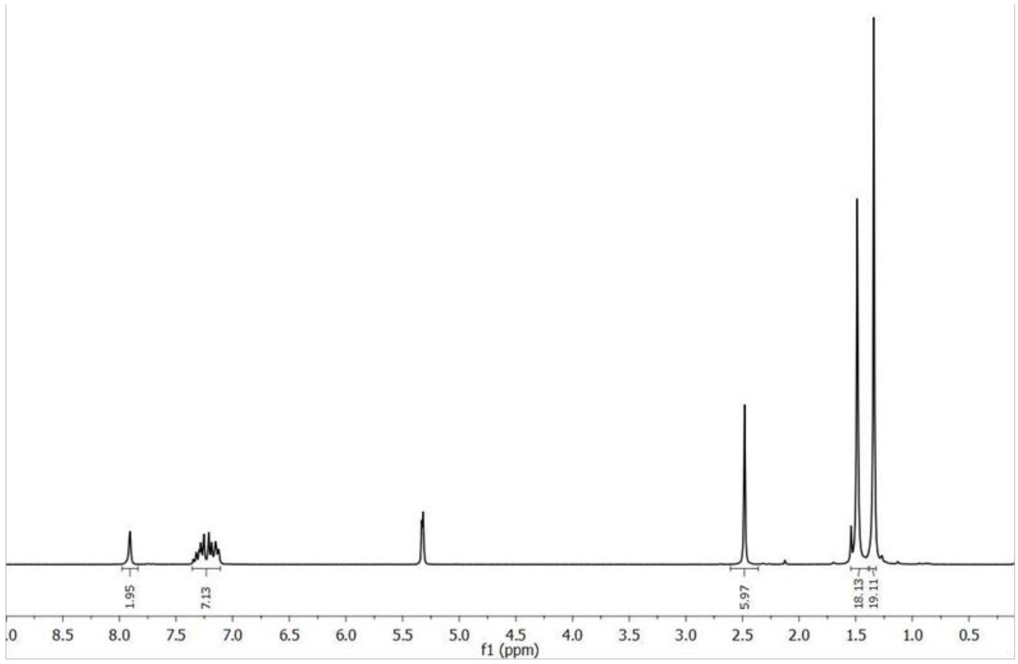

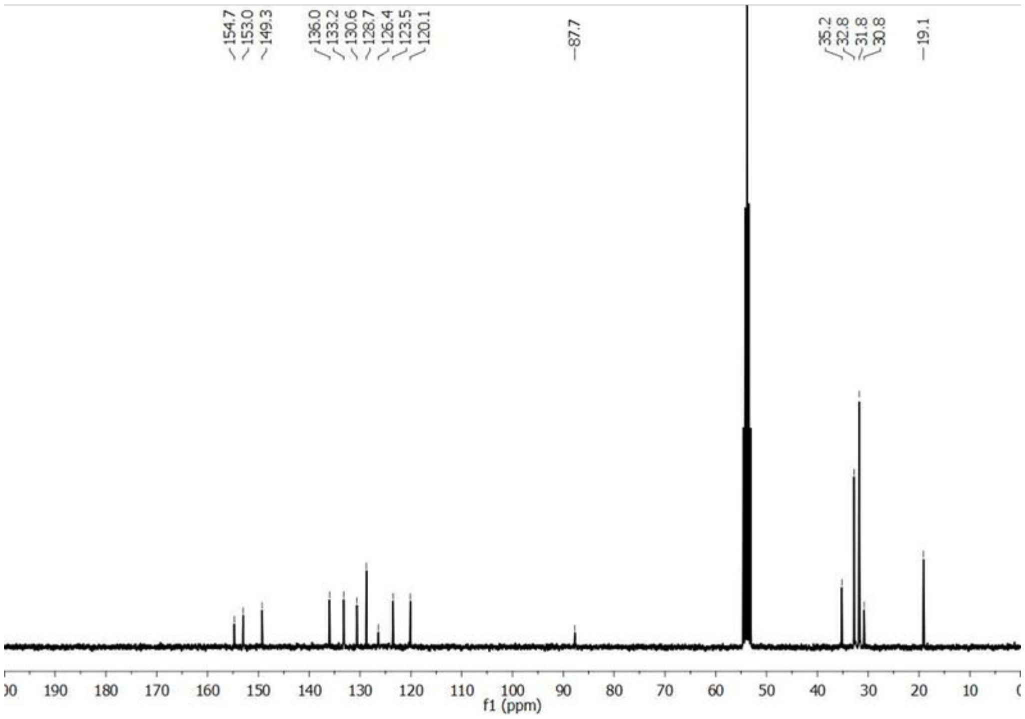

Figure S9. ^{1}H and $^{13}C\{^{1}H\}$ NMR spectra of [{(C^C)Au (C≡C^{t}Bu)$_2$}{Cu(C≡NXyl)}] **5a**.

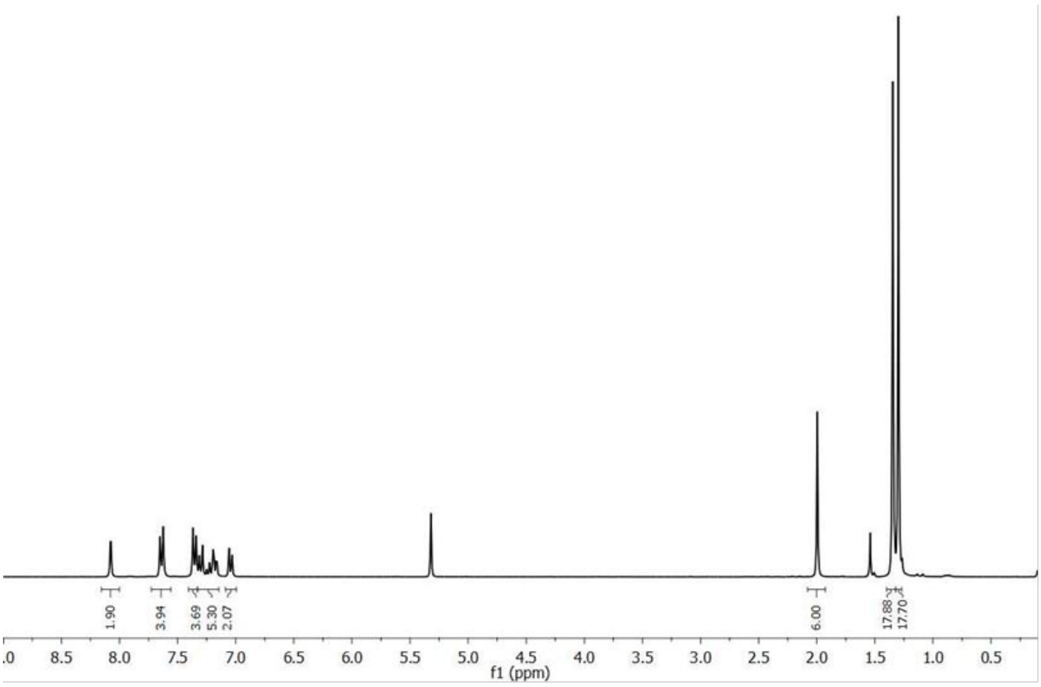

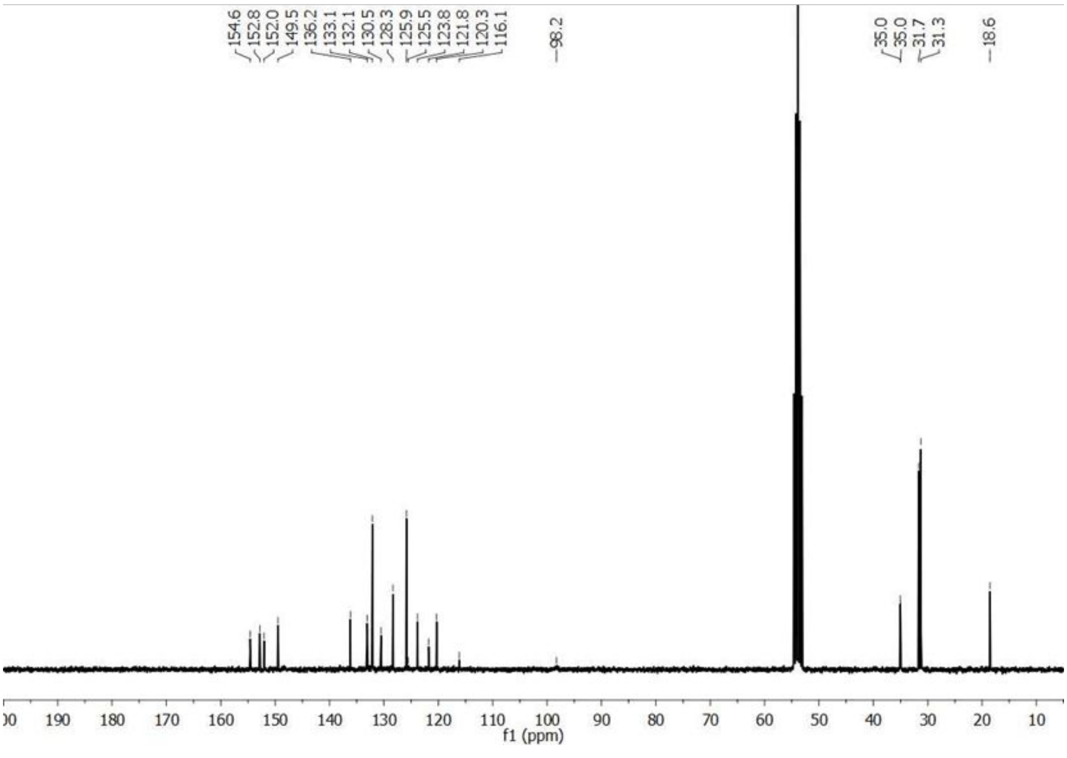

Figure S10. ^{1}H and $^{13}C\{^{1}H\}$ NMR spectra of $[\{(C\text{^}C)Au(C{\equiv}CC_6H_4{}^tBu\text{-}4)_2\}\{Cu(C{\equiv}NXyl)\}]$ **5b.**

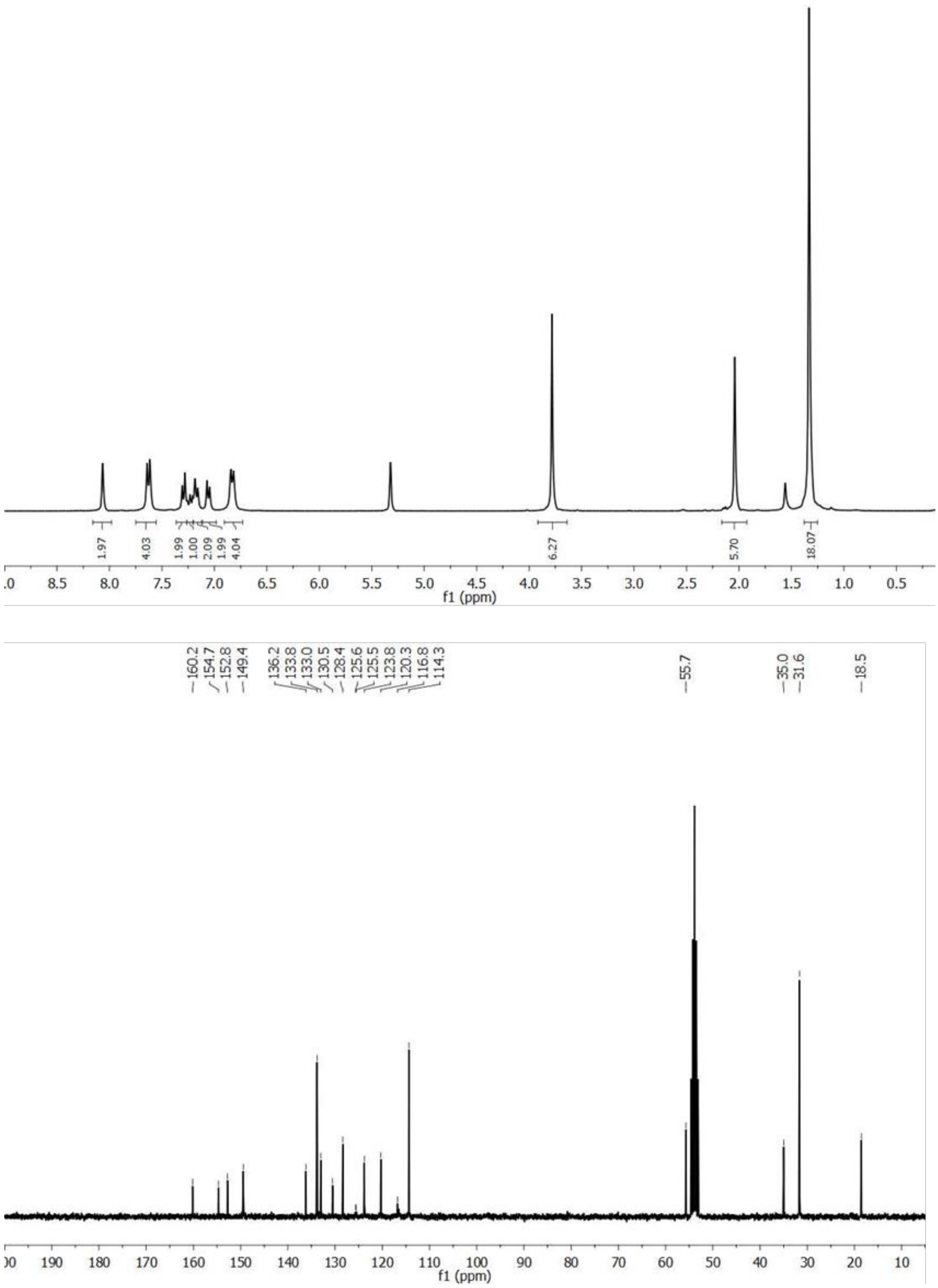

Figure S11. ^{1}H and ^{13}C{^{1}H} NMR spectra of [{(C^C)Au(C≡CC$_6$H$_4$OMe-4)$_2$}{Cu(C≡NXyl)}] **5c.**

S2. X-ray crystallography

Crystals of each sample were mounted in MiTeGen MicroMesh systems and fixed in the cold nitrogen stream on a diffractometer. Diffraction intensities were recorded al low temperature on an Oxford Diffraction Xcalibur-3/Sapphire3-CCD diffractometer, equipped with Mo-Kα radiation and graphite monochromator or Rigaku HG Saturn724+ (2×2 bin mode). Data were processed using the CrystAlisPro-CCD and –RED software or CrystalClear-SM Expert 3.1 b27, been the absorption correction done at this stage.[S1]

The structures of all samples were determined by the direct methods routines with SHELXS or SHELXT programs and refined by full-matrix least-squares methods on F^2 in SHELXL.[S2] Non-hydrogen atoms were generally refined with anisotropic thermal parameters. Hydrogen atoms were included in idealized positions. No missed symmetry was reported by PLATON.[S3]

Refinement results are included in Table S1. Computer programs used in this analysis were run through[S4] Scattering factors for neutral atoms were taken from reference [S5]

Crystallographic details

Crystal structure analysis of . [{(C^C)Au(C≡C^{t}Bu)$_2$}$_2$Ag$_2$] 1:

Crystal data: $C_{32}H_{42}AuAg$, M = 731.49. Monoclinic, space group $P2_1/c$ (no. 14), a = 14.9787(3), b = 10.8556(2), c = 19.7012(5) Å, β = 110.724(3) °, V = 2996.19(12) Å^3. Z = 4, Dc = 1.622 g cm^{-3}, F(000) = 1440, T = 140(2) K, μ(Mo-Kα) = 55.6 cm^{-1}, λ(Mo-Kα) = 0.71073 Å.

The crystal was a light yellow block. From a sample under oil, one, *ca* 0.10 x 0.10 x 0.20 mm, was mounted on a small loop and fixed in the cold nitrogen stream on a Rigaku Oxford Diffraction Xcalibur diffractometer, equipped with Mo-Kα radiation, Sapphire-3 detector and graphite monochromator. Intensity data were measured by thin-slice ω-scans. Total no. of reflections recorded, to θ_{max} = 27.5°, was 49,803 of which 6867 were unique (Rint = 0.041); 6301 were 'observed' with $I > 2\sigma_I$.

Data were processed using the CrysAlisPro-CCD and -RED (S1) programs. The structure was determined by the intrinsic phasing routines in the SHELXT program (S2) and refined by full-matrix least-squares methods, on F^2's, in SHELXL (S2). The ^{t}Bu group of C(17) is disordered in two orientations with an occupation ratio of 0.893 : 0.107. The non-hydrogen atoms (except

for the minor components of the disordered ^{t}Bu group) were refined with anisotropic thermal parameters. The hydrogen atoms were included in idealised positions and their Uiso values were set to ride on the Ueq values of the parent carbon atoms. At the conclusion of the refinement, $wR_2 = 0.043$ and $R_1 = 0.024$ (2B) for all 6867 reflections weighted $w = [\sigma^2(F_o^2) + (0.0160\ P)^2 + 1.6783\ P]^{-1}$ with $P = (F_o^2 + 2F_c^2)/3$; for the 'observed' data only, $R_1 = 0.020$.

In the final difference map, the highest peak (*ca* 0.87 e$Å^{-3}$) was near the Au centre.

Scattering factors for neutral atoms were taken from reference (S5). Computer programs used in this analysis have been noted above, and were run through WinGX (S4) on a Dell Optiplex 780 PC at the University of East Anglia.

Crystal structure analysis of [{(C^C)Au(C≡C^{t}Bu)$_2$}$_2$Cu$_2$] 2

Crystal data: $C_{32}H_{42}AuCu$, M = 687.16. Monoclinic, space group $P2_1/n$ (equiv. to no. 14), a = 14.8997(3), b = 10.57203(15), c = 20.0210(4) Å, β = 110.542(2) °, V = 2953.17(10) $Å^3$. Z = 4, Dc = 1.546 g cm^{-3}, F(000) = 1368, T = 104(2) K, μ(Mo-Kα) = 57.0 cm^{-1}, λ(Mo-Kα) = 0.71073 Å.

The crystal was a colourless block. From a sample under oil, one, *ca* 0.10 x 0.10 x 0.15 mm, was mounted on a small loop and fixed in the cold nitrogen stream on a Rigaku Oxford Diffraction XtaLAB AFC12 (RCD3) diffractometer, equipped with Mo-Kα radiation, CCD plate detector and mirror monochromator. Intensity data were measured by thin-slice ω-scans. Total no. of reflections recorded, to $\theta_{max} = 27.5°$, was 114,568 of which 6772 were unique (Rint = 0.049); 6507 were 'observed' with $I > 2\sigma_I$.

Data were processed using the CrysAlisPro-CCD and -RED (S1) programs. The structure was determined by the intrinsic phasing routines in the SHELXT program (S2) and refined by full-matrix least-squares methods, on F^2's, in SHELXL (S2). The non-hydrogen atoms were refined with anisotropic thermal parameters. The hydrogen atoms were included in idealised positions and their Uiso values were set to ride on the Ueq values of the parent carbon atoms. At the conclusion of the refinement, $wR_2 = 0.047$ and $R_1 = 0.023$ (2B) for all 6772 reflections weighted $w = [\sigma^2(F_o^2) + (0.0029\ P)^2 + 10.0221\ P]^{-1}$ with $P = (F_o^2 + 2F_c^2)/3$; for the 'observed' data only, $R_1 = 0.021$.

In the final difference map, the highest peak (*ca* 1.3 e$Å^{-3}$) was near H(25b).

Scattering factors for neutral atoms were taken from reference (S5). Computer programs used in this analysis have been noted above, and were run through WinGX (S4) on a Dell Optiplex 780 PC at the University of East Anglia.

Crystal structure analysis of [{(C^C)Au(C≡CtBu)$_2$}{Ag(C≡NXyl)}] 4a

Crystal data: $C_{41}H_{51}AgAuN$, M = 862.66. Monoclinic, space group $P2_1/c$ (no. 14), a = 28.2944(5), b = 13.2307(2), c = 21.4762(3) Å, β = 111.396(2) °, V = 7485.6(2) Å^3. Z = 4, Dc = 1.531 g cm^{-3}, F(000) = 3440, T = 105.1(6) K, μ(Mo-Kα) = 44.7 cm^{-1}, λ(Mo-Kα) = 0.71073 Å.

The crystal was a colourless plate. From a sample under oil, one, *ca* 0.20 x 0.15 x 0.05 mm, was mounted on a small loop and fixed in the cold nitrogen stream on a Rigaku Oxford Diffraction XtaLAB Synergy diffractometer, equipped with Mo-Kα radiation, HyPix detector and mirror monochromator. Intensity data were measured by thin-slice ω-scans. Total no. of reflections recorded, to θ_{max} = 27.5°, was 87,245 of which 17,129 were unique (Rint = 0.044); 15,244 were 'observed' with I > $2\sigma_I$.

Data were processed using the CrysAlisPro-CCD and -RED (S1) programs. The structure was determined by the intrinsic phasing routines in the SHELXT program (S2) and refined by full-matrix least-squares methods, on F^2's, in SHELXL (S2). The non-hydrogen atoms were refined with anisotropic thermal parameters. The hydrogen atoms were included in idealised positions and their Uiso values were set to ride on the Ueq values of the parent carbon atoms. At the conclusion of the refinement, wR_2 = 0.070 and R_1 = 0.038 (2B) for all 17,129 reflections weighted $w = [\sigma^2(F_o^2) + (0.0290 P)^2 + 16.8113 P]^{-1}$ with $P = (F_o^2 + 2F_c^2)/3$; for the 'observed' data only, R_1 = 0.031.

In the final difference map, the highest peak (*ca* 1.6 eÅ^{-3}) was near Au(2).

Scattering factors for neutral atoms were taken from reference (S5). Computer programs used in this analysis have been noted above, and were run through WinGX (S4) on a Dell Optiplex 780 PC at the University of East Anglia.

Crystal structure analysis of [{(C^C)Au(C≡CC$_6$H$_4$OMe-4)$_2$}{Ag(C≡NXyl)}]$_2$·CH$_2$Cl$_2$ **4c$_2$**·CH$_2$Cl$_2$ **4c**·2 CH$_2$Cl$_2$

Crystal data: $C_{47}H_{47}AgAuNO_2$, M = 1047.62. Monoclinic, space group $P2_1/n$ (as no. 14), a = 11.5412(3), b = 19.7886(4), c = 19.5343(4) Å, β = 105.047(2) °, V = 4308.36(17) $Å^3$. Z = 4, Dc = 1.615 g cm^{-3}, F(000) = 2080, T = 140(2) K, μ(Mo-Kα) = 40.19 cm^{-1}, λ(Mo-Kα) = 0.71073 Å.

The crystal was a yellow block. From a sample under oil, one, *ca* 0.05 x 0.10 x 0.10 mm, was mounted on a small loop and fixed in the cold nitrogen stream on a Rigaku Oxford Diffraction Xcalibur diffractometer, equipped with Mo-Kα radiation, Sapphire detector and graphite monochromator. Intensity data were measured by thin-slice ω-scans. Total no. of reflections recorded, to θ_{max} = 29.4°, was 21,591 of which 9955 were unique (Rint = 0.032); 8411 were 'observed' with $I > 2\sigma_I$.

Data were processed using the CrysAlisPro-CCD and -RED (S1) programs. The structure was determined by the intrinsic phasing routines in the SHELXT program (S2) and refined by full-matrix least-squares methods, on F^2's, in SHELXL (S2). There is solvent, CH_2Cl_2, included in the lattice, disordered over two orientations. The non-hydrogen atoms were refined with anisotropic thermal parameters. The hydrogen atoms were included in idealised positions and their Uiso values were set to ride on the Ueq values of the parent carbon atoms. At the conclusion of the refinement, wR_2 = 0.056 and R_1 = 0.039 (S2) for all 9955 reflections weighted $w = [\sigma^2(F_o^2) + (0.0188\ P)^2]^{-1}$ with $P = (F_o^2 + 2F_c^2)/3$; for the 'observed' data only, R_1 = 0.029.

In the final difference map, the highest peak (*ca* 1.0 e$Å^{-3}$) was near Au(1).

Scattering factors for neutral atoms were taken from reference (S5). Computer programs used in this analysis have been noted above, and were run through WinGX (S4) on a Dell Optiplex 780 PC at the University of East Anglia.

Crystal structure analysis of [{(C^C)Au(C≡$C^tBu)_2$}{Cu(C≡NXyl)}] **5a**

Crystal data: $C_{41}H_{51}AuCuN$, 0.064(Au Cu), M = 835.26. Monoclinic, space group $P2_1/n$ (equiv. to no. 14), a = 12.4101(6), b = 15.6179(10), c = 19.0101(16) Å, V = 3684.5 (4) $Å^3$. Z = 4, Dc = 1.506 g cm^{-3}, F(000) = 1676, T = 140(1) K, μ(Mo-Kα) = 48.76 cm^{-1}, λ(Mo-Kα) = 0.71073 Å.

The crystal was a colourless block. From a sample under oil, one, *ca* 0.20 x 0.20 x 0.20 mm, was mounted on a small loop and fixed in the cold nitrogen stream on a Rigaku Oxford Diffraction Xcalibur diffractometer, equipped with Mo-Kα radiation, Sapphire-3 detector and graphite monochromator. Intensity data were measured by thin-slice ω-scans. Total no. of

reflections recorded, to θ_{max} = 27.5°, was 12,580 of which 6737 were unique (Rint = 0.030); 5677 were 'observed' with I > $2\sigma_I$.

Data were processed using the CrysAlisPro-CCD and -RED (S1) programs. The structure was determined by the intrinsic phasing routines in the SHELXT program (S2) and refined by full-matrix least-squares methods, on F^2's, in SHELXL (S2). The ^{t}Bu group of C(17) was found to be disordered in two orientations with occupation ratio of 0.832 : 0.168. There are two difference peaks parallel to the Au(1)…Cu(1) vector, and very similar in distance apart; these were included as Au(2) and Cu(2) in the refinement process with an occupation factor which refined to 0.064; no further coordinated atoms/peaks were observed. The non-hydrogen atoms (except the minor components of the disordered ^{t}Bu group) were refined with anisotropic thermal parameters. Hydrogen atoms were included in idealised positions and their Uiso values were set to ride on the Ueq values of the parent carbon atoms. At the conclusion of the refinement, wR_2 = 0.080 and R_1 = 0.038 (2B) for all 6737 reflections weighted w = $[\sigma^2(F_o^2) + (0.0385\ P)^2 + 0.4868\ P]^{-1}$ with $P = (F_o^2 + 2F_c^2)/3$; for the 'observed' data only, R_1 = 0.030.

In the final difference map, the highest peak (*ca* 0.86 $eÅ^{-3}$) was near Au(1).

Scattering factors for neutral atoms were taken from reference (S5). Computer programs used in this analysis have been noted above, and were run through WinGX (S4) on a Dell Optiplex 780 PC at the University of East Anglia.

Only for connectivity. [{(C^C)Au(C≡CC_6H_4OMe-4)$_2$}{Cu(C≡NXyl)}] **5c** Colourless block (0.1×0.1×0.05) grown by layering with petrol a solution of the solid in CD_2Cl_2. The symmetry space group of the crystal is $P2_1/n$. A SHEL 0.8 999 instruction was used. EADP was used in some carbon atoms. DFIX was used for modelling the OMe substituents. Many Q peaks larger than 1 with no chemical meaning were found, what causes several A alerts in the checkcif. Besides, the poor Thermal parameters of several atoms cause other A alerts in the checkcif. Due to the poor quality and the unfruitful attempts of growing higher quality crystals, this structure is provided only for connectivity purpose.

**Table S1.** Selected crystal data and structure refinement details for:					
	**1**	**2**	**4a**	**4c$_2$**·CH_2Cl_2	**5a**
Empirical formula	C32 H42 Ag Au	C32 H42 Au Cu	C41 H51 Ag Au N	C47 H47 Ag Au N O2, C H2 Cl2	C41 H51 Au Cu N, 0.064(Au Cu)
F_w	731.49	687.16	862.66	1047.62	835.26
T (K)	140(2)	140(2)	105.1(6)	140(2)	140(2)
Crystal system, space group	Monoclinic, P2$_1$/n	Monoclinic, P2$_1$/n	Monoclinic, P2$_1$/c	Monoclinic, P2$_1$/n	Monoclinic, P2$_1$/n
a(Å)	14.9787(3)	14.8997(3)	28.2944(5)	11.5412(3)	12.4101(6)
b(Å)	10.8556(2)	10.57203(15)	13.2307(2)	19.7886(4)	15.6179(10)
c(Å)	19.7012(5)	20.0210(4)	21.4762(3)	19.5343(4)	19.0101(16)
α(deg)	90	90	90	90	90
β(deg)	110.724(3)	110.542(2)	111.396(2)	105.047(2)	90
γ(deg)	90	90	90	90	90
Volume (A^3)	2996.19(12)	2953.17(10)	7485.6(2)	4308.36(17)	3684.5(4)
Z	4	4	8	4	4
D_{calcd} (Mg/m^3)	1.622	1.546	1.531	1.615	1.506
Absorption coefft (mm^{-1})	5.561	5.699	4.465	4.019	4.876
F(000)	1440	1368	3440	2080	1676
Crystal colour, shape	Light yellow block	Colourless block	Colourless plate	Yellow block	Colourless block
Crystal size, (mm)	0.10 x 0.10 x 0.20	0.10 x 0.10 x 0.15	0.05 x 0.15 x 0.20	005 x 0.10 x 0.10	0.02 x 0.02 x 0.02
θ range for data collection (º)	3.578 to 27.499	3.537 to 27.482	3.604 to 27.484	2.984 to 29.37	3.533 to 27.495
No. of 'observed' reflns, Rint	6301, 0.041	6507, 0.049	15244, 0.043	8411, 0.032	5677, 0.030
Data // restraints // params	6867 // 0 // 335	6772 // 0 // 307	17129 // 0 // 793	9955 // 0 // 516	6737 // 0 // 435
Goodness-of-fit on F^2	1.092	1.142	1.037	1.016	1.027
Final R indexes [I>2σ(I)]	R_1 = 0.0198, wR_2 = 0.0414	R_1 = 0.0212, wR_2 = 0.0467	R_1 = 0.0312, wR_2 = 0.0672	R_1 = 0.0286, wR_2 = 0.0529	R_1 = 0.0300, wR_2 = 0.0763
R indexes (all data)	R_1 = 0.0237, wR_2 = 0.0424	R_1 = 0.0226, wR_2 = 0.0471	R_1 = 0.0379, wR_2 = 0.0696	R_1 = 0.0391, wR_2 = 0.0558	R_1 = 0.0385, wR_2 = 0.0805
Largest diff peak and hole (e.Å^{-3})	0.866 and -0.474	1.257 and -1.006	1.557 and -1.865	1.039 and -0.757	0.857 and -1.155

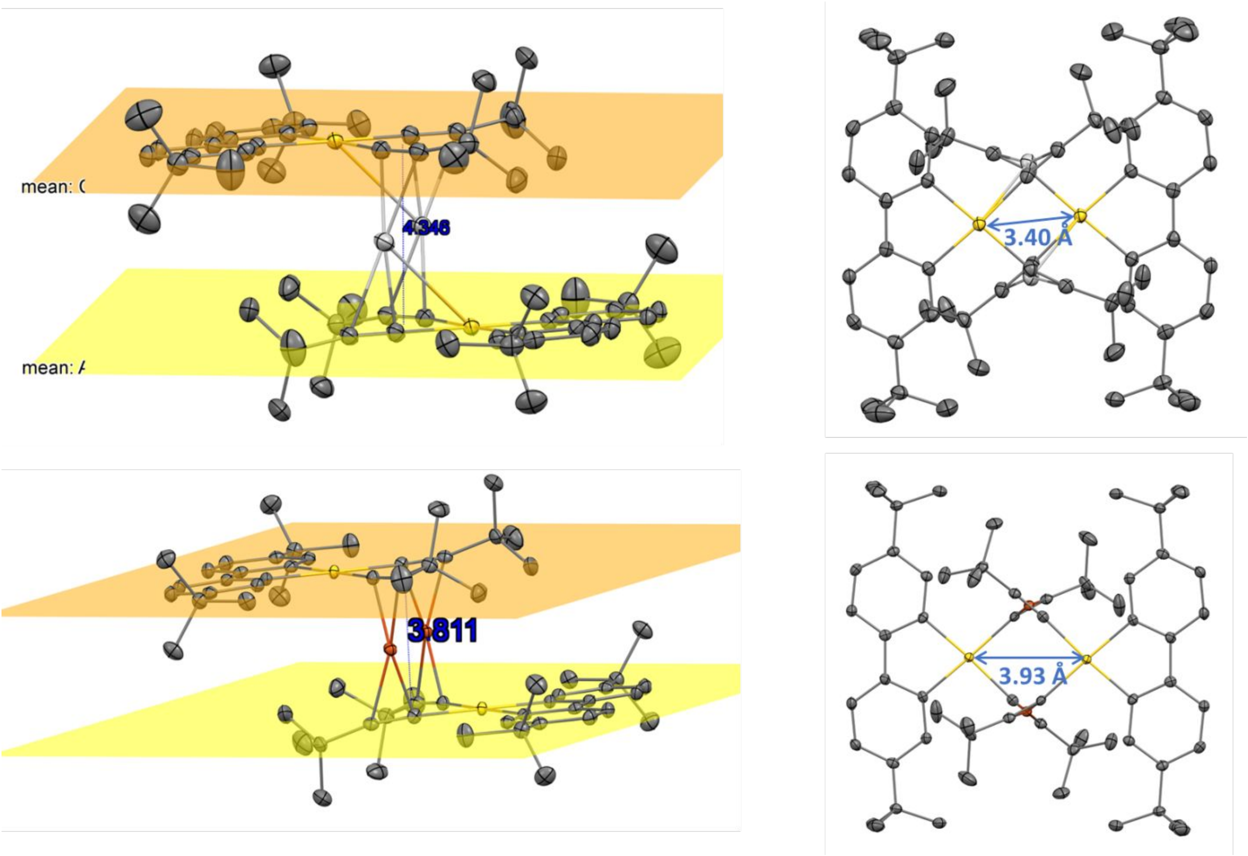

Figure S12. Views of the relative disposition of the $\{(C\text{^}C)Au(C{\equiv}C^tBu)_2\}$ fragments in $[\{(C\text{^}C)Au(C{\equiv}C^tBu)_2\}_2Ag_2]$ **1** (top) and $[\{(C\text{^}C)Au(C{\equiv}C^tBu)_2\}_2Cu_2]$ **2** (down). This Figure supports the section **Results and Discussion**, *X-Ray diffraction studies* in the Manuscript.

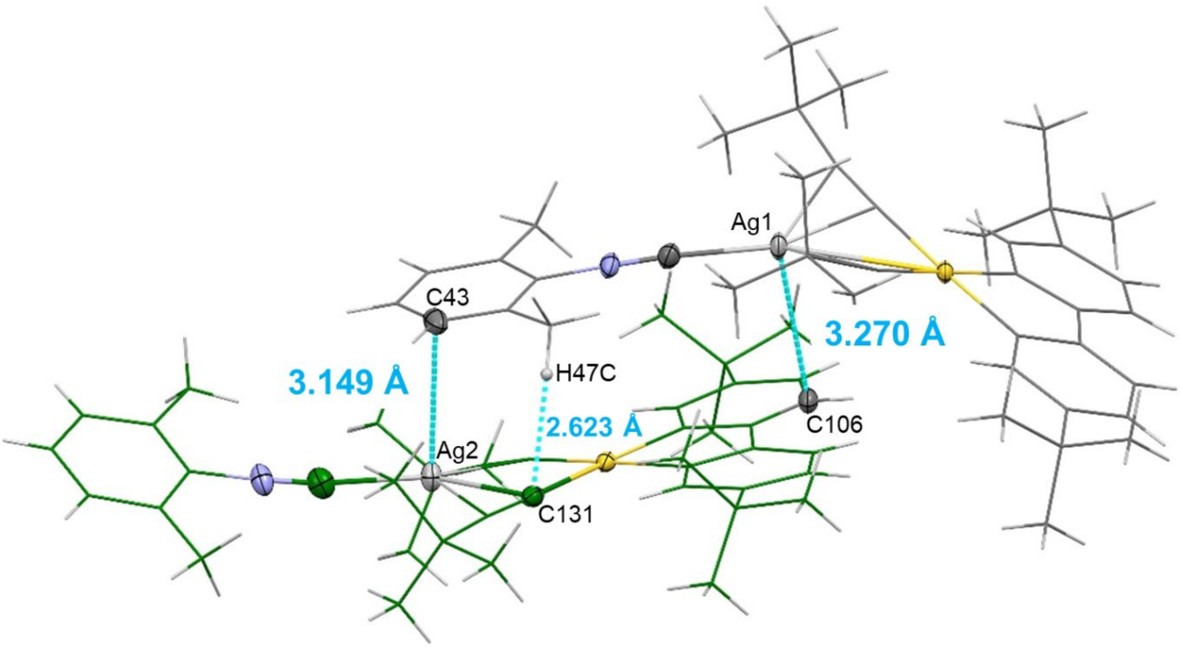

Figure S13. Detail of the crystal packing of [{(C^C)Au(C≡CtBu)2}{Ag(C≡NXyl)}] **4a** showing the intermolecular interactions between both types of molecules in the lattice. This Figure supports the section **Results and Discussion**, *X-Ray diffraction studies* in the Manuscript.

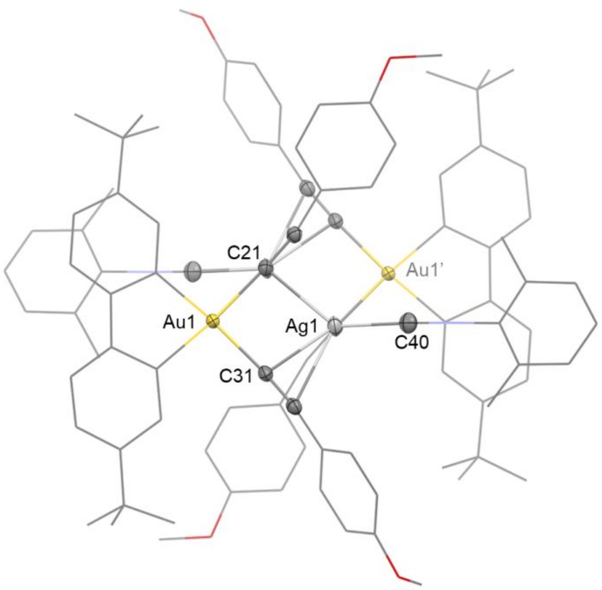

Figure S14. Top-to-down view of the X-ray structure of [{(C^C)Au(C≡CC$_6$H$_4$OMe-4)$_2$}{Ag(C≡NXyl)}]$_2$·CH$_2$Cl$_2$ **4c$_2$**·CH$_2$Cl$_2$ **4c**·2 CH$_2$Cl$_2$. This Figure supports the section **Results and Discussion**, *X-Ray diffraction studies* in the Manuscript.

[{(C^C)Au(C≡CC$_6$H$_4$OMe-4)$_2$}{Cu(C≡NXyl)}] **5c**

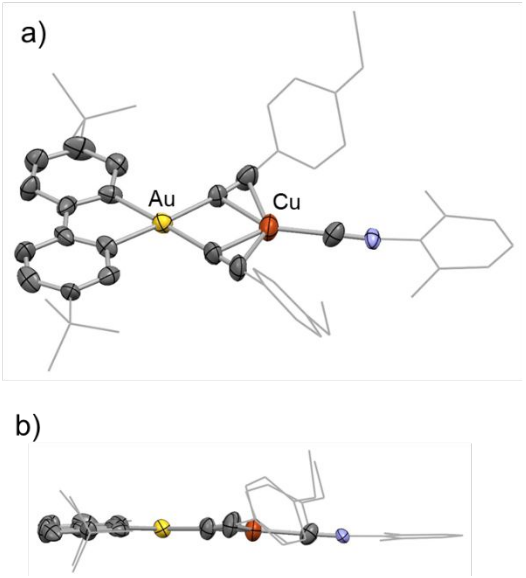

Figure S15. Connectivity of the X-ray structure of [{(C^C)Au(C≡CC$_6$H$_4$OMe-4)$_2$}{Cu(C≡NXyl)}] **5c** showing **(a)** a general view **(b)** a side view that highlights the "in plane" disposition of the Cu(C≡NXyl) fragment.

S3. Photophysical Properties

UV−visible absorption spectra were recorded using a Perkin-Elmer Lambda 35 UV/vis spectrometer. Excitation and emission spectra were measured using a (TCSPC) FluoroLog Horiba Jobin Yvon spectrofluorometer and/or Edimburg FLS 1000 spectrofluorimeters. Lifetime measurements were performed with Edimburg FLS 1000 spectrofluorimeter with μF2 pulse lamp (Power: 100 W, Fuse: 3.15 Amp A/S); the estimated uncertainty is ~10% or better. Quantum Yields were measured with Hamamatsu Absolute PL Quantum Yield Spectrometer; the estimated uncertainty is ~5% or better. Polymeric sample preparation: PMMA (polymethyl methacrylate) was purchased from commercial sources. Thin films were prepared by spin/drop casting from a solution of the corresponding complex and the amount of polymer to reach the desired concentration in each case. Films were then placed under vacuum for 10 minutes to remove residual solvent.

Table S2: Absorption data for the complexes in CH_2Cl_2 (5E^{-5} *M*)

Complex	Absorbance [nm] (10^3 ε/M^{-1} cm^{-1}).
**1**	291 (13.0), 310 (7.3), 322 (9.0), 355 (1.3)
**2**	291 (51.4), 308 (24.4), 322 (25.9), 354 (5.4)
**3a**	291 (38.1), 308 (15.6), 320 (16.6), 355 (3.1)
**3b**	307 (33.5), 318sh (27.1), 359sh (2.4)
**3c**	308 (17.1), 319 (17.0), 332sh (8.0), 364sh (1.4)
**4a**	287 (29.6), 307 (25.6), 319 (28.9), 341 (6.9)
**4b**	291 (44.1), 306 (13.1), 319 (10.3), 351sh (2.0)
**4c**	293 (43.4), 296 (40.7), 318a (14.6), tail to 370 nm.
**5a**	306 (36.9), 319 (35.9), 342sh (8.7)
**5b**	319 (21.1), 350sh (3.5)
**5c**	318sh (29.1), 342sh (8.7), tail to 365 nm.

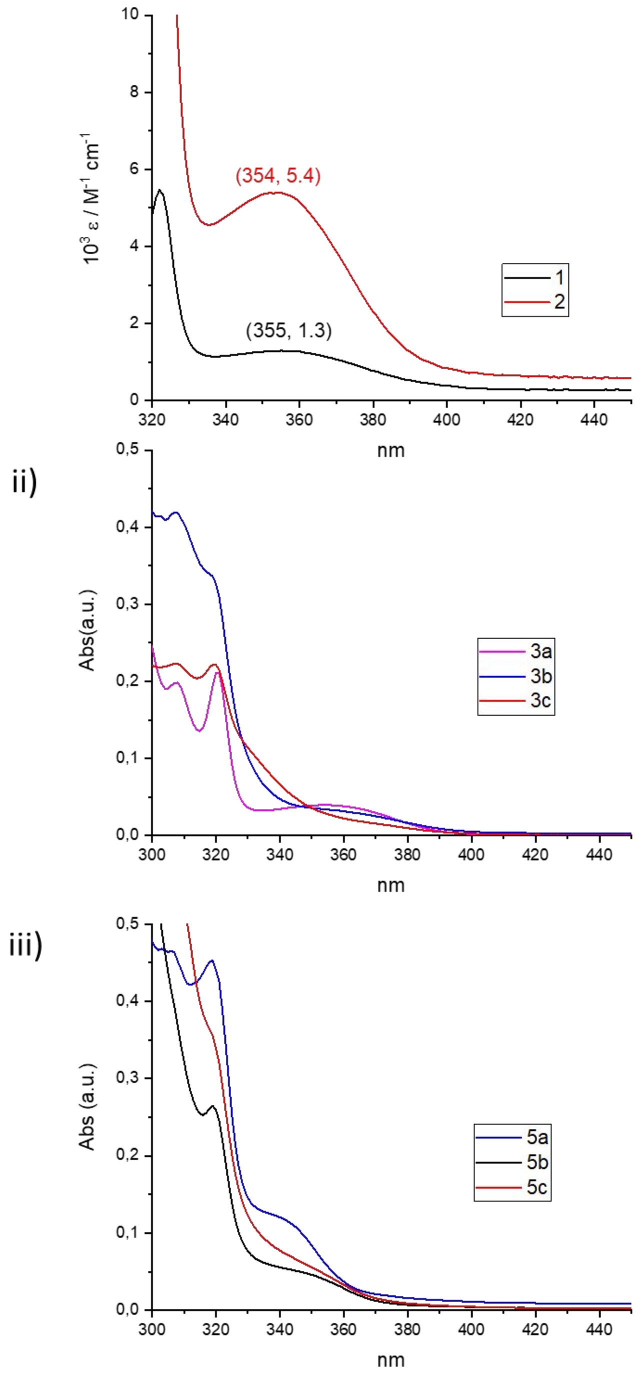

Figure S16. UV-Vis Absorption Spectra in CH_2Cl_2 5 × 10^{-5} M of complexes **1** and **2** (i) **3a**, **3b** and **3c** (ii), **5a**, **5b** and **5c** (iii). This Figure Supports the section **Photophysical Properties**. *Absorption Spectra*.

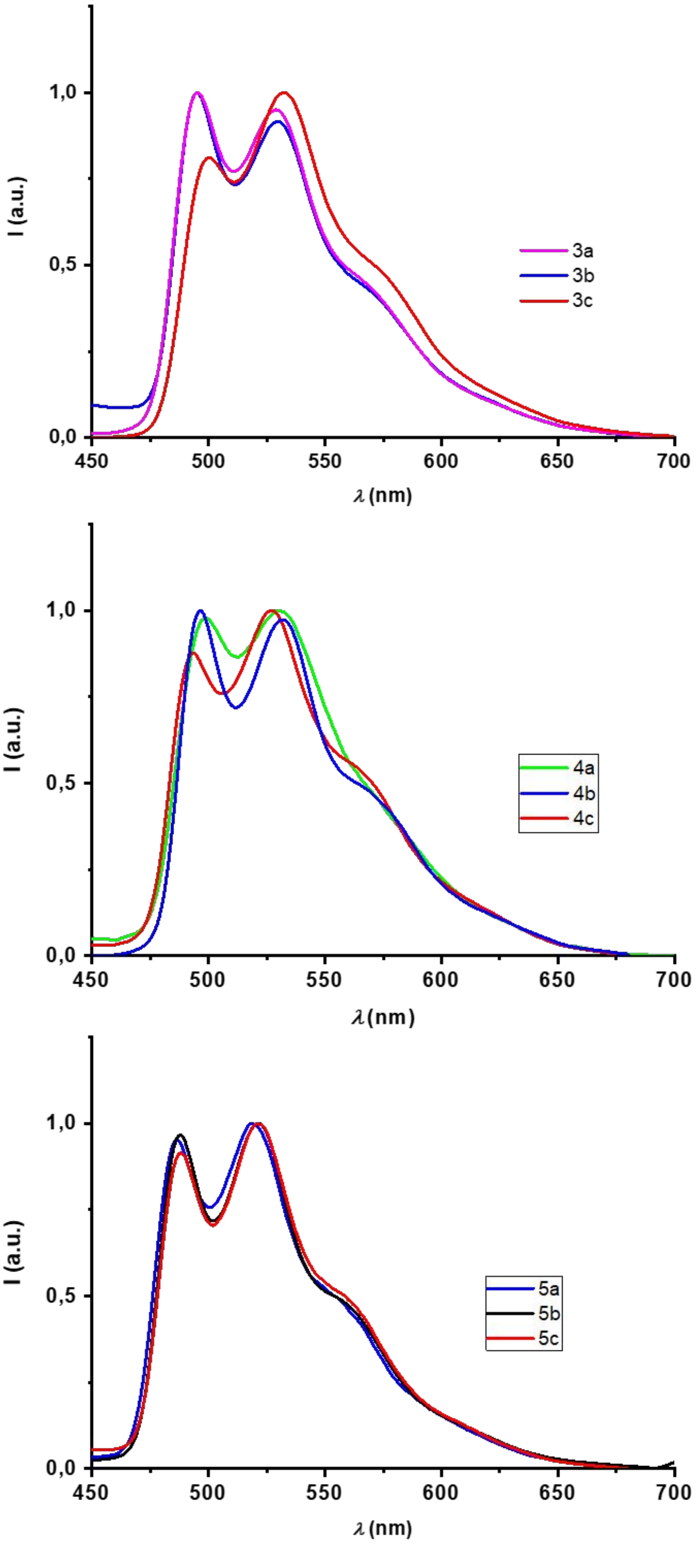

Figure S17: PL spectra of complexes **3(a,b,c)** top, **4(a,b,c)** (middle) and **5(a,b,c)** (down) in PMMA (10%). This Figure Supports the section **Photophysical Properties**. *Photoluminescence Spectra.*

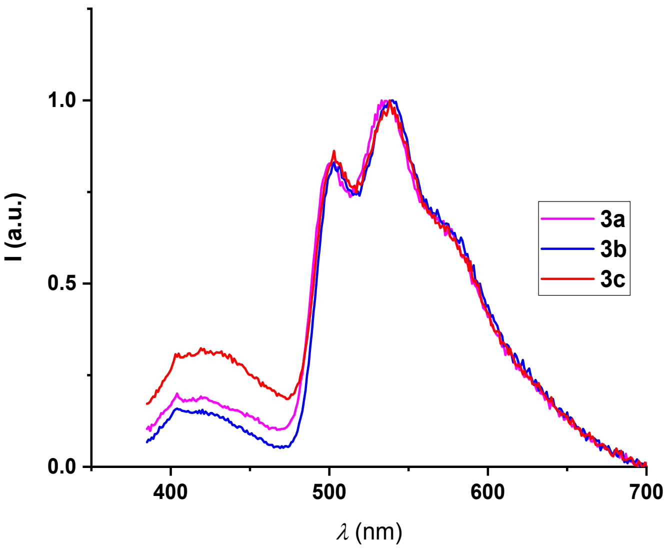

Figure S18: PL spectra of complexes **3(a,b,c)** in in CH_2Cl_2 1 × 10^{-3} M. This Figure Supports the section **Photophysical Properties**. *Photoluminescence Spectra.*

S4. Theoretical Calculations

Calculations were carried out with the Gaussian 16 package1, using GaussView 6 to visualize the results. Overlap populations between molecular fragments were calculated using the GaussSum 3.0 software.[S6] Three functionals were studied B3LYP,[S7] CAM-B3LYP[S8] and ωB97X-D[S9], being the range-separated and dispersion-corrected hybrid density functional ωB97X-D the one to better predict the emission energies. The basis set used for the metal atoms was the LanL2DZ effective core potential and 6-31G(d,p) for the ligand atoms.[S10] No negative frequency was found in the vibrational frequency analysis of the final equilibrium geometries. The effect of the solvent in the ground state calculations (DFT, TD-DFT) was taken into account using the polarized continuum model approach (PCM)[S11] implemented in the Gaussian 16 software while the T1 state calculations were performed without any solvent. The emission energies were calculated as the difference between the optimized T1 state and the S0 state in the optimized T1 geometry (adiabatic electronic transition).[S12]

Table S3. DFT optimized geometries for ground state (in CH2Cl2) and triplet state (in gas phase) for **1**.

	X-ray	S_0 (CH_2Cl_2)	T_1 (gas)
	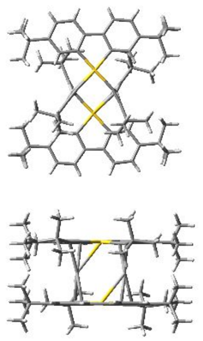	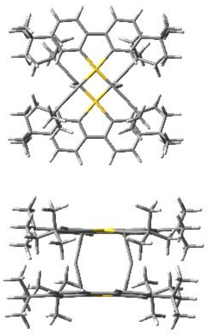	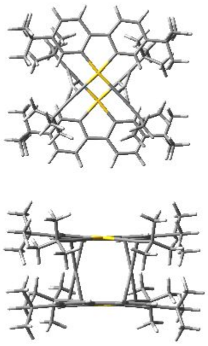
Au-C1	2.047-2.045	2.051-2.051	2.050-2.030
Au-C2	2.042-2.042	2.051-2.051	2.049-2.029
Au-C3	2.056-2.056	2.076-2.076	2.078-2.076
Au-C5	2.065-2.066	2.077-2.078	2.076-2.075
Au-Ag1	3.215-3.216	3.237-3.237	3.224-3.175
Au-Ag2	3.298-3.299	3.250-3.250	3.225-3.177
Au-Au	5.519	5.128	5.079
Ag1-C3	2.214-2.215	2.296-2.295	2.285-2.280
Ag1-C4	2.393-2.394	2.507-2.506	2.485-2.502
Ag2-C5	2.201-2.202	2.296-2.296	2.285-2.280
Ag2-C6	2.378-2.379	2.500-2.500	2.483-2.500
Ag-Ag	3.461	3.973	3.894
C3-C4	1.215-1.216	1.231-1.230	1.230-1.230
C5-C6	1.209-1.209	1.231-1.231	1.230-1.230
C1-Au-C2	81.54-81.58	80.91-80.96	81.00-80.97
C3-Au-C5	86.71-86.67	90.60-90.61	90.84-90.54
Au-C3-C4	174.98-175.06	178.65-178.62	179.03-178.08
Au-C5-C6	164.17-164.17	178.97-178.94	179.06-178.06

Table S3 Continued. DFT optimized geometries for ground state (in CH2Cl2) and triplet state (in gas phase) for **2**.

	X-ray	S_0 (CH_2Cl_2)	T_1 (gas)
	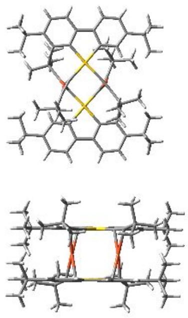	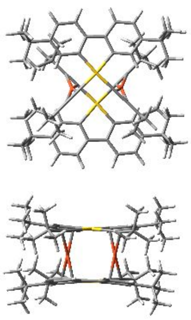	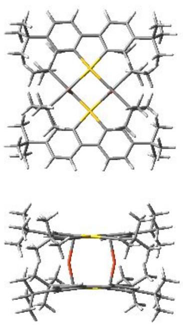
Au-C1	2.041-2.042	2.050-2.049	2.029-2.049
Au-C2	2.043-2.043	2.050-2.050	2.029-2.049
Au-C3	2.0532.053	2.077-2.076	2.073-2.076
Au-C5	2.059-2.058	2.076-2.077	2.073-2.076
Au-Cu1	3.232-3.232	3.112-3.112	3.053-3.113
Au-Cu2	3.296-3.295	3.113-3.113	3.053-3.114
Au-Au	5.471	4.941	4.897
Cu1-C3	2.000-1.999	2.035-2.035	2.032-2.040
Cu1-C4	2.1082.108	2.214-2.214	2.216-2.206
Cu2-C5	1.996-1.996	2.034-2.034	2.032-2.040
Cu2-C6	2.103-2.103	2.214-2.214	2.216-2.205
Cu-Cu	3.561	3.786	3.747
C3-C4	1.229-1.229	1.233-1.233	1.233-1.233
C5-C6	1.224-1.224	1.233-1.233	1.233-1.233
C1-Au-C2	81.25-81.25	81.08-81.09	81.03-81.07
C3-Au-C5	86.47-86.45	91.02-91.00	90.81-91.36
Au-C3-C4	174.13-174.13	179.59-179.63	177.55-178.75
Au-C5-C6	165.38-165.40	179.78-179.76	177.52-178.72

Table S3 Continued. DFT optimized geometries for ground state (in CH2Cl2) and triplet state (in gas phase) for **3a**.

	X-ray	S_0 (CH_2Cl_2)	T_1 (gas)
		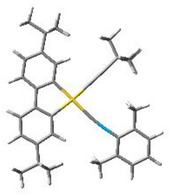	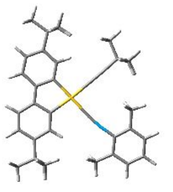
Au-C1		2.031	2.009
Au-C2		2.061	2.044
Au-C3		2.055	2.053
Au-C5		2.081	2.064
C3-C4		1.217	1.216
C5-N1		1.160	1.163
C1-Au-C2		81.05	81.01
C3-Au-C5		87.35	87.21
Au-C3-C4		179.91	179.68
Au-C5-N1		173.61	173.89
Angle between the CNXyl and the Au plane		11.36	8.41

Table S3 Continued. DFT optimized geometries for ground state (in CH2Cl2) and triplet state (in gas phase) for **4c**.

	X-ray	S_0 (CH_2Cl_2)	T_1 (gas)
	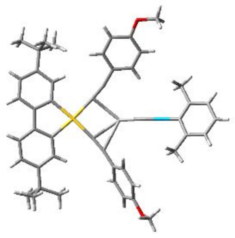	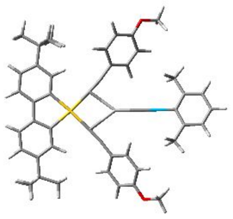	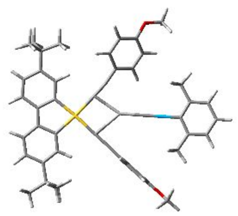
Au-C1	2.052	2.050	2.029
Au-C2	2.045	2.051	2.030
Au-C3	2.072	2.078	2.090
Au-C5	2.052	2.078	2.089
Au-Ag	3.385	3.436	3.399
C3-C4	1.210	1.230	1.233
C5-C6	1.216	1.229	1.230
Ag-C3	2.506	2.453	2.377
Ag-C4	2.769	2.632	2.522
Ag-C5	2.389	2.461	2.463
Ag-C6	2.620	2.638	2.610
Ag-C7	2.112	2.129	2.118
C7-N1	1.149	1.163	1.163
C1-Au-C2	80.82	80.95	80.96
C3-Au-C5	90.89	90.12	89.78
Au-C3-C4	168.61	176.69	178.54
Au-C5-C6	172.03	176.69	178.65
Au-Ag-C7	155.01	179.39	172.87
Ag-C7-N1	176.45	179.53	179.12
Angle between the Ag and the Au plane	7.18	0.30	1.21

Angle between the PhOMe1 plane and the Au plane	20.51	34.42	31.24
Angle between the PhOMe2 plane and the Au plane	65.60	38.44	89.83
Angle between the Xyl plane and the Au plane	19.82	14.44	3.51

Table S3 Continued. DFT optimized geometries for ground state (in CH2Cl2) and triplet state (in gas phase) for **5a**.

	X-ray	S_0 (CH_2Cl_2)	T_1 (gas)
	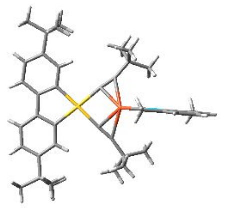	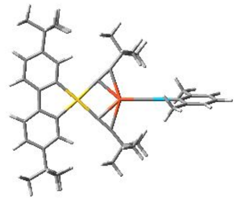	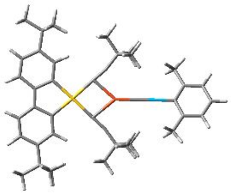
Au-C1	2.037	2.046	2.026
Au-C2	2.044	2.045	2.027
Au-C3	2.053	2.077	2.079
Au-C5	2.050	2.076	2.079
Au-Cu	3.083	3.169	3.184
C3-C4	1.204	1.231	1.230
C5-C6	1.208	1.230	1.229
Cu-C3	2.134	2.171	2.181
Cu-C4	2.280	2.347	2.349
Cu-C5	2.137	2.180	2.181
Cu-C6	2.312	2.357	2.348
Cu-C7	1.878	1.926	1.934
C7-N1	1.171	1.165	1.168
C1-Au-C2	81.13	81.11	81.07
C3-Au-C5	84.69	85.88	85.71
Au-C3-C4	173.93	178.70	178.64
Au-C5-C6	175.39	178.64	178.60
Au-Cu-C7	160.07	176.39	179.95
Cu-C7-N1	175.56	179.40	179.97
Angle between the Cu and the Au plane	11.81	2.80	0.00
Angle between the Xyl plane and the Au plane	86.09	77.08	24.96

Table S3 Continued. DFT optimized geometries for ground state (in CH2Cl2) and triplet state (in gas phase) for **5c**.

	X-ray	S_0 (CH_2Cl_2)	T_1 (gas)
	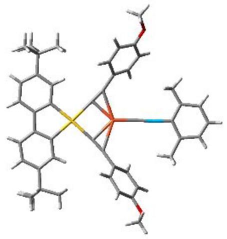	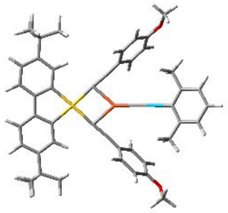	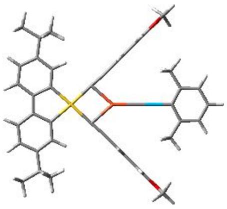
Au-C1	2.066	2.046	2.026
Au-C2	2.072	2.046	2.027
Au-C3	2.129	2.086	2.090
Au-C5	2.131	2.086	2.090
Au-Cu	3.107	3.148	3.150
C3-C4	1.149	1.230	1.231
C5-C6	1.150	1.231	1.231
Cu-C3	2.135	2.176	2.166
Cu-C4	2.371	2.340	2.336
Cu-C5	2.147	2.174	2.166
Cu-C6	2.460	2.341	2.336
Cu-C7	1.908	1.921	1.922
C7-N1	1.143	1.164	1.166
C1-Au-C2	80.70	81.02	80.99
C3-Au-C5	86.89	86.26	86.37
Au-C3-C4	173.64	176.87	177.40
Au-C5-C6	173.43	177.11	177.40
Au-Cu-C7	176.43	170.11	180.00
Cu-C7-N1	177.24	178.03	180.00
Angle between the Cu and the Au plane	1.07	6.10	0.02

Angle between the PhOMe1 plane and the Au plane	50.19	65.91	89.32
Angle between the PhOMe2 plane and the Au plane	58.47	61.42	89.27
Angle between the Xyl plane and the Au plane	2.66	19.42	3.89

Table S4. Composition (%) of Frontier MOs in terms of ligands and metals in the ground state in CH_2Cl_2.

1

MO	eV	C^C 1	C≡C^{t}Bu 1	Au 1	C^C 2	C≡C^{t}Bu 2	Au 2	Ag 1	Ag 2
LUMO+5	1.46	11	5	0	11	5	0	35	35
LUMO+4	1.41	42	2	6	43	2	6	0	0
LUMO+3	1.28	12	6	12	12	7	12	20	20
LUMO+2	1.09	32	3	1	32	3	1	14	14
LUMO+1	0.42	28	5	12	28	5	12	4	4
LUMO	0.29	18	11	15	18	11	15	7	7
HOMO	-7.40	54	0	1	44	0	1	0	0
HOMO-1	-7.40	44	0	1	54	0	1	0	0
HOMO-2	-8.41	35	7	4	35	7	4	5	5
HOMO-3	-8.51	45	2	1	45	2	1	1	1
HOMO-4	-8.54	47	1	2	47	1	2	0	0
HOMO-5	-8.65	41	5	4	41	5	4	1	1

2

MO	eV	C^C 1	C≡C^{t}Bu 1	Au 1	C^C 2	C≡C^{t}Bu 2	Au 2	Cu 1	Cu 2
LUMO+5	1.50	21	10	15	21	10	15	4	4
LUMO+4	1.41	20	8	18	20	8	18	4	4
LUMO+3	1.35	43	2	5	43	2	5	0	0
LUMO+2	1.12	42	3	1	42	3	1	4	4
LUMO+1	0.29	24	8	13	24	8	13	6	6
LUMO	0.27	17	12	14	17	12	14	7	7
HOMO	-7.41	51	0	1	47	0	1	0	0
HOMO-1	-7.42	47	0	1	51	0	1	0	0
HOMO-2	-8.49	35	5	3	35	5	3	6	6
HOMO-3	-8.55	50	1	1	44	1	1	1	1
HOMO-4	-8.55	44	1	2	50	1	2	0	0
HOMO-5	-8.69	42	3	4	42	3	4	1	1

3a

MO	eV	C^C	C≡NXyl	C≡C^{t}Bu	Au
LUMO+5	1.93	15	57	8	19
LUMO+4	1.46	2	98	0	0
LUMO+3	1.41	71	13	2	15
LUMO+2	1.19	39	35	3	23
LUMO+1	0.87	63	25	1	11
LUMO	-0.11	12	71	2	15
HOMO	-7.43	99	0	0	1
HOMO-1	-8.35	34	0	59	7
HOMO-2	-8.44	4	2	89	4
HOMO-3	-8.53	94	1	2	3
HOMO-4	-8.90	50	19	27	4
HOMO-5	-9.10	0	99	1	0

4c

MO	eV	C^C	C≡CPhOMe	Au	Ag	C≡NXyl
LUMO+5	1.45	0	5	0	0	95
LUMO+4	1.33	53	25	0	11	12
LUMO+3	1.20	13	38	1	12	36
LUMO+2	1.19	2	79	1	9	8
LUMO+1	0.56	36	36	25	1	3
LUMO	0.11	0	2	0	10	88
HOMO	-7.24	97	2	2	0	0
HOMO-1	-7.61	5	90	2	4	0
HOMO-2	-7.69	4	93	2	2	0
HOMO-3	-8.36	96	1	3	0	0
HOMO-4	-8.50	76	16	6	2	0
HOMO-5	-8.85	39	41	8	10	3

5a

MO	eV	C^C	C≡C^{t}Bu	Au	Cu	C≡NXyl
LUMO+5	1.88	40	16	33	9	2
LUMO+4	1.48	0	0	0	0	100
LUMO+3	1.48	83	5	11	0	1
LUMO+2	0.99	5	3	0	35	58
LUMO+1	0.78	58	11	27	2	2
LUMO	0.19	0	2	0	5	93
HOMO	-7.27	98	0	1	0	0
HOMO-1	-8.33	92	3	5	0	0
HOMO-2	-8.41	74	15	8	3	0
HOMO-3	-8.48	7	47	0	26	19
HOMO-4	-8.55	27	36	6	26	5
HOMO-5	-8.80	6	77	3	14	1

5c

MO	eV	C^C	C≡CPhOMe	Au	Cu	C≡NXyl
LUMO+5	1.53	1	71	0	11	18
LUMO+4	1.46	2	39	1	11	47
LUMO+3	1.32	3	80	2	4	10
LUMO+2	1.03	29	60	4	5	1
LUMO+1	0.58	39	34	24	2	1
LUMO	0.15	0	3	0	12	85
HOMO	-7.26	97	1	2	0	0
HOMO-1	-7.57	2	89	0	9	1
HOMO-2	-7.93	6	85	3	5	1
HOMO-3	-8.40	95	1	4	0	0
HOMO-4	-8.52	80	12	7	1	1
HOMO-5	-8.80	25	35	4	31	5

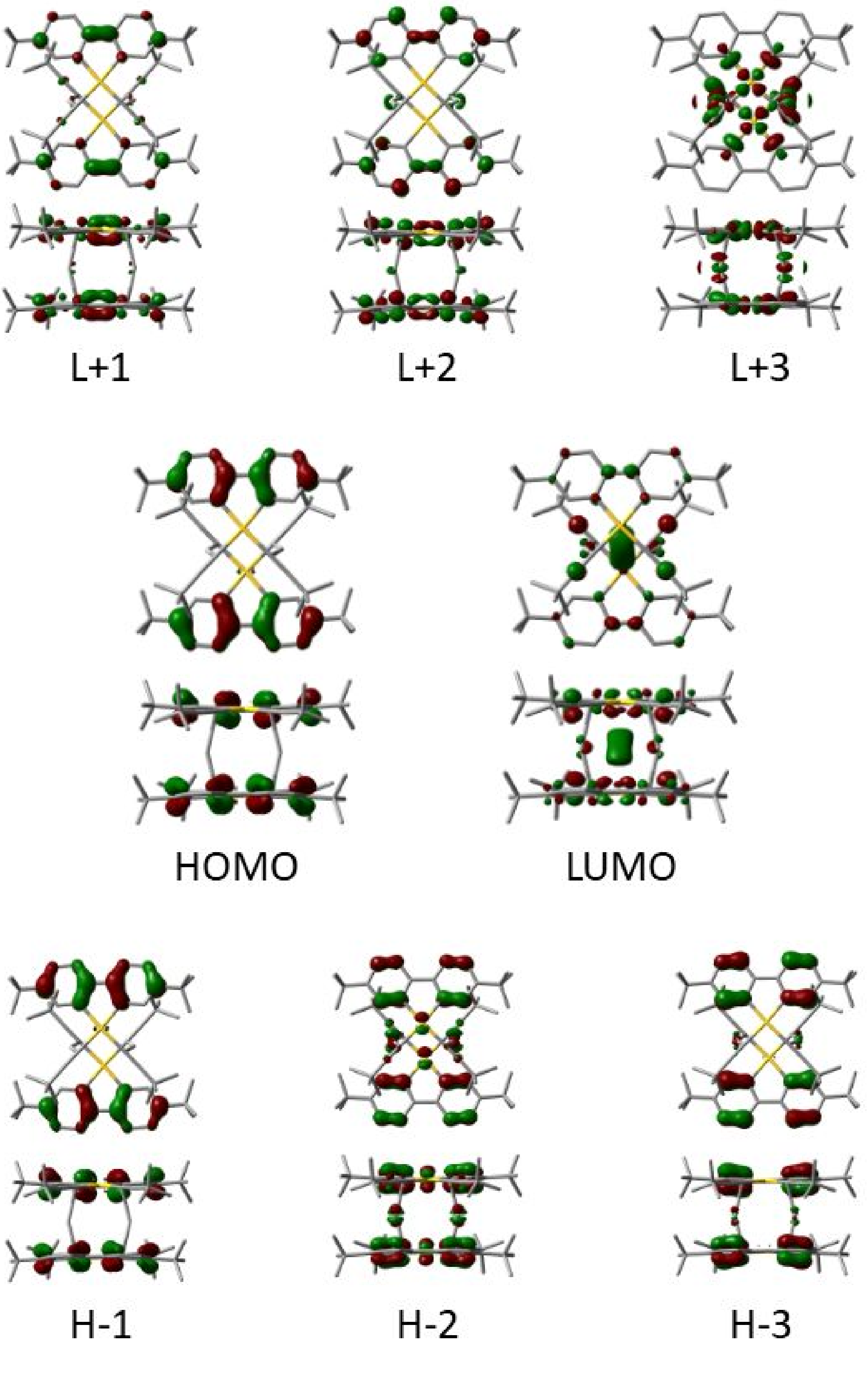

Figure S19. Selected frontier Molecular Orbitals for **1** in the ground state.

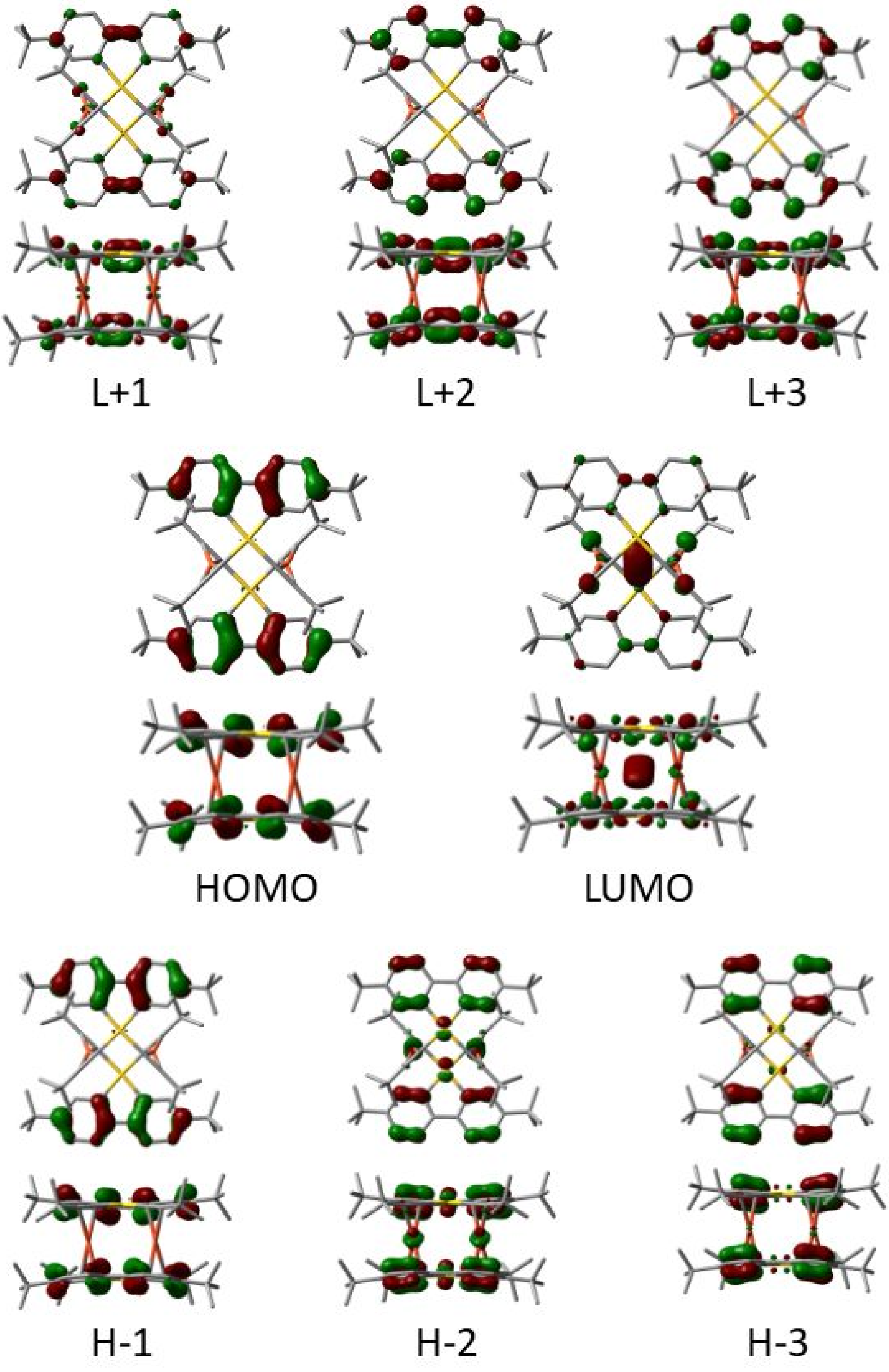

Figure S20. Selected frontier Molecular Orbitals for **2** in the ground state.

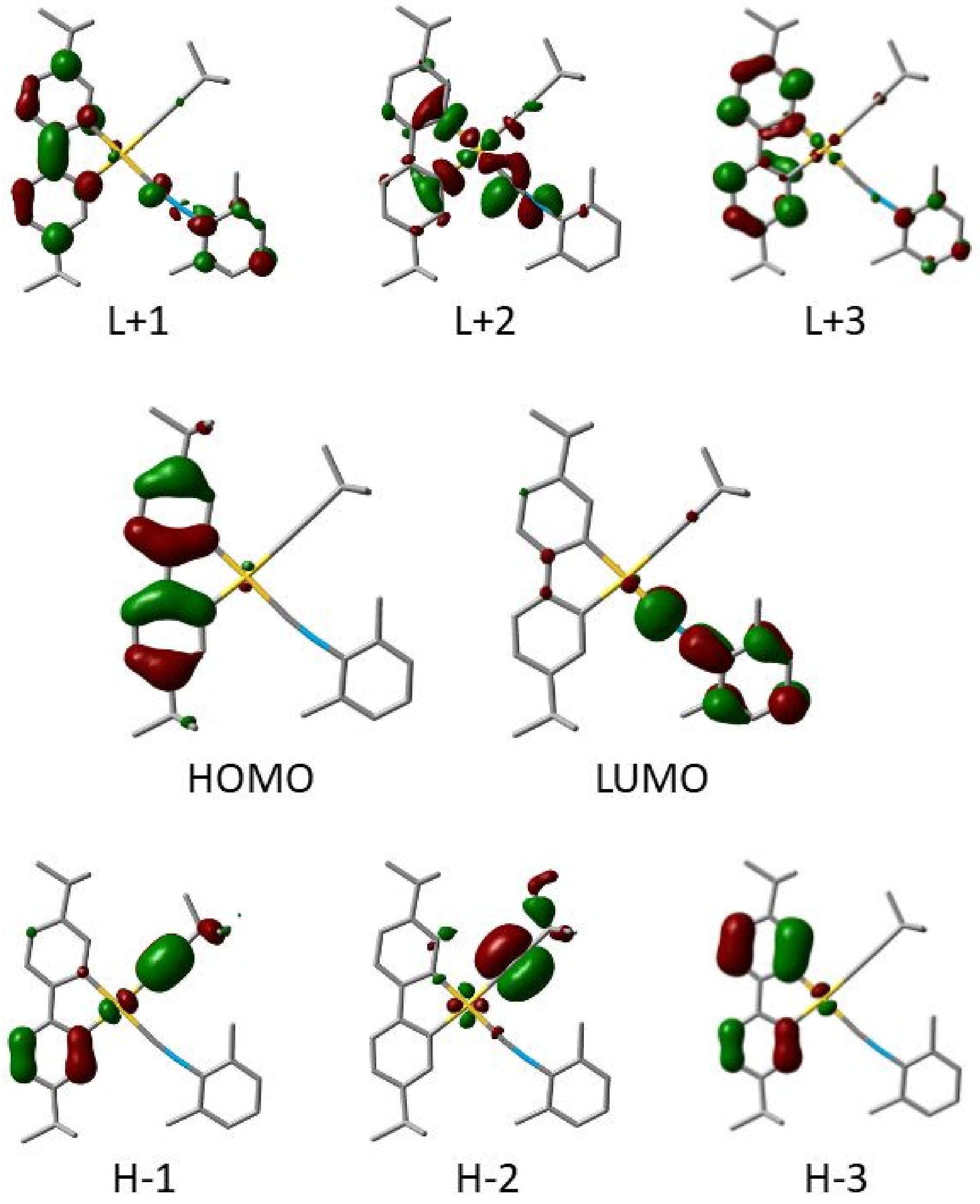

Figure S21. Selected frontier Molecular Orbitals for **3a** in the ground state.

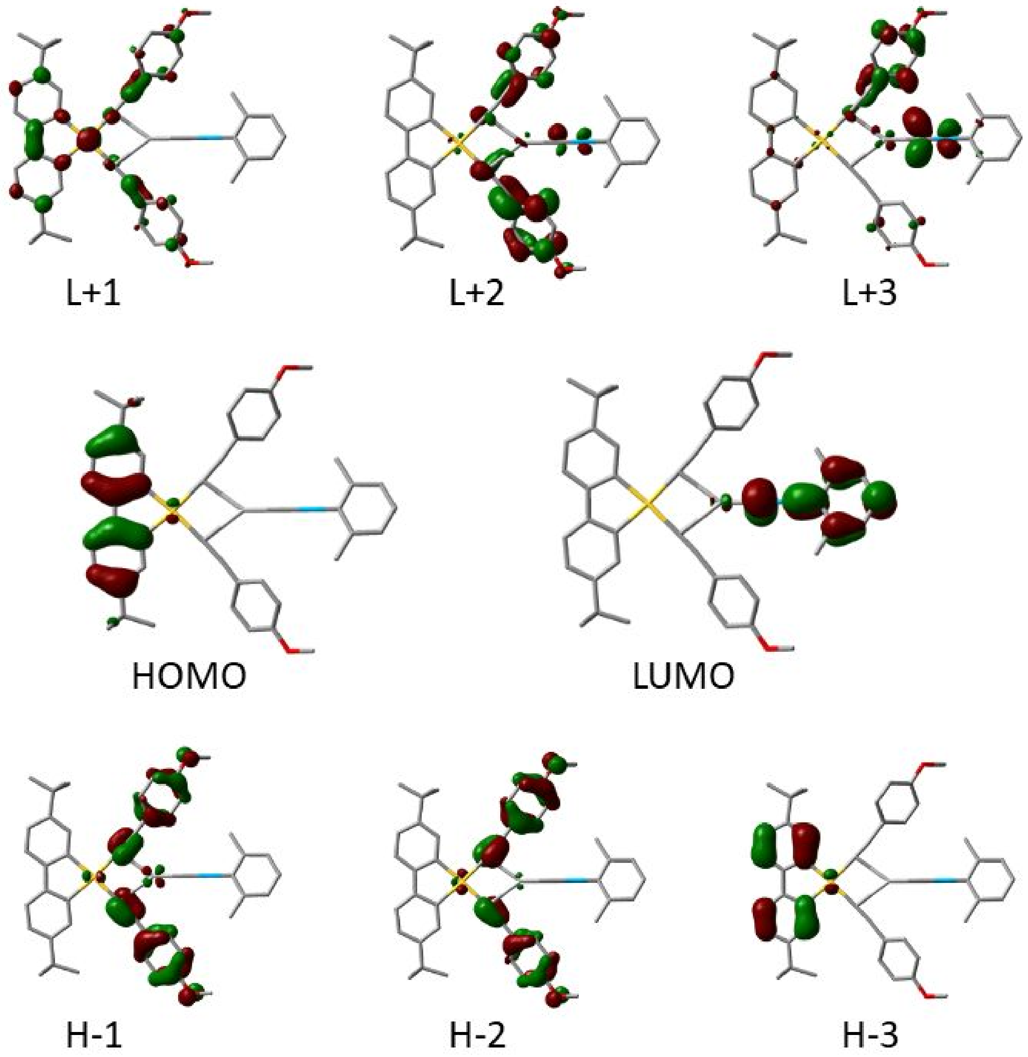

Figure S22. Selected frontier Molecular Orbitals for **4c** in the ground state.

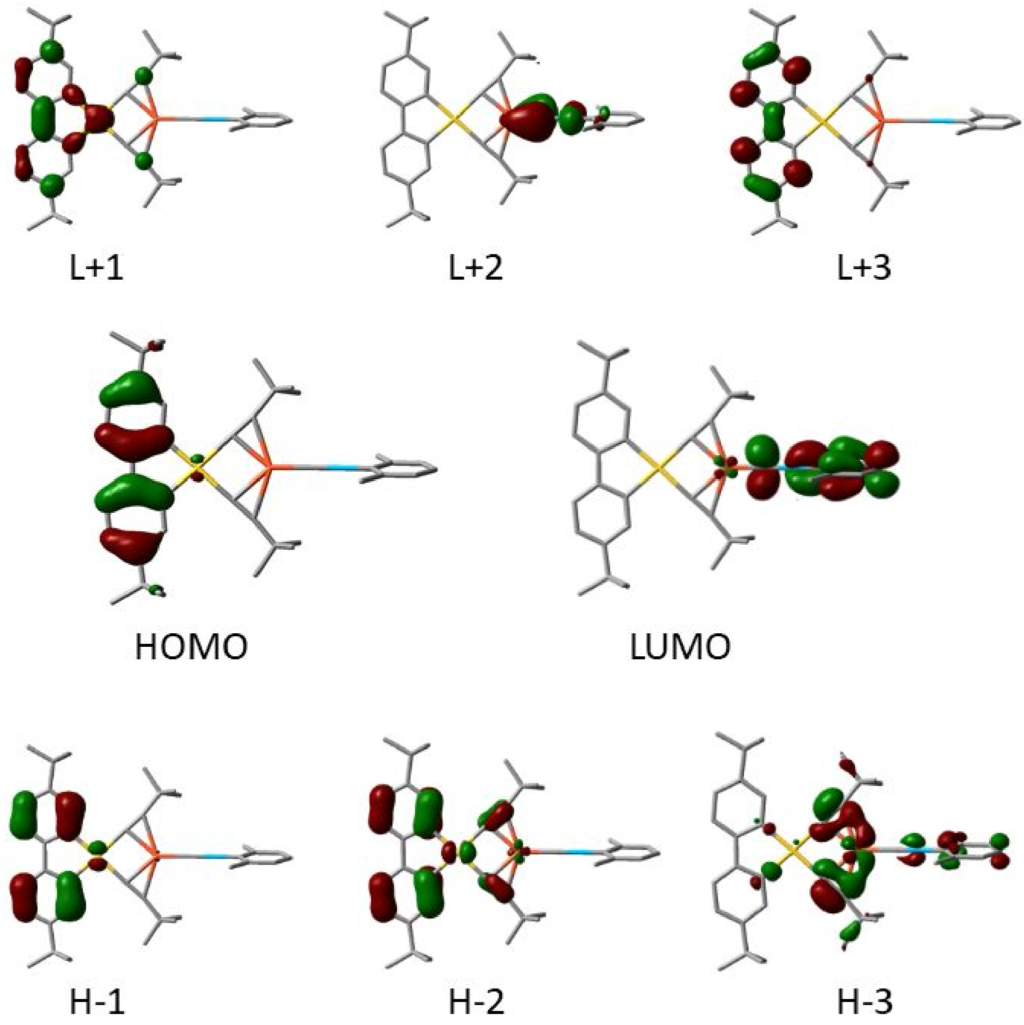

Figure S23. Selected frontier Molecular Orbitals for **5a** in the ground state.

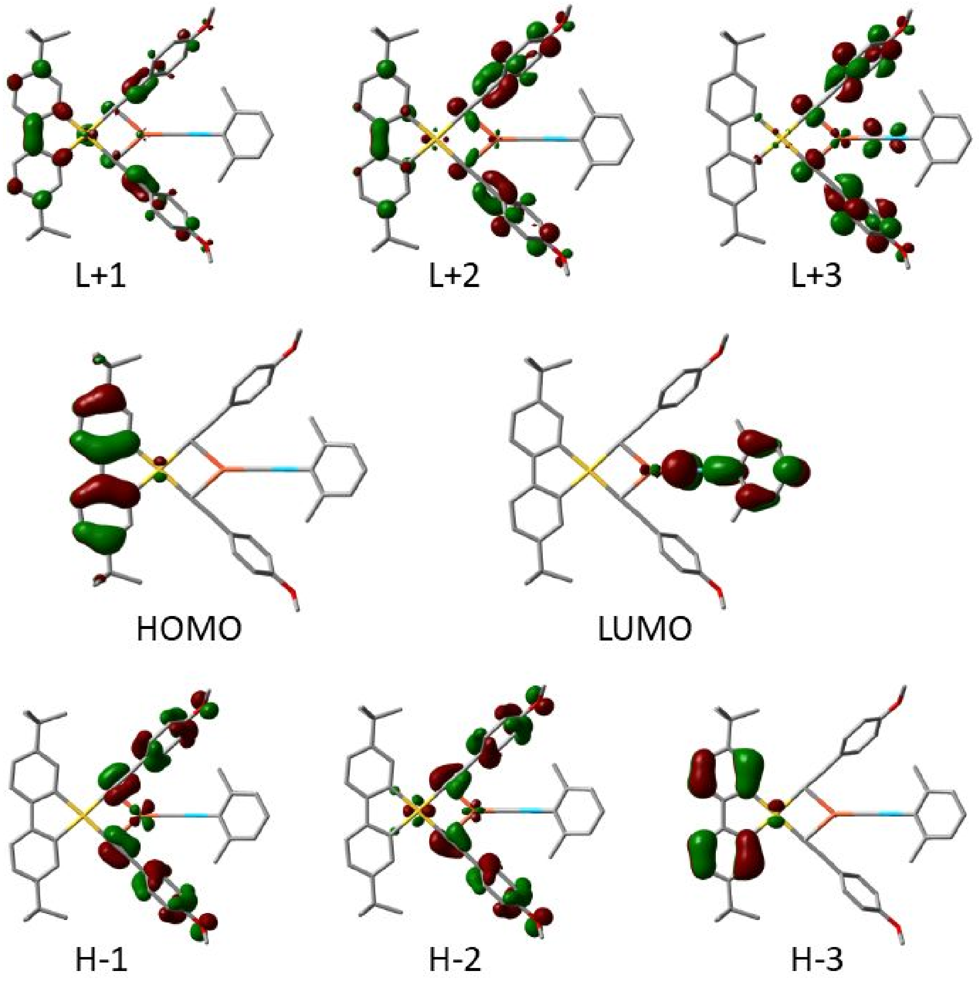

Figure S24. Selected frontier Molecular Orbitals for **5c** in the ground state.

Table S5. Selected vertical excitation energies singlets (S0) and first triplets computed by TDDFT/SCRF (CH_2Cl_2) with the orbitals involved for **1**

State	λ/nm	f	Transition (% Contribution)
T_1	431.9	-	H-1→L+1 (29%), HOMO→LUMO (17%), HOMO→L+2 (8%)
T_2	431.9	-	H-1→LUMO (17%), HOMO→L+1 (29%), H-1→L+2 (8%)
T_3	318.6	-	H-12→L+1 (5%), H-5→L+1 (7%), H-5→L+4 (6%), H-4→L+10 (7%), H-3→L+11 (8%), H-2→LUMO (9%), HOMO→L+14 (6%)
S_1	300.4	0.0003	H-1→L+1 (44%), HOMO→LUMO (44%)
**S_2**	**300.3**	**0.2090**	**H-1→LUMO (44%), HOMO→L+1 (44%)**
S_4	263.3	0.1855	H-4→L+1 (12%), H-1→L+2 (24%), HOMO→L+4 (28%)
S_6	252.5	0.0011	H-1→L+6 (39%), HOMO→L+3 (33%), HOMO→L+7 (13%)
S_8	245.4	0.1643	H-5→LUMO (19%), H-2→L+1 (33%), H-1→L+10 (9%), HOMO→L+11 (8%)
S_{11}	236.9	0.0093	H-9→LUMO (30%),r H-8→LUMO (17%), H-7→L+1 (29%)
S_{12}	234.6	0.1887	H-10→LUMO (34%), H-6→L+1 (23%), H-3→LUMO (9%)
S_{14}	230.0	1.1689	H-4→L+1 (23%), H-3→LUMO (19%), H-1→L+2 (11%), H-10→LUMO (8%)

Table S5 Continued. Selected vertical excitation energies singlets (S0) and first triplets computed by TDDFT/SCRF (CH_2Cl_2) with the orbitals involved for **2**

State	λ/nm	f	Transition (% Contribution)
T_1	433.8	-	H-1→LUMO (18%), H-1→L+2 (16%), HOMO→L+1 (30%)
T_2	433.8	-	H-1→L+1 (30%), HOMO→LUMO (19%), HOMO→L+2 (16%)
T_3	319.0	-	H-12→L+2 (5%), H-5→L+1 (7%), H-5→L+3 (5%), H-5→L+7 (5%), H-4→L+9 (5%), H-3→L+11 (7%), H-2→LUMO (9%), HOMO→L+13 (5%), HOMO→L+22 (5%)
S_1	303.0	0.0001	H-1→L+1 (46%), HOMO→LUMO (41%)
**S_2**	**302.9**	**0.1833**	**H-1→LUMO (41%), HOMO→L+1 (46%)**
S_4	263.6	0.1825	H-4→L+1 (13%), H-1→L+2 (24%), HOMO→L+3 (28%)
S_6	256.1	0.001	H-1→L+5 (40%), HOMO→L+4 (44%)
S_9	246.3	0.0607	H-9→LUMO (24%), H-7→L+1 (24%), H-2→L+1 (17%)
S_{10}	245.7	0.1715	H-7→L+1 (10%), H-5→LUMO (19%), H-2→L+1 (22%)
S_{11}	245.4	0.0351	H-10→LUMO (13%), H-8→LUMO (18%), H-6→L+1 (34%)
S_{14}	232.4	1.2974	H-4→L+1 (23%), H-3→LUMO (16%), H-1→L+2 (14%)

Table S5 Continued. Selected vertical excitation energies singlets (S0) and first triplets computed by TDDFT/SCRF (CH_2Cl_2) with the orbitals involved for **3a**

State	λ/nm	f	Transition (% Contribution)
T_1	427.8	-	HOMO→LUMO (18%), HOMO→L+1 (52%)
T_2	365.2	-	H-6→LUMO (34%), H-5→L+4 (22%), H-4→LUMO (14%)
T_3	315.1	-	H-3→L+7 (22%), HOMO→L+10 (10%), H-4→L+3 (9%), H-1→L+3 (8%), H-8→L+1 (7%)
**S_1**	**299.6**	**0.0927**	**HOMO→LUMO (59%), HOMO→L+1 (32%)**
S_2	263.1	0.0941	H-3→L+1 (14%), HOMO→L+1 (13%), HOMO→L+3 (47%)
S_5	253.3	0.1817	H-1→LUMO (72%)
S_6	247.0	0.0720	H-6→L+4 (10%), H-5→LUMO (65%), H-5→L+1 (10%)
S_7	240.6	0.6428	H-4→LUMO (35%), H-3→LUMO (10%), HOMO→L+7 (9%), H-6→LUMO (6%)
S_8	235.3	0.3387	H-3→LUMO (20%), HOMO→L+1 (17%), H-4→LUMO (8%), HOMO→LUMO (8%)
S_9	233.9	0.2691	H-6→LUMO (39%), H-4→LUMO (11%), HOMO→L+7 (12%)
S_{12}	223.6	0.3363	H-3→LUMO (16%), HOMO→LUMO (19%), HOMO→L+3 (24%), H-2→L+2 (7%), HOMO→L+6 (7%)

Table S5 Continued. Selected vertical excitation energies singlets (S0) and first triplets computed by TDDFT/SCRF (CH_2Cl_2) with the orbitals involved for **4c**

State	λ/nm	f	Transition (% Contribution)
T_1	427.1	-	HOMO→L+1 (42%), HOMO→L+4 (26%)
T_2	384.5	-	H-2→L+1 (11%), H-2→L+2 (14%), H-2→L+3 (10%), H-1→L+1 (11%)
T_3	384.0	-	H-2→L+1 (10%), H-2→L+2 (15%), H-1→L+1 (10%), H-1→L+2 (26%)
**S_1**	**292.4**	**0.1022**	**HOMO→L+1 (73%), HOMO→L+4 (10%)**
S_2	260.8	0.0628	H-3→L+1 (16%), HOMO→L+4 (21%), HOMO→L+8 (29%)
S_3	259.4	0.4448	H-2→L+2 (14%), H-1→LUMO (17%), H-1→L+1 (39%)
S_4	258.6	0.9762	H-2→L+1 (37%), H-1→L+2 (26%)
S_5	248.5	0.0135	H-2→L+6 (32%), H-1→L+3 (11%), H-1→L+7 (15%)
S_6	248.1	0.1868	H-2→L+7 (17%), H-1→L+6 (32%)
S_8	244.4	0.0927	H-8→L+5 (11%), H-7→LUMO (47%), HOMO→L+10 (20%)

S_9	243.8	0.3324	H-1→LUMO (48%), H-1→L+1 (10%)
S_{13}	229.9	0.3130	H-5→L+1 (24%), H-3→L+1 (20%)
S_{14}	228.8	0.5743	H-8→LUMO (54%)

Table S5 Continued. Selected vertical excitation energies singlets (S0) and first triplets computed by TDDFT/SCRF (CH_2Cl_2) with the orbitals involved for **5a**

State	λ/nm	f	Transition (% Contribution)
T_1	424.0	-	HOMO→L+1 (59%), HOMO→L+3 (13%)
T_2	365.3	-	H-8→LUMO (40%), H-6→L+4 (23%), H-3→LUMO (24%)
T_3	314.0	-	H-7→L+1 (10%), H-2→L+3 (17%), H-1→L+9 (22%), HOMO→L+14 (16%)
**S_1**	**287.2**	**0.2042**	**HOMO→L+1 (87%)**
S_2	261.0	0.0817	H-1→L+1 (20%), HOMO→L+3 (62%)
S_3	248.6	0.2904	H-3→LUMO (41%), H-3→L+2 (35%)
S_4	244.0	0.0427	H-8→L+4 (12%), H-6→LUMO (75%)
S_5	242.0	0.0175	HOMO→L+5 (50%)
S_6	241.3	0.1630	H-4→LUMO (12%), H-4→L+2 (12%), H-3→LUMO (11%), H-3→L+2 (12%), HOMO→L+5 (24%)
S_7	241.0	0.3273	H-4→LUMO (16%), H-3→LUMO (14%), H-3→L+2 (22%)
S_{10}	226.7	0.8597	H-1→L+1 (59%), HOMO→L+3 (27%)
S_{11}	226.4	0.0386	H-4→L+1 (54%)

Table S5 Continued. Selected vertical excitation energies singlets (S0) and first triplets computed by TDDFT/SCRF (CH_2Cl_2) with the orbitals involved for **5c**

State	λ/nm	f	Transition (% Contribution)
T_1	430.7	-	HOMO→L+1 (45%), HOMO→L+2 (24%)
T_2	378.4	-	H-2→L+3 (22%), H-1→L+1 (20%), H-1→L+2 (28%)
T_3	372.0	-	H-2→L+1 (14%), H-2→L+2 (21%), H-1→L+3 (30%)
**S_1**	**295.1**	**0.1347**	**HOMO→L+1 (71%), HOMO→L+2 (20%)**
S_2	276.0	0.0882	H-1→LUMO (72%)
S_3	260.2	0.1175	H-1→L+1 (23%), H-1→L+2 (10%), HOMO→L+7 (29%)
S_4	259.9	0.6195	H-1→L+1 (24%), H-1→L+2 (13%), HOMO→L+7 (23%)
S_5	252.9	0.1035	H-2→LUMO (30%), H-2→L+1 (12%), H-1→L+3 (11%)

S_7	246.2	0.1878	H-2→L+1 (13%), H-2→L+5 (11%), H-1→L+4 (12%), H-1→L+8 (15%)
S_9	245.1	0.1521	H-5→LUMO (25%), H-2→L+1 (12%), H-1→L+3 (10%)
S_{12}	237.5	0.5342	H-8→LUMO (18%), H-7→LUMO (23%), H-6→LUMO (11%)
S_{13}	234.1	0.1546	H-1→L+2 (13%), H-8→L+1 (8%), H-6→L+1 (9%), H-1→L+1 (9%)
S_{15}	230.4	0.3125	H-5→L+1 (23%), H-2→L+2 (17%)

Table S6. Plots and composition (%) of the frontier MOs and spin density of the first triplet state for **1**.

SOMO	SOMO-1	Spin density
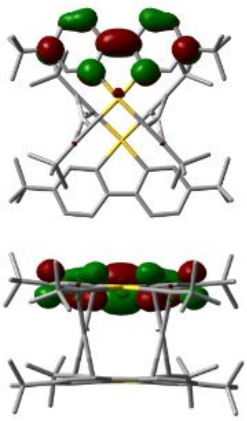	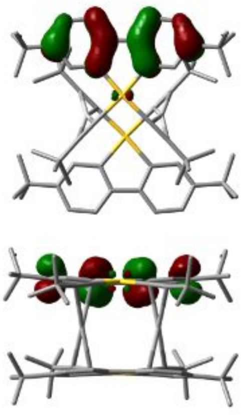	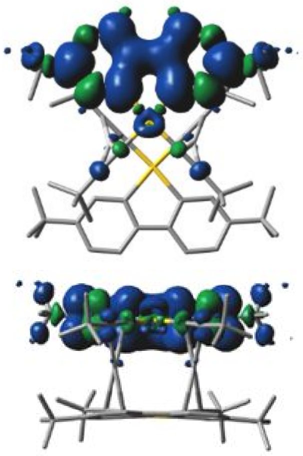
C^C^{1} 88%, C≡C^{1} 3% Au^{1} 7%, Ag^{1} 1%<br>C^C^{2} 0%, C≡C^{2} 0% Au^{2} 0%, Ag^{2} 1%	C^C1 99%, C≡C^{1} 0% Au^{1} 1%, Ag^{1} 0%<br>C^C^{2} 0%, C≡C^{2} 0% Au^{2} 0%, Ag^{2} 0%	

Table S6. **Continued**. Plots and composition (%) of the frontier MOs and spin density of the first triplet state for **2**.

SOMO	SOMO-1	Spin density
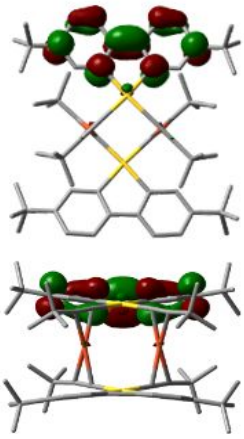	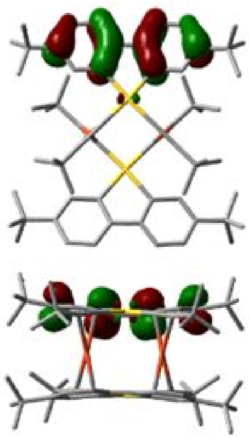	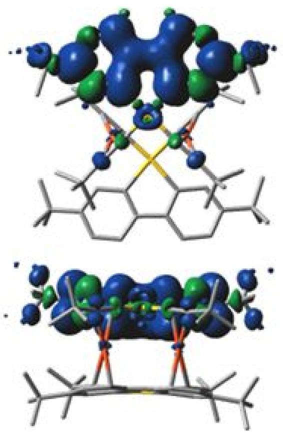
C^C^{1} 88%, C≡C^{1} 3%<br>Au^{1} 7%, Cu^{1} 1%<br>C^C^{2} 0%, C≡C^{2} 1%<br>Au^{2} 0%, Cu^{2} 1%	C^C^{1} 98%, C≡C^{1} 0%<br>Au^{1} 1%, Cu^{1} 0%<br>C^C^{2} 0%, C≡C^{2} 0%<br>Au^{2} 0%, Cu^{2} 0%	

Table S6 Continued. Plots and composition (%) of the frontier MOs and spin density of the first triplet state for **3a**.

SOMO	SOMO-1	Spin density
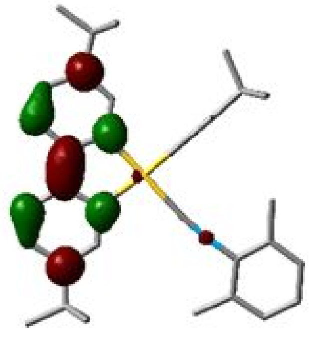	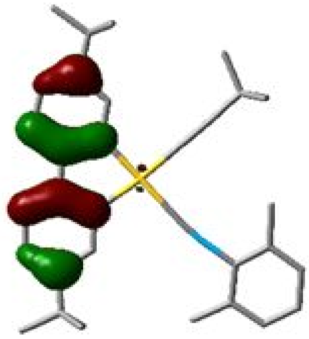	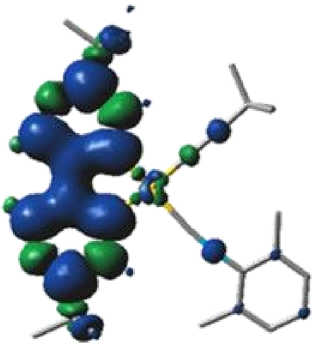
C^C 89%, C≡C 1%, C≡N 3%, Au 7%	C^C 99%, C≡C 0%, C≡N 0%, Au 1%	

Table S6 Continued. Plots and composition (%) of the frontier MOs and spin density of the first triplet state for **4c**.

SOMO	SOMO-1	Spin density
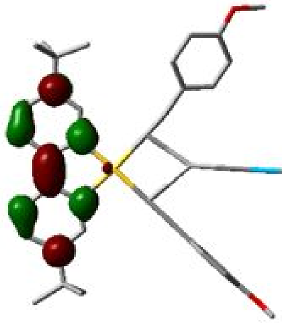	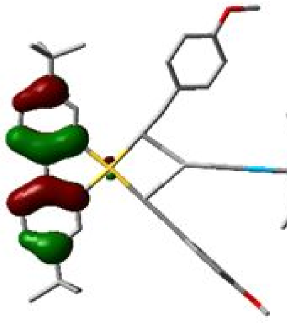	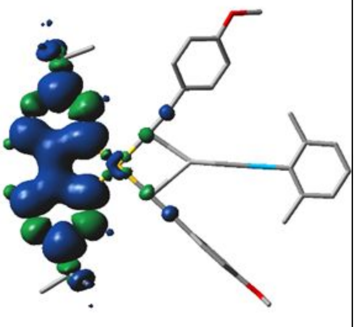
C^C 92%, C≡C 2%, Au 6% Ag 0%, C≡N 0%	C^C 99%, C≡C 0%, Au 1% Cu 0%, C≡N 0%	

Table S6 Continued. Plots and composition (%) of the frontier MOs and spin density of the first triplet state for **5a**.

SOMO	SOMO-1	Spin density
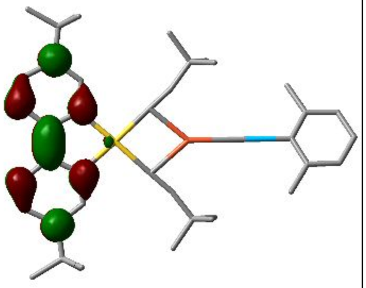	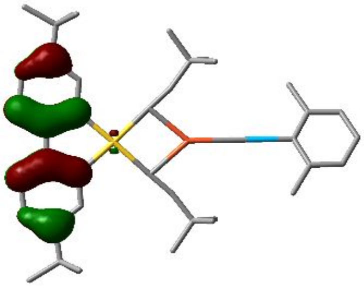	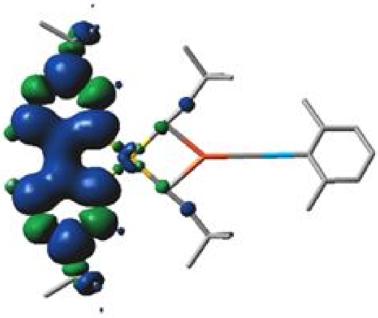
C^C 93%, C≡C 2%, Au 6% Cu 0%, C≡N 0%	C^C 99%, C≡C 0%, Au 1% Cu 0%, C≡N 0%	

Table S6 Continued. Plots and composition (%) of the frontier MOs and spin density of the first triplet state for **5c**.

SOMO	SOMO-1	Spin density
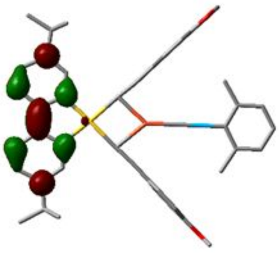	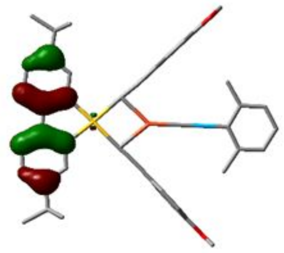	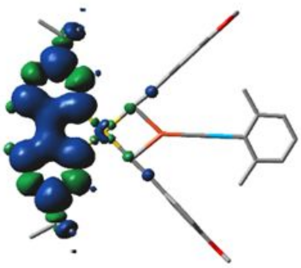
C^C 98%, C≡C 2%, Au 0%<br>Cu 0%, C≡N 0%	C^C 100%, C≡C 0%, Au 0%<br>Cu 0%, C≡N 0%	

References.

S1. *Programs CrysAlisPro*, Oxford Diffraction Ltd., Abingdon, UK (2018).

S2. Sheldrick, G. M. A short history of SHELX. *Acta Cryst.* **2008**, *A64*, 112. G. M. Sheldrick, Programs for crystal structure determination (SHELXT), Acta Cryst. (**2015**) A71, 3-8, and refinement (SHELXL), Acta Cryst. (**2008**) A64, 112-122 and (**2015**) C71, 3-8.

S3 Spek, A. L. (2006) PLATON – A Multipurpose Crystallographic Tool, Utrecht University, Utrecht, The Netherlands. A. L. Spek, *Acta Cryst.* **1990**, *A46*, C34

S4 Farrugia, L. J. WinGX. *J. Appl. Crystallogr.* **1999**, *32*, 837.

S5. 'International Tables for X-ray Crystallography', Kluwer Academic Publishers, Dordrecht. Vol. C. 1992, pp. 500, 219 and 193.

S6. Gaussian 16, Revision A.03, M. J. Frisch et al. Gaussian, Inc., Wallingford CT, 2016.

S7. O'Boyle, N. M.; Tenderholt, A. L.; Langner, K. M., Cclib: a library for package-independent computational chemistry algorithms. *J. Comput. Chem*. **2008**, *29*, 839-845.

S8. (a) Becke, A. D., Density-functional exchange-energy approximation with correct asymptotic behavior. *Phys. Rev. A* **1988**, *38*, 3098-3100. (b) Becke, A. D., Density-functional thermochemistry. III. The role of exact exchange. *J. Chem. Phys*. **1993**, *98*, 5648-5652. (c) Parr, R. G., Pariser, R. The Parameter I - A in Electronic Structure Theory in Concepts and Methods in Modern Theoretical Chemistry: Electronic Structure and Reactivity. **2016**, p 431 – 440 CRC Press. ISBN 978-146650531-5 and references therein.

S9 (a) T. Yanai, D.P. Tew, N.C. Handy, A new hybrid exchange–correlation functional using the Coulomb-attenuating method (CAM-B3LYP). *Chem. Phys. Lett.* **2004**, *393*, 51. (b) J.-D. Chai, M. Head-Gordon, Long-range corrected double-hybrid density functionals. *J. Chem. Phys.* **2009**, *131*, 174105-1.

S10 (a) F. Jensen, J. Chem. Theory Comput. Describing anions by density functional theory: fractional electron affinity. **2010**, *6*, 2726; (b) K. S. Thanthiriwatte, E. G. Hohenstein, L. A. Burns and C. D. Sherrill, Assessment of the performance of DFT and DFT-D methods for describing distance dependence of hydrogen-bonded interactions. *J. Chem. Theory Comput.* **2011**, *7*, 88.

S11 Wadt, W. R.; Hay, P. J., Ab initio effective core potentials for molecular calculations. Potentials for the transition metal atoms Sc to Hg. *J. Chem. Phys.* **1985**, *82*, 284-298.

S12. Barone, V.; Cossi, M., Quantum calculation of molecular energies and energy gradients in solution by a conductor solvent model. *J. Phys. Chem. A*. **1998**, *102*, 1995-2001.
